# Provision and uptake of routine antenatal services: a qualitative evidence synthesis

**DOI:** 10.1002/14651858.CD012392.pub2

**Published:** 2019-06-12

**Authors:** Soo Downe, Kenneth Finlayson, Özge Tunçalp, Ahmet Metin Gülmezoglu

**Affiliations:** University of Central LancashireResearch in Childbirth and Health (ReaCH) unitPrestonUKPR1 2HE; World Health OrganizationUNDP/UNFPA/UNICEF/WHO/World Bank Special Programme of Research, Development and Research Training in Human Reproduction (HRP), Department of Reproductive Health and Research20 Avenue AppiaGenevaSwitzerland1211

**Keywords:** Female, Humans, Pregnancy, Health Personnel, Health Personnel/psychology, Health Services Accessibility, Pregnant People, Pregnant People/psychology, Quality of Health Care, Attitude of Health Personnel, Culture, Developed Countries, Fraud, Health Care Costs, Health Facility Environment, Personnel Staffing and Scheduling, Postpartum Period, Prenatal Care, Prenatal Care/economics, Prenatal Care/methods, Prenatal Care/organization & administration, Prenatal Care/statistics & numerical data, Qualitative Research, Sex Factors

## Abstract

**Background:**

Antenatal care (ANC) is a core component of maternity care. However, both quality of care provision and rates of attendance vary widely between and within countries. Qualitative research can assess factors underlying variation, including acceptability, feasibility, and the values and beliefs that frame provision and uptake of ANC programmes. This synthesis links to the Cochrane Reviews of the effectiveness of different antenatal models of care. It was designed to inform the World Health Organization guidelines for a positive pregnancy experience and to provide insights for the design and implementation of improved antenatal care in the future.

**Objectives:**

To identify, appraise, and synthesise qualitative studies exploring:

· Women’s views and experiences of attending ANC; and factors influencing the uptake of ANC arising from women’s accounts;

· Healthcare providers’ views and experiences of providing ANC; and factors influencing the provision of ANC arising from the accounts of healthcare providers.

**Search methods:**

To find primary studies we searched MEDLINE, Ovid; Embase, Ovid; CINAHL, EbscoHost; PsycINFO, EbscoHost; AMED, EbscoHost; LILACS, VHL; and African Journals Online (AJOL) from January 2000 to February 2019. We handsearched reference lists of included papers and checked the contents pages of 50 relevant journals through Zetoc alerts received during the searching phase.

**Selection criteria:**

We included studies that used qualitative methodology and that met our quality threshold; that explored the views and experiences of routine ANC among healthy, pregnant and postnatal women or among healthcare providers offering this care, including doctors, midwives, nurses, lay health workers and traditional birth attendants; and that took place in any setting where ANC was provided. We excluded studies of ANC programmes designed for women with specific complications. We also excluded studies of programmes that focused solely on antenatal education.

**Data collection and analysis:**

Two authors undertook data extraction, logged study characteristics, and assessed study quality. We used meta‐ethnographic and Framework techniques to code and categorise study data. We developed findings from the data and presented these in a 'Summary of Qualitative Findings' (SoQF) table. We assessed confidence in each finding using GRADE‐CERQual. We used these findings to generate higher‐level explanatory thematic domains. We then developed two lines of argument syntheses, one from service user data, and one from healthcare provider data. In addition, we mapped the findings to relevant Cochrane effectiveness reviews to assess how far review authors had taken account of behavioural and organisational factors in the design and implementation of the interventions they tested. We also translated the findings into logic models to explain full, partial and no uptake of ANC, using the theory of planned behaviour.

**Main results:**

We include 85 studies in our synthesis. Forty‐six studies explored the views and experiences of healthy pregnant or postnatal women, 17 studies explored the views and experiences of healthcare providers and 22 studies incorporated the views of both women and healthcare providers. The studies took place in 41 countries, including eight high‐income countries, 18 middle‐income countries and 15 low‐income countries, in rural, urban and semi‐urban locations. We developed 52 findings in total and organised these into three thematic domains: socio‐cultural context (11 findings, five moderate‐ or high‐confidence); service design and provision (24 findings, 15 moderate‐ or high‐confidence); and what matters to women and staff (17 findings, 11 moderate‐ or high‐confidence) The third domain was sub‐divided into two conceptual areas; personalised supportive care, and information and safety. We also developed two lines of argument, using high‐ or moderate‐confidence findings:

For women, initial or continued use of ANC depends on a perception that doing so will be a positive experience. This is a result of the provision of good‐quality local services that are not dependent on the payment of informal fees and that include continuity of care that is authentically personalised, kind, caring, supportive, culturally sensitive, flexible, and respectful of women’s need for privacy, and that allow staff to take the time needed to provide relevant support, information and clinical safety for the woman and the baby, as and when they need it. Women’s perceptions of the value of ANC depend on their general beliefs about pregnancy as a healthy or a risky state, and on their reaction to being pregnant, as well as on local socio‐cultural norms relating to the advantages or otherwise of antenatal care for healthy pregnancies, and for those with complications. Whether they continue to use ANC or not depends on their experience of ANC design and provision when they access it for the first time.

The capacity of healthcare providers to deliver the kind of high‐quality, relationship‐based, locally accessible ANC that is likely to facilitate access by women depends on the provision of sufficient resources and staffing as well as the time to provide flexible personalised, private appointments that are not overloaded with organisational tasks. Such provision also depends on organisational norms and values that overtly value kind, caring staff who make effective, culturally‐appropriate links with local communities, who respect women’s belief that pregnancy is usually a normal life event, but who can recognise and respond to complications when they arise. Healthcare providers also require sufficient training and education to do their job well, as well as an adequate salary, so that they do not need to demand extra informal funds from women and families, to supplement their income, or to fund essential supplies.

**Authors' conclusions:**

This review has identified key barriers and facilitators to the uptake (or not) of ANC services by pregnant women, and in the provision (or not) of good‐quality ANC by healthcare providers. It complements existing effectiveness reviews of models of ANC provision and adds essential insights into why a particular type of ANC provided in specific local contexts may or may not be acceptable, accessible, or valued by some pregnant women and their families/communities. Those providing and funding services should consider the three thematic domains identified by the review as a basis for service development and improvement. Such developments should include pregnant and postnatal women, community members and other relevant stakeholders.

## Plain language summary

**Provision and uptake of routine antenatal services**

**What is the aim of this review?**

The aim of this Cochrane qualitative evidence synthesis is to explore women’s and healthcare workers’ views and experiences of antenatal care. We collected and analysed all relevant qualitative studies to answer this question, and include 85 studies.

The synthesis links to the Cochrane Reviews of the effectiveness of different antenatal models of care. The synthesis was designed to inform the World Health Organization guidelines for a positive pregnancy experience.

**Key messages**

Three areas of antenatal care are important to both women and service providers in all regions of the world. These are: the need to recognise and take account of the socio‐cultural context in which care is provided; the need to ensure that service design and provision are appropriate, accessible, acceptable and of high quality: and that what matters to women and staff is personalised supportive care, information, and safety.

**What was studied in this review?**

Antenatal care is the health care women get while they are pregnant. During antenatal care visits, pregnant women are provided with support, reassurance, and information about pregnancy and birth, as well as tests and examinations to see if they and their baby are healthy. If any issues or problems are discovered, these can be managed during the clinic visit. If needed, women can be referred to other care providers. Different types of healthcare workers can give antenatal care. These include midwives, doctors, nurses, and, sometimes, traditional birth attendants.

The World Health Organization recommends that all pregnant women get antenatal care, but pregnant women do not always use this care. This may be because they do not think it is important, or because they cannot get to the healthcare facility. It may also be because the antenatal care they receive is of poor quality or because they are badly treated when they are there. By looking at studies of women’s and healthcare workers’ views and experiences of antenatal care, we aimed to learn more about what might help women to use antenatal care, and what might stop them using it.

**What are the main findings of this review?**

We include 85 studies in our synthesis. Forty‐six studies explored the views and experiences of women who were pregnant or who had recently given birth. 17 studies explored the views and experiences of healthcare providers, including lay or community health workers, and 22 studies included the views of both women and healthcare providers. The studies took place in eight high‐income countries, 18 middle‐income countries and 12 low‐income countries, in rural and urban locations.

Our findings suggest that women use antenatal care if they find it is a positive experience that fits with their beliefs and values, is easy for them to access, affordable, and treats them as an individual. They want care that helps them to feel that they and their baby are safe, and that is provided by kind, caring, culturally sensitive, flexible, and respectful staff that have time to give them support and reassurance about the health and well‐being of them and their babies. They also value tests and treatments that are offered when they need them, and information and advice that is relevant to them.

Our findings also suggest that healthcare staff want to be able to offer this kind of care. They would like to work in antenatal services that are properly funded, and that give them proper support, pay, training and education. They believe this will help them to have enough time to treat each pregnant woman as an individual, and to have the knowledge, skills resources and equipment to do their job well.

**How up‐to‐date is this review?**

The review authors searched for studies that had been published up to February 2019.

## Summary of findings

**Summary of findings for the main comparison:** Summary of qualitative findings ‐ Socio‐cultural context

**SOCIO‐CULTURAL CONTEXT**				
**Summary of review finding**		**Studies contributing to the review finding**	**CERQual assessment of confidence in the evidence**	**Explanation of CERQual assessment**
**Influence of traditional beliefs**				
Women	**W1. Influence of traditional beliefs** Women in many LMICs hold a range of diverse medical, spiritual and supernatural beliefs which may limit their engagement with ANC services. In these contexts biomedical approaches to health care were not culturally normative and women sometimes turned to TBAs or traditional healers for remedies to treat a variety of pregnancy‐related symptoms or to protect against or dispel the effects of 'evil spirits'. Where healthcare providers developed an understanding of and a respect for these traditional beliefs women were more likely to engage with ANC providers	14 studies^a^	Moderate confidence	Finding downgraded because of concerns about relevance. Likely to be more relevant in LMICs
	**W2. Influence of others** Engagement with ANC can be influenced by a variety of family members and community representatives who may encourage attendance (husband, mother, community health worker or the local TBA) or discourage access (mothers‐in‐law) (Pakistan, Nepal, Afghanistan and Bangladesh)	11 studies^b^	Moderate confidence	Finding downgraded because of concerns about relevance. Likely to be more relevant in LMICs
**Influence of local beliefs and traditional maternity practices**				
Providers	**P1. Co‐operation with influential community members** Where providers were able to co‐operate effectively with influential tribal elders and TBAs this was viewed as a facilitator to ANC access. Where there was a lack of understanding and co‐operation, especially with TBAs, this was perceived as having a detrimental effect on women's willingness to engage with ANC services	5 studies^c^	Moderate confidence	Finding downgraded because of concerns about relevance. Likely to be more relevant in LMICs
	**P2. Traditional, societal and community norms, practices and beliefs** Providers believe that women do not always engage with ANC because of a variety of traditional views about maternity care, including superstitious beliefs, the shame associated with being pregnant, the perception that pregnancy is a healthy state and their preference to be seen by a TBA or doctor	11 studies^d^	Moderate confidence	Finding downgraded because of concerns around methodology and coherence
**Pregnancy as a healthy state**				
Women	**W3. Pregnancy seen as a normal event** In a number of countries women and their communities viewed pregnancy as a normal, healthy state of being and saw no reason to attend a health facility when they did not perceive themselves to be ill or unwell	16 studies^e^	High confidence	Likely to be a factor in a variety of settings and contexts, particularly in LMICs
**Selective use of ANC**				
Women	**W4. Confirmation of pregnancy** Visiting an antenatal clinic to have a pregnancy test was seen as a clean and reliable way of confirming a pregnancy and encouraged attendance at ANC facilities. However, some women viewed the pregnancy test as the only reason to visit an ANC provider and attended only once to confirm their pregnancy	6 studies^f^	Low confidence	Finding downgraded because of concerns about relevance and coherence. Likely to be relevant in LICs
	**W5. Only visit clinic to get an ANC card** In several LMICs women only visit the clinic to get an ANC card and do not return for further appointments. The ANC card is valued by women as it is seen as an insurance policy allowing access to the hospital in the event of a pregnancy complication, and is often used by providers as a 'passport' to guarantee admission to a hospital at the time of delivery	5 studies^g^	Low confidence	Finding downgraded because of concerns about relevance and coherence. Likely to be relevant in specific African LMICs
**Gender issues**				
Women	**W6. Financial dependence on husband** In a number of traditional contexts women have to ask their husbands for money to attend ANC and this can act as a barrier if husbands are particularly poor or if they are unsupportive of ANC	6 studies^h^	Low confidence	Finding downgraded because of concerns about relevance and coherence
	**W7. Shame and embarrassment** In some LMICs there is a sense of shame attached to being pregnant because of its association with sex (Pakistan and Bangladesh). In other settings a sense of shame may be felt by women following criticism from health providers about the size of their families, whilst in South America women felt shame and embarrassment about routine physical examinations	6 studies^i^	Low confidence	Finding downgraded because of concerns about relevance and coherence
	**W8. Gender of health care provider** Women prefer to be seen by a female healthcare provider during ANC visits. This view seems to be based on the assumption that women have a better understanding and mutual affinity with pregnancy and child birth compared to men	7 studies^j^	Low confidence	Finding downgraded because of concerns about relevance and coherence
	**W9. Women's freedom of movement** Due to cultural or religious beliefs in some countries, the need for women to be accompanied in public places can limit engagement with ANC services as there are not always people willing or available to go with them	2 studies^k^	Very low confidence	Finding downgraded because of concerns about adequacy of data, relevance and coherence. Likely to be a factor in specific contexts only


ANC: antenatal care: HIC: high‐income countries; HMICs: high‐ and ‐middle‐income countries: LIC: low‐income country; LMICs: low‐ and middle‐income countries ^a^Agus 2012 (Indonesia); Chapman 2003 (Mozambique); Choudhury 2011 (Bangladesh); Dako‐Gyeke 2013 (Ghana); Family Care International 2003 (Kenya); Mahiti 2015 (Tanzania); Matsuoka 2010 (Cambodia); Mayca 2009 (Peru); Mumtaz 2007 (Pakistan); Rath 2010 (India); Stokes 2008 (Gambia); Sychareun 2016(Lao PDR); Thwala 2011 (Swaziland); Titaley 2010 (Indonesia). ^b^Andrew 2014 (PNG); Ayala 2013 (Peru); Chowdhury 2003 (Bangladesh); Dako‐Gyeke 2013 (Ghana); Griffiths 2001 (India); Mrisho 2009 (Tanzania); Mumtaz 2007 (Pakistan); Munguambe 2016 (Mozambique); Rahmani 2013 (Afghanistan); Simkhada 2010 (Nepal); Stokes 2008 (Gambia). ^c^Chimezie 2013 (Nigeria); Bradley 2012 (Ethiopia); Franngard 2006 (Uganda); Graner 2010 (Vietnam); Manithip 2013 (Laos). ^d^Chimezie 2013 (Nigeria); Dako‐Gyeke 2013 (Ghana); Graner 2010 (Vietnam); Heaman 2015 (Canada); Khoso 2016 (Pakistan). LeMasters 2018 (Romania); Mayca 2009 (Peru); Mugo 2018 (South Sudan); Munguambe 2016 (Mozambique); Rahmani 2013 (Afghanistan); Titaley 2010 (Indonesia). ^e^Agus 2012 (Indonesia); Andrew 2014 (PNG); Chapman 2003 (Mozambique); Choudhury 2011 (Bangladesh); Chowdhury 2003 (Bangladesh); Coverston 2004 (Argentina); Haddrill 2014 (UK); Kabakian‐Khasholian 2000 (Lebanon); Khoso 2016 (Pakistan); LeMasters 2018 (Romania); Maputle 2013 (South Africa); Matsuoka 2010 (Cambodia); Mumtaz 2007 (Pakistan); Myer 2003 (South Africa); Rahmani 2013 (Afghanistan); Titaley 2010 (Indonesia). ^f^Andrew 2014 (PNG); Choudhury 2011 (Bangladesh); Chowdhury 2003 (Bangladesh); Family Care International 2003 (Kenya); Larsson 2017(Sweden); Mrisho 2009 (Tanzania). ^g^Abrahams 2001 (South Africa); Family Care International 2003 (Kenya); Mrisho 2009 (Tanzania); Myer 2003 (South Africa); Thwala 2011 (Swaziland). ^h^Chapman 2003 (Mozambique);Choudhury 2011 (Bangladesh); Chowdhury 2003 (Bangladesh); Østergaard 2015 (Burkina Faso); Rahmani 2013 (Afghanistan); Umeora 2008 (Nigeria). ^i^Andrew 2014 (Papua New Guinea); Chowdhury 2003 (Bangladesh); Coverston 2004 (Argentina); Mayca 2009 (Peru); Mumtaz 2007 (Pakistan); Walburg 2014 (France). ^j^Armstrong 2005 (Australia); Ayala 2013 (Peru); Kabakian‐Khasholian 2000 (Lebanon); Khoso 2016 (Pakistan); Maputle 2013 (South Africa); Stokes 2008 (Gambia); Walburg 2014 (France). ^k^Chowdhury 2003 (Bangladesh); Mumtaz 2007 (Pakistan).

**Summary of findings 2:** Summary of qualitative findings ‐ Service philosophy, design and provision

**SERVICE PHILOSOPHY, DESIGN and PROVISION**				
**Summary of review finding**		**Studies contributing to the review finding**	**CERQual assessment of confidence in the evidence**	**Explanation of CERQual assessment**
**Local infrastructure**				
Women	**W10. Poor infrastructure** Some women were unable or unwilling to visit a clinic because of the poor infrastructure. This was particularly pertinent in rural areas where the prospect of making long journeys (sometimes on foot) presented a variety of potential problems or dangers	6 studies^a^	Moderate confidence	Finding downgraded because of concerns about relevance and coherence
	**W11. Proximity of clinic** In certain circumstances the convenience of having an ANC clinic close by encouraged ANC attendance, but for most women the inconvenience of having to visit a clinic in a distant location or in an unfamiliar part of town acted as a barrier to access	10 studies^b^	Moderate confidence	Finding downgraded because of concerns about relevance and coherence. Likely to be a negative factor in rural locations
Providers	**P3. Proximity of Clinic** Health professionals believed that having a clinic close by acted as an incentive to ANC access for most women	5 studies^c^	Low confidence	Finding downgraded because of concerns about adequacy of data, relevance and coherence. Based on 1 study
	**P4. Availability of transport** Providers believed that the accessibility and availability of local transport acted as a barrier (where services were poor) or a facilitator (where services were good) to ANC attendance	9 studies^d^	Moderate confidence	Finding downgraded because of concerns about adequacy of data, relevance and coherence. Likely to be a negative factor in rural locations especially in LMICs
**Cost of services**				
Women	**W12. Indirect cost of services** In the vast majority of countries ANC is provided free of charge but in many contexts the indirect costs associated with transport to and from the clinic, the purchase of additional medicines and the potential loss of income associated with clinic attendance all acted as a barrier to ANC engagement	22 studies^e^	High confidence	Likely to be a negative factor in a range of settings and contexts, especially in LMICs
Providers	**P5. Indirect costs of ANC** Providers believed that some women on particularly low incomes ca not afford to come to ANC because of the additional costs associated with attendance (transport and medicines) or because of the loss of income incurred as a result of being away from work	13 studies^f^	High confidence	Finding likely to be a factor in a range of settings and contexts
	**P6. Staff corruption** Providers in one location supplemented their salaries by selling medicines and equipment that were supposed to be provided to women free of charge	2 studies^g^	Very low confidence	Finding downgraded because of concerns about adequacy of data, relevance and coherence. Based on 1 study
**Clinic environment**				
Women	**W13. Need for privacy** The opportunity to hold private conversations with healthcare professionals was seen as an important aspect of ANC and, in situations where this was not possible (e.g. group ANC), the lack of privacy occasionally acted as a barrier to further engagement	4 studies^h^	Low confidence	Finding downgraded because of concerns about relevance and coherence. Limited number of studies from HICs only
	**W14. Waiting times** In a number of countries women had to wait for long periods of time before being seen by a health professional. For some women, especially in LMICs, these long waits meant a loss of vital income and discouraged further engagement with ANC services	11 studies^i^	Moderate confidence	Finding downgraded because of concerns about relevance and coherence
	**W15. Time spent with health professional** Women welcome a regular series of ANC appointments and want to spend time with a health professional at each visit, discussing various aspects of their pregnancy without feeling rushed. In this regard the group model of ANC is appreciated because of the unhurried nature of the approach and the opportunity to spend more time with a health professional at each visit	15 studies^j^	High confidence	Finding likely to be a factor in a range of settings and contexts
Providers	**P7. Condition of clinic** Providers in Sub‐Saharan Africa felt that clinics were in a very poor condition and were not amenable to the delivery of quality ANC. They cited a lack of running water or electricity, no phone lines and dirty rooms as specific concerns	6 studies^k^	Low confidence	Finding downgraded because of concerns about relevance and coherence. Finding limited to rural African locations
	**P8. Privacy** Providers felt that the opportunity to hold private conversations with women was an important ingredient of quality ANC. However, in a number of different contexts they felt that overcrowded clinics coupled with a lack of physical space meant that privacy was often compromised and acted as a barrier to the delivery of quality ANC	8 studies^l^	Moderate confidence	Finding downgraded because of concerns about relevance and coherence
	**P9. Time with women** Because of staff shortages and in some instances the high demand for services, providers felt they did not have enough time to deliver an informative, woman‐centred ANC service to women	13 studies^m^	High confidence	Finding likely to be a factor in a range of settings and contexts
**Organisation of services**				
Women	**W16. Disorganised services** Some women felt they were given confusing and inconsistent messages around the timing and content of ANC services, which discouraged further visits	7 studies^n^	Low confidence	Finding downgraded because of concerns around adequacy of data, methodology and coherence. 2 of the 4 studies came from rural areas of Uganda
	**W17. Flexibility of appointments** Women reported that they did not like rigid appointment systems and appreciated a flexible approach to service delivery including the provision of drop‐in clinics, out‐of‐hours services, home visits and the ability to contact midwives directly	9 studies^o^	Moderate confidence	Finding downgraded because of concerns around coherence
Providers	**P10. Organisation of services** Some providers felt the organisation of ANC was haphazard and unco‐ordinated. They felt the timing and availability of education sessions and appointments were not designed to meet the needs of women and that health promotion programmes were often implemented simultaneously, leading to confusion and frustration amongst staff	8 studies^p^	Low confidence	Finding downgraded because of concerns around adequacy of data, methodology and coherence. 2 of the 3 studies came from rural areas of Uganda
	**P11. Flexibility of appointments** By offering a variety of appointment times and being flexible with their time, providers felt they were able to offer a more woman‐centred service, and in one study this led to shorter waiting times for women at the clinic. Where ANC appointments were viewed as being rigid and inflexible this was perceived to be a barrier to access	8 studies^q^	Moderate confidence	Finding downgraded because of concerns around coherence
**Resource issues and working conditions**				
Women	**W18. Lack of clinical resources** Women highlighted the lack of medicine and medical equipment at clinics as potential barriers to ANC access. Some clinics lacked basic supplies and women were asked to bring essential items (e.g. rubber gloves) to ANC appointments. Because of the perceived inadequacy at public health clinics women occasionally turned to private providers at additional cost	5 studies^r^	Low confidence	Finding downgraded because of concerns about relevance and coherence. Likely to be a factor in some LMICs
Providers	**P12. Shortage of staff** Health professionals from a wide variety of settings and contexts felt that their ability to deliver high‐quality ANC was restricted by a shortage of frontline staff	18 studies^s^	High confidence	Finding likely to be a factor in a range of settings and contexts
	**P13. Availability of resources** Providers believe that their ability to deliver ANC is restricted by the limited amount of resources available to them. Medicines, equipment and written information about ANC were cited as being either unavailable or in short supply. Providers in one rural location were able to purchase extra resources using income generated from selling food grown on clinic land	13 studies^t^	High confidence	Finding likely to be relevant in a range of LMICs, particularly in Sub‐Saharan Africa
	**P14. Staff working conditions** Health professionals felt that low salaries coupled with a heavy workload and a high staff turnover prevented them from delivering high‐quality ANC.	11 studies^u^	Moderate confidence	Finding downgraded because of concerns around coherence. No data from HICs
	**P15. Staff training** Health professionals felt they were not given sufficient training to carry out their role. Poor knowledge of standard ANC practices, an inability to deal with complications or a lack of understanding of cultural practices were all cited as examples. Providers also bemoaned the lack of opportunities for further training	12 studies^v^	High confidence	Finding likely to be a factor in a range of settings and contexts
	**P16. Need for management support** Health professionals wanted appropriate support from their managers and appreciated a positive, receptive and encouraging managerial style as opposed to a distant, uncommunicative and rigid approach	4 studies^w^	Low confidence	Finding downgraded because of concerns about adequacy of data, relevance and coherence
**(Over‐) emphasis on risk**				
Providers	**P17. Emphasis on risk** Some health professionals thought that the emphasis on risk‐focused screening and intervention, particularly around HIV and malaria, limited their ability to offer genuine care. This was often compounded by the pressure to achieve local, national or international targets and, with the limited time available, they sometimes fell short of meeting ANC recommendations	10 studies^x^	Moderate confidence	Finding downgraded because of concerns about relevance and coherence


ANC: antenatal care: HIC: high‐income countries; HMICs: high‐ and ‐middle‐income countries: LIC: low‐income country; LMICs: low‐ and middle‐income countries ^a^Mahiti 2015 (Tanzania); Mrisho 2009 (Tanzania); Munguambe 2016 (Mozambique); Rahmani 2013 (Afghanistan); Rath 2010 (India); Titaley 2010 (Indonesia). ^b^Cabral 2013 (Brazil); Griffiths 2001 (India); Khoso 2016 (Pakistan); Haddrill 2014 (UK); LeMasters 2018 (Romania); Matsuoka 2010 (Cambodia); Munguambe 2016 (Mozambique); Pretorius 2004 (South Africa); Simkhada 2010 (Nepal); Sword 2012 (Canada); ^c^Chimezie 2013 (Nigeria); Heaman 2015 (Canada); Hunter 2017 (Ireland); Miteniece 2018 (Georgia); Mugo 2018 (South Sudan); ^d^Andrew 2014 (Papua New Guinea); Baffour‐Awuah 2015 (Ghana); Bradley 2012 (Ethiopia); Heaman 2015 (Canada); Miteniece 2018 (Georgia); Mrisho 2009 (Tanzania); Mugo 2018 (South Sudan); Munguambe 2016 (Mozambique); Rahmani 2013 (Afghanistan). ^e^Abrahams 2001 (South Africa); Agus 2012 (Indonesia); Andrew 2014 (PNG); Choudhury 2011 (Bangladesh); Chowdhury 2003 (Bangladesh); Coverston 2004 (Argentina); Family Care International 2003 (Kenya); Griffiths 2001 (South Africa); Kabakian‐Khasholian 2000 (Lebanon); Khoso 2016 (Pakistan); Mahiti 2015 (Tanzania); Maputle 2013 (South Africa); Matsuoka 2010 (Cambodia); Mrisho 2009 (Tanzania); Mumtaz 2007 (Pakistan); Munguambe 2016 (Mozambique); Myer 2003 (South Africa); Rahmani 2013 (Afghanistan); Santos 2010 (Brazil); Simkhada 2010 (Nepal); Titaley 2010 (Indonesia); Umeora 2008 (Nigeria). ^f^Bradley 2012 (Ethiopia); Chimezie 2013 (Nigeria); Gheibizadeh 2016 (Iran); Graner 2010 (Vietnam); Heaman 2015 (Canada); Hunter 2017 (Ireland); LeMasters 2018 (Romania); Miteniece 2018 (Georgia);Molina 2011 (Colombia); Mugo 2018 (South Sudan); Munguambe 2016 (Mozambique); Rahmani 2013 (Afghanistan); Titaley 2010 (Indonesia). ^g^LeMasters 2018 (Romania); Rahmani 2013 (Afghanistan). ^h^Gheibizadeh 2016 (Iran); Hunter 2017 (Ireland); Novick 2011 (USA); Sword 2012 (Canada). ^i^Abrahams 2001 (South Africa); Ayala 2013 (Peru); Cardelli 2016 (Brazil); Chapman 2003 (Mozambique); Conrad 2012 (Uganda); Gheibizadeh 2016 (Iran); Hunter 2017 (Ireland); Mahiti 2015 (Tanzania); Pretorius 2004 (South Africa); Shabila 2014 (Iraq); Worley 2004 (New Zealand). ^j^Bessett 2010 (USA); Cabral 2013 (Brazil); De Castro 2010 (Brazil); Graner 2013 (Vietnam); Heberlein 2016 (USA); Kabakian‐Khasholian 2000 (Lebanon); Kraschnewski 2014 (USA); Lagan 2011 (5 HICs: Aus, Can, UK, NZ, USA); Maputle 2013 (South Africa); McNeil 2012 (Canada); Novick 2011 (USA); Spindola 2012 (Brazil); Sword 2012 (Canada); Umeora 2008 (Nigeria); Worley 2004 (New Zealand). ^k^Chimezie 2013 (Nigeria); Ganle 2014 (Ghana); Leal 2018 (Brazil); Mathole 2005 (Zimbabwe); Miteniece 2018 (Georgia); Mugo 2018 (South Sudan). ^l^Andrew 2014 (PNG); Baffour‐Awuah 2015 (Ghana); Ganle 2014 (Ghana); Gheibizadeh 2016 (Iran); Franngard 2006 (Uganda); Larsen 2004 (PNG); Novick 2013 (USA); Sword 2012 (Canada). ^m^Alderson 2004 (UK); Andrew 2014 (PNG); Baffour‐Awuah 2015 (Ghana); Franngard 2006 (Uganda); Heaman 2015 (Canada); Hunter 2017 (Ireland); Larsen 2004 (PNG); Leal 2018 (Brazil); Mathole 2005 (Zimbabwe); McDonald 2014 (Canada); Miteniece 2018 (Georgia); Saftner 2017 (USA); Wright 2018 (Australia). ^n^Abrahams 2001 (South Africa); Ayiasi 2013 (Uganda); Cardelli 2016 (Brazil); Conrad 2012 (Uganda); Mahiti 2015 (Tanzania); Østergaard 2015 (Burkina Faso); Titaley 2010 (Indonesia). ^o^Abrahams 2001 (South Africa); Armstrong 2005 (Australia); Chapman 2003 (Mozambique); Docherty 2011 (UK);Haddrill 2014 (UK); Maputle 2013 (South Africa); McDonald 2014 (Australia); Sword 2003 (Canada); Sword 2012 (Canada). ^p^Ayiasi 2013 (Uganda); Baffour‐Awuah 2015 (Ghana); Biondi 2018 (Brazil); Conrad 2012 (Uganda); Heaman 2015 (Canada); Gheibizadeh 2016 (Iran); Leal 2018 (Brazil); Mathole 2005 (Zimbabwe). ^q^Ayiasi 2013 (Uganda); Bradley 2012 (Ethiopia); Heaman 2015 (Canada); Hunter 2017 (Ireland); Larsen 2004 (PNG); Mathole 2005 (Zimbabwe); McDonald 2014 (Canada); Sword 2012 (Canada). ^r^Ayiasi 2013 (Uganda); Conrad 2012 (Uganda); Mahiti 2015 (Tanzania); Matsuoka 2010 (Cambodia); Shabila 2014 (Iraq). ^s^Alderson 2004 (UK); Andrew 2014 (PNG); Ayiasi 2013 (Uganda); Baffour‐Awuah 2015 (Ghana); Bradley 2012 (Ethiopia); Chimezie 2013 (Nigeria); Franngard 2006 (Uganda); Ganle 2014 (Ghana); Graner 2010 (Vietnam);Gross 2011 (Tanzania); Larsen 2004 (PNG); Manithip 2013 (Laos); Mathole 2005 (Zimbabwe); Miteniece 2018 (Georgia); Molina 2011 (Colombia); Novick 2013 (USA); Rahmani 2013 (Afghanistan); Titaley 2010 (Indonesia). ^t^Bradley 2012 (Ethiopia); Chimezie 2013 (Nigeria); Franngard 2006 (Uganda); Ganle 2014 (Ghana); Graner 2010 (Vietnam); Gross 2011 (Tanzania);Heaman 2015 (Canada); Larsen 2004 (PNG); Manithip 2013 (Laos); Mathole 2005 (Zimbabwe); Mayca 2009 (Peru); Mrisho 2009 (Tanzania); Mugo 2018(South Sudan). ^u^Baffour‐Awuah 2015 (Ghana); Biondi 2018 (Brazil); Chimezie 2013 (Nigeria); Franngard 2006 (Uganda); Graner 2010 (Vietnam); Heaman 2015 (Canada); Larsen 2004 (PNG); Manithip 2013 (Laos); Mathole 2005 (Zimbabwe); Mrisho 2009 (Tanzania); Mugo 2018 (South Sudan). ^v^Ayiasi 2013 (Uganda); Baffour‐Awuah 2015 (Ghana); Chimezie 2013 (Nigeria); Ganle 2014 (Ghana); Graner 2010 (Vietnam); Heaman 2015 (Canada); Hunter 2017 (Ireland); Leal 2018 (Brazil); Manithip 2013 (Laos); Mayca 2009 (Peru); Miteniece 2018 (Georgia); Molina 2011 (Colombia). ^w^Bradley 2012 (Ethiopia); Chimezie 2013 (Nigeria); Franngard 2006 (Uganda); Novick 2013 (USA). ^x^Alderson 2004 (UK); Ayiasi 2013 (Uganda); Conrad 2012 (Uganda); Gross 2011 (Tanzania); Heaman 2015 (Canada); Hunter 2017 (Ireland); Leal 2018 (Brazil); Mathole 2005 (Zimbabwe); Saftner 2017 (USA); Wright 2018 (Australia).

**Summary of findings 3:** Summary of qualitative findings ‐ What matters to women and staff (personalised supportive care)

**WHAT MATTERS TO WOMEN and STAFF** a. Personalised supportive care				
**Summary of review finding**		**Studies contributing to the review finding**	**CERQual assessment of confidence in the evidence**	**Explanation of CERQual assessment**
**Social and community support**				
Women	**W19. Involvement of the community** In settings where women were involved in the organisation and running of ANC services there was wider acceptance of the benefits of ANC and a greater willingness to attend	3 studies^a^	Low confidence	Finding downgraded because of concerns about relevance and coherence. Likely to be a factor in more rural communities
	**W20. Peer support** Women were more likely to access ANC when it was provided in an environment where they felt they were with other pregnant women able to offer emotional, psychological and practical support. This was particularly pertinent in HMICs where the group model of ANC was available but was also evident in LMICs where women were given the opportunity to bond with each other during ANC visits	12 studies^b^	High confidence	Finding also includes data from group ANC programmes
Providers	**P18. Social support for women** Health professionals acknowledged that women appreciated the social support they received from their peers in environments where group ANC was available	7 studies^c^	Low confidence	Finding downgraded because of concerns around coherence and relevance. Finding limited to HICs
**Individualised care**				
Women	**W21. Continuity of care** Women appreciated being seen by the same healthcare professional at each appointment (including pre‐ and postnatal) primarily because this gave them the opportunity to build caring, trusting relationships with healthcare providers	9 studies^d^	Moderate confidence	Finding downgraded because of concerns around coherence and relevance. Limited data from LMICs
	**W22. Woman‐centred care** Women sometimes felt that ANC was provided in an impersonal and non‐individualised manner with an over‐emphasis on physical symptoms and a disproportionate level of attention given to the baby	9 studies^e^	Moderate confidence	Finding downgraded because of concerns around coherence and relevance. Limited data from LMICs
Providers	**P19. Continuity of care** Health professionals offering group ANC felt that the model gave them the opportunity to practise continuity of care and this was seen as a facilitator for the delivery of good‐quality ANC. Where providers were not able to offer continuity of care this was viewed as a barrier to the delivery of quality ANC	10 studies^f^	Moderate confidence	Finding downgraded because of concerns around coherence. Finding limited to HICs
**Attitude of staff**				
Women	**W23. Rude and abusive staff** Women from a variety of different countries and contexts reported rude and hostile behaviour by healthcare providers. As well as a general lack of respect, women reported acts of discrimination and bullying as well as verbal and physical abuse during their ANC visits	15 studies^g^	High confidence	Finding likely to be a factor in a range of settings and contexts
	**W24. Attribution of apathy or laziness** In a few countries women reported that they were too lazy to visit ANC services or felt ambivalent about going. The reasons were not discussed or fully explained by authors	3 studies^h^	Very low confidence	Finding downgraded because of concerns around adequacy of data, methodology and coherence. Appears to be a factor in certain African settings
	**W25. Lack of care in ANC** Brief and cursory encounters with healthcare providers during ANC appointments were highlighted by a number of women in a variety of contexts. The impersonal nature of the ANC encounter, coupled with a reliance on tests and procedures rather than conversation, left women feeling isolated and disenfranchised	8 studies^i^	Moderate confidence	Finding downgraded because of concerns around coherence and relevance. (Read in conjunction with the review finding below)
	**W26. Authentic and kind staff** Women's willingness to engage with ANC was enhanced when healthcare providers were perceived to be authentic and kind. A friendly, respectful and attentive approach was appreciated by women, especially those who were feeling worried or anxious about their pregnancy	18 studies^j^	High confidence	Finding likely to be a factor in a range of settings and contexts
Providers	**P20. Staff attitude** Providers recognised that their attitude and temperament was important even though they sometimes delivered ANC in a hierarchical and didactic manner. They acknowledged that they could be disrespectful to women or become frustrated with women who turned up late or did not heed their advice, and that these behaviours were unlikely to encourage women to engage with ANC. They also associated the qualities of being kind, caring, respectful and calm with the provision of quality ANC	17 studies^k^	High confidence	Finding likely to be a factor in a range of settings and contexts


ANC: antenatal care: HIC: high‐income countries; HMICs: high‐ and ‐middle‐income countries: LIC: low‐income country; LMICs: low‐ and middle‐income countries ^a^Mayca 2009 (Peru); Mumtaz 2007 (Pakistan); Rath 2010 (India). ^b^Armstrong 2005 (Australia); Cabral 2013 (Brazil); Cardelli 2016 (Brazil); Dako‐Gyeke 2013 (Ghana); McDonald 2014 (Canada); McNeil 2012 (Canada); Neves 2013 (Brazil); Novick 2011 (USA); Rath 2010 (India); Sword 2003 (Canada); Teate 2011 (Australia); Umeora 2008 (Nigeria). ^c^Baffour‐Awuah 2015 (Ghana); Heaman 2015 (Canada); Heberlein 2016 (USA); LeMasters 2018 (Romania); McDonald 2014 (Canada); Novick 2013 (USA); Teate 2013 (Australia); ^d^Heberlein 2016 (USA);Larsson 2017 (Sweden); Lasso Toro 2012 (Colombia); McDonald 2014 (Canada); Spindola 2012 (Brazil); Sword 2003 (Canada); Sword 2012 (Canada); Walburg 2014 (France); Worley 2004 (New Zealand). ^e^Armstrong 2005 (Australia); Bessett 2010 (USA); Cabral 2013 (Brazil); Docherty 2011 (UK); Earle 2000 (UK); Heberlein 2016 (USA); Kraschnewski 2014 (USA); Larsson 2017 (Sweden); Walburg 2014 (France). ^f^Alderson 2004 (UK); Baffour‐Awuah 2015 (Ghana); Heaman 2015 (Canada); Hunter 2017 (Ireland);Larsson 2017 (Sweden); McDonald 2014 (Canada); Saftner 2017 (USA); Sword 2012 (Canada); Teate 2013 (Australia); Wilmore 2015a (Australia). ^g^Ayala 2013 (Peru); Choudhury 2011 (Bangladesh); Conrad 2012 (Uganda); Duarte 2012 (Brazil); Gheibizadeh 2016 (Iran); Hunter 2017 (Ireland); LeMasters 2018 (Romania); Maputle 2013 (South Africa); Mayca 2009 (Peru); Munguambe 2016 (Mozambique); Østergaard 2015 (Burkina Faso); Pretorius 2004 (South Africa); Rahmani 2013 (Afghanistan); Shabila 2014 (Iraq); Walburg 2014 (France). ^h^Family Care International 2003 (Kenya); Mrisho 2009 (Tanzania); Myer 2003 (South Africa). ^i^Ayiasi 2013 (Uganda); Bessett 2010 (USA);Cabral 2013 (Brazil); Dako‐Gyeke 2013 (Ghana); Kabakian‐Khasholian 2000 (Lebanon); Mahiti 2015 (Tanzania); Østergaard 2015 (Burkina Faso); Worley 2004 (New Zealand). ^j^Armstrong 2005 (Australia); Cardelli 2016 (Brazil); Docherty 2011 (UK); Duarte 2012 (Brazil); Earle 2000 (UK); Gheibizadeh 2016 (Iran); Heberlein 2016 (USA); Hunter 2017 (Ireland); Kabakian‐Khasholian 2000 (Lebanon); Larsson 2017 (Sweden); Novick 2011 (USA); Pretorius 2004 (South Africa); Shabila 2014 (Iraq); Spindola 2012 (Brazil); Sword 2003 (Canada); Sword 2012 (Canada); Walburg 2014 (France); Worley 2004 (New Zealand). ^k^Andrew 2014 (PNG); Ayiasi 2013 (Uganda); Biondi 2018**(**Brazil); Gheibizadeh 2016 (Iran); Gross 2011 (Tanzania); Heaman 2015 (Canada); Hunter 2017 (Ireland); Leal 2018 (Brazil); LeMasters 2018 (Romania); Manithip 2013 (Laos); Mathole 2005 (Zimbabwe); Miteniece 2018 (Georgia); Rahmani 2013 (Afghanistan); Saftner 2017 (USA); Sword 2012 (Canada); Wilmore 2015 (Australia); Wright 2018 (Australia).

**Summary of findings 4:** Summary of qualitative findings ‐ What matters to women and staff (information and safety)

**WHAT MATTERS TO WOMEN and STAFF** b. Information and safety				
**Summary of review finding**		**Studies contributing to the review finding**	**CERQual assessment of confidence in the evidence**	**Explanation of CERQual assessment**
**ANC as a source of information**				
Women	**W27. ANC as a source of knowledge and information** In many countries women visit ANC providers to acquire knowledge and information about their pregnancy and birth. In situations where this is provided in a useful, appropriate and culturally sensitive manner, sometimes through the use of pictures and stories, it can generate a sense of empowerment and acts as a facilitator to further engagement. In situations where this approach is not adopted, e.g. where tests are not explained properly or information is infused with medical jargon or is outdated and irrelevant, it acts as a barrier and limits further access	25 studies^a^	High confidence	Finding likely to be a factor in a range of settings and contexts
	**W28. Unaware of pregnancy** In some instances women were unaware of the signs and symptoms of pregnancy and accessed ANC services late	3 studies^b^	Very low confidence	Finding downgraded because of concerns around adequacy of data, methodology and coherence
	**W29. Alternative sources of information** When women's informational needs were not met by ANC providers they looked for alternative sources of information. For women in HICs this kind of knowledge was usually acquired through the Internet, whilst women in LMICs tended to turn to friends, relatives or TBAs	9 studies^c^	Moderate confidence	Finding downgraded because of concerns around, relevance and coherence
**ANC as a context for clinical safety**				
Women	**W30. Influence of pregnancy complications** The development of pregnancy‐related problems or complications prompted some women to seek advice and assistance from ANC providers, and for these women acted as an incentive to attend early and regularly in subsequent pregnancies	7 studies^d^	Low confidence	Finding downgraded because of concerns around adequacy of data, methodology and coherence. Limited to LMICs.
	**W31. ANC as a source of medical safety** For women in a variety of different resource settings the availability of medicines, medical tests and screening procedures (e.g. HIV tests and ultrasound) offered safety and reassurance during pregnancy and encouraged ANC attendance	23 studies^e^	High confidence	Finding likely to be a factor in a range of settings and contexts
Providers	**P21. Specific components of/incentives for ANC** Providers believed the availability of iron supplements, the opportunity to offer health promotion information and the opportunity for women to take an active role in tests and screening were all attractive components of ANC. The use of ANC cards to monitor pregnancy progress were not viewed as favourably, as they covered a limited number of the FANC recommendations, meaning women missed out on a number of recommended tests and procedures.	7 studies^f^	Low confidence	Finding downgraded because of concerns around adequacy of data, relevance and coherence


ANC: antenatal care: FANC: focused antenatal care; HIC: high‐income countries; HMICs: high‐ and ‐middle‐income countries: LIC: low‐income country; LMICs: low‐ and middle‐income countries ^a^Abrahams 2001 (South Africa); Ayiasi 2013 (Uganada); Cabral 2013 (Brazil); Cardelli 2016 (Brazil); Conrad 2012 (Uganda); De Castro 2010 (Brazil); Docherty 2011 (UK); Duarte 2012 (Brazil); Graner 2013 (Vietnam); Heberlein 2016 (USA); Kabakian‐Khasholian 2000 (Lebanon); Kraschnewski 2014 (USA); Lasso Toro 2012 (Colombia); Maputle 2013 (South AFrica); McNeil 2012 (Canada); Mrisho 2009 (Tanazania); Mumtaz 2007 (Pakistan); Myer 2003 (South Africa); Neves 2013 (Brazil); Rath 2010 (India); Santos 2010 (Brazil); Shabila 2014 (Iraq); Sword 2003 (Canada); Sword 2012 (Canada); Worley 2004 (New Zealand). ^b^Abrahams 2001 (South Africa); Haddrill 2014 (UK); Myer 2003 (South Africa). ^c^Agus 2012 (Indonesia); Ayiasi 2013 (Uganda); Cardelli 2016 (Brazil); Chowdhury 2003 (Bangladesh); Dako‐Gyeke 2013 (Ghana); Family Care International 2003 (Kenya); Heberlein 2016 (USA); Kraschnewski 2014 (USA); Lagan 2011 (5 HICs: USA, Can, Aus, NZ, UK). ^d^Abrahams 2001 (South Africa); Chapman 2003 (Mozambique); Chowdhury 2003 (Bangladesh); Family Care International 2003 (Kenya); Griffiths 2001 (India); Khoso 2016(Pakistan); Munguambe 2016 (Mozambique). ^e^Agus 2012 (Indonesia); Andrew 2014 (PNG); Ayala 2013 (Peru); Cardelli 2016 (Brazil); Conrad 2012 (Uganda); Dako‐Gyeke 2013 (Ghana); De Castro 2010 (Brazil); Earle 2000 (UK); Family Care International 2003 (Kenya); Graner 2013 (Vietnam); Griffiths 2001 (India); Heberlein 2016 (USA); Hunter 2017 (Ireland); Larsson 2017 (Sweden);Mahiti 2015 (Tanzania); Mrisho 2009 (Tanzania); Munguambe 2016 (Mozambique); Pretorius 2004 (South Africa); Spindola 2012 (Brazil); Stokes 2008 (Uganda); Sword 2012 (Canada); Sychareun 2016 (Laos); Umeora 2008 (Uganda). ^f^Graner 2010 (Vietnam); Gross 2011 (Tanzania); Heaman 2015 (Canada); Hunter 2017 (Ireland); Leal 2018 (Brazil); Saftner 2017 (USA); Sword 2012 (Canada).

## Background

There has been widespread and continuing concern about rates of maternal and neonatal deaths and serious morbidity across the world (UN 2018). Antenatal care (ANC) offers the promise of screening a women and her foetus for actual and potential problems as the pregnancy progresses, and for treating any complications that may arise. Antenatal care is therefore a core component of maternity care provision in most contexts around the world. Quantitative reviews provide information on the efficacy of standard and alternative versions of antenatal care interventions and programmes for women who use them, and for their babies (Catling 2015; Dowswell 2015).

The main measures for the adequacy of ANC provision are the time of the first visit, and the number of antenatal sessions attended (WHO 2002, WHO 2016). Until 2016, World Health Organization (WHO) recommendations for routine antenatal care for women with no existing or historical health problems proposed a four‐session focused antenatal care (FANC) programme during pregnancy, starting before 16 weeks gestation, with specific interventions and activities at each visit (WHO 2002). The number of visits and the content of each visit were based on the WHO Antenatal Care Trial published in 2001 (Villar 2001). However, a Cochrane Review of three cluster‐randomised controlled trials (cluster‐RCTs) (including the original WHO trial), published in 2015, suggested that reduced models of antenatal care might be associated with increased risk of perinatal mortality (Dowswell 2015). This led to a secondary analysis of the results of the original WHO trial, which indicated that in some cases the WHO FANC programme might be associated with higher levels of perinatal mortality. This was particularly evident at 32 to 36 weeks gestation (Vogel 2013). In addition, anecdotal accounts and local audits suggested that the care package was not always delivered with fidelity to the original, tested protocol. Under these conditions, while women may attend for the requisite number of visits, the content or quality of care, or both, may not be appropriate for their needs. Barriers and drivers for good‐quality care provision in general from a staff perspective have indicated a range of factors in intrapartum and postnatal care, including how staff themselves are treated (Munabi‐Babigumira 2017). In 2016, a new WHO ANC guideline was published, recommending eight visits, and taking into account the views and experiences of providers of ANC (WHO 2016). The qualitative analysis undertaken for the 2016 WHO guidelines, and updated to 2019, is the basis for this review.

Although there has been a rise in the percentage of women who attend antenatal care programmes early in pregnancy, and who go on to attend at least three more sessions, these rates are still very low in some countries (Benova 2018; UN 2014). Until recently, it has been assumed that lack of attendance is largely driven by the ‘three delays’ model (Thaddeus 1994). When services are only provided in central locations, and transportation is infrequent, expensive or non‐existent, this is a clear barrier to attendance for some women, especially in cultures where they do not have the autonomy to decide to attend, or to pay for transportation, or both. However, there is increasing evidence that even when services are more accessible and affordable, women do not always use them, especially if they are members of marginalised population groups, such as those living in areas of deprivation, women from ethnic minority groups, refugees, substance misusers, and those from travelling communities (Downe 2009; Finlayson 2013). This observation holds true in both high‐ and low‐ income settings. These studies also note that the biomedical assumptions on which formal ANC is based might not fulfil the needs of all pregnant women, especially in cultures where a more psychosocial approach is culturally normative. The growing recognition of the degree to which women are subject to mistreatment while seeking care in formal maternity care systems also provides an insight into why women may not attend ANC, or why they may attend once and then not again (Bohren 2015; Bowser 2010). This raises questions about why ANC programme vary in quality, and what the drivers or blocks may be for provision of better care in future by staff and healthcare providers.

Qualitative research is the ideal vehicle for answering questions of acceptability, and for exploring the kinds of values and beliefs that might frame provision and uptake of future antenatal care programmes. Data acquired from qualitative studies can inform the content, delivery, and provision of antenatal care, so that it is more effective, acceptable, accessible, and of higher quality for all users, including those who are members of the most marginalised groups. Findings can inform individual studies and reviews of effectiveness, by suggesting outcomes that are relevant to women and providers, as well as by generating hypotheses that can be tested out, for example, in future subgroup analyses. In addition, these methods can inform guidelines by answering questions around the acceptability and feasibility of implementing different aspects of antenatal care, in policy and practice.

This review was designed to complement the existing Cochrane Reviews of the efficacy of different antenatal models of care (Catling 2015; Dowswell 2015), to inform the antenatal care recommendations in the WHO guideline for a positive pregnancy experience (WHO 2016), and to provide insights for the design and implementation of improved antenatal care in the future. It was originally planned as two reviews: one related to service user uptake of ANC, and the other related to service provider provision of good‐quality ANC. However, many studies included both groups, and important insights about complex adaptive interactions between women's and healthcare providers' views and experiences could have been lost if the two groups were treated separately (e.g. in situations where the views of women and healthcare providers were integrated in the analysis within a study). We therefore decided to combine the two reviews, and we report the findings of both reviews together.

### Description of the topic

Antenatal care has been defined as "the routine care that all healthy women can expect to receive during their pregnancy" (NICE 2008)*.* Health promotion activities are also included. Globally, there is wide variation in the number and content of routine antenatal care sessions provided, including a greater or lesser degree of technical monitoring and testing (Dowswell 2015). Generally, the central purpose of ANC is prophylactic, through the monitoring and support of whole populations of pregnant women and of their babies, to maximise the health and well‐being of the majority, and to identify, treat and refer the minority who develop actual or potential complications as the pregnancy progresses.

### How the synthesis might inform or supplement what is already known in this area

This review is focused on access to and uptake of antenatal care. Pawson 1998 has theorised that "programmes are theory incarnate" in social and health care. The mismatch between the theoretical assumptions of routine antenatal care by those who design and deliver it, and those of the cultural context in which it is set, is beginning to be understood as an important barrier to the uptake of antenatal care. Much of what has been termed 'standard' antenatal care is based on an assumption that pregnancy is a fundamentally risky state clinically, and so women need to be regularly assessed for actual or incipient risks. It also assumes that pregnancy is a socially positive condition, that women recognise their pregnancies relatively early, that they have the desire to announce their pregnant state, that they see antenatal care as valuable, and that they have the social, economic and political power to access care when it is provided. In contrast, in many countries pregnancy is seen as a largely healthy physical state, but socially risky. For example, announcing a pregnancy can result in the risk of being subject to the evil eye if jealous neighbours find out (Finlayson 2013). Reluctance to attend clinics among some women may therefore be because they feel there is no need to do so if all is well; or because attending an antenatal clinic reveals the pregnancy, and risks spiritual damage; or because of the extra physical, financial, and social risks of long journeys through difficult terrain. For marginalised women (e.g. those living in areas of deprivation, women from minority ethnic groups, refugees, asylum seekers, substance misusers, women from travelling communities, etc.), reluctance to attend central clinics for antenatal care includes fear of exposure of being pregnant, and consequent social disgrace (for instance, in the case of teenage mothers) (Downe 2009). These insights add to a barriers model in maternity care systems research that has included resource issues (lack of transport options to facilities, lack of funding for transport, need for ‘under‐the‐counter’ payments) and other wider cultural blocks, including the need for women in some societies to ask the permission of elders to travel (Thaddeus 1994). The growing concern over the impact of disrespectful and even abusive attitudes and behaviours by healthcare staff towards pregnant women and their families also suggests a further barrier to accessing care (Bohren 2015; Bohren 2014; Bowser 2010).

Alongside the narratives of pregnant and postnatal women, qualitative data studies are revealing the attitudes, beliefs, and behaviours of maternity service providers. These suggest that in some settings healthcare providers are also exposed to disrespect and abuse (Bowser 2010). This may be vertical or horizontal bullying (Khalil 2009), disrespect, and even the threat of physical or sexual assault as they travel to and from work (Baig 2018). This has been noted in countries in all income brackets.

More prosaically, barriers to the provision of any antenatal care, let alone care of good quality, include lack of essential resources, equipment and drugs (Biza 2015; Ezeonwu 2014). This limits the capacity of healthcare providers to ensure that facilities are attractive and clean, and to provide an adequate response to both routine needs and to emergencies. Beyond this, provision of care in rural locations is limited by understaffing when healthcare providers who might be interested in working in these locations are put off from doing so by a lack of good‐quality housing or schooling for their children (Lehmann 2008).

In high‐income countries, a lack of healthcare providers and limited resources are also cited as factors that influence the provision of quality antenatal care (Royal College of Midwives 2015). Even when there are sufficient resources, there may be an emphasis on the problems caused by the increasingly technical content of care, and especially on the extent to which this hinders positive interpersonal interaction between healthcare providers and pregnant women and their companions (Nyman 2013). These issues can have a negative influence on staff morale and a subsequent impact on the quality of care provided (Smith 2008).

Quantitative reviews of existing programmes provide information on the efficacy of standard biomedical ANC interventions and programmes (Catling 2015; Dowswell 2015). However, they do not explain what women think or feel about them, or if healthcare providers find it easy to offer good‐quality care within these programmes. To date, studies examining the factors that could drive or block ANC uptake or good‐quality provision of services, or both, have not been subject to systematic scrutiny. While it may be assumed that facilitators will simply be the obverse of the barriers, this is not necessarily the case. Many existing ANC programmes that are in theory subject to some of the factors seen as barriers in other settings (such as distance to travel, long waiting times, the need for under‐the‐counter payments) have high attendance figures, and some new models appear to be attractive to both women and healthcare providers in some settings or social groups where uptake is not traditionally high. These include explicitly partnership‐focused models, such as participative women’s groups (Seward 2017), and group‐based Centering Pregnancy (Carlson 2006; Carter 2016; Magriples 2015). It is not clear what underlying mechanisms have catalysed the attractiveness (and in some studies the effectiveness) of either of these existing programmes, or if they also have downsides. For example, there is a suggestion in some reported data that individual women randomised to group‐type antenatal care dislike the consequent lack of privacy, and a study of male partners attending HIV testing with women at their first ANC visit led to a lack of uptake of ANC, presumably due to fear of disclosure of HIV status (Becker 2010). Looking for both promoting as well as inhibiting factors is equally important, but this should not be based on prior assumptions about what is likely to work. The component of this review that seeks to identify uptake of ANC factors is therefore specifically focused on studies that report on the views of pregnant and postnatal women themselves, and not on what other family or community decision‐makers or healthcare providers believe about women’s views. Similarly, the provider component only includes the views of service providers, and not the opinion of others about these views.

The phenomena of interest for this review are therefore the factors influencing the uptake of routine antenatal care from the perspective of pregnant and postnatal women, and those influencing the provision of good‐quality care by healthcare providers**.**

### How the intervention might work

#### Theoretical model

In line with Booth 2015, we assessed a range of theoretical models that could provide a framework for the synthesis of our findings. There is little theoretical research that is directly focused on the mechanisms that underpin healthcare uptake or the quality of health service provision, although there is a wide spectrum of research on components like knowledge of, understanding of, and beliefs about benefits, and about design features, such as the availability, accessibility, appropriateness, and quality components of the AAAQ model (Potts 2008). The underpinning theory for our review is the theory of planned behaviour (Azjen 1991). We chose this by consensus among the review team, as it is widely used in healthcare behavioural research, and it appeared a priori to have a good potential explanatory power for the phenomena in which we were interested. Logic models based on this theory should include input factors relating to attitudes, subjective norms, and behavioural control. Attitudes toward the behaviour in question (in this case, attendance at antenatal clinics) can be expected to predict that behaviour. Subjective norms may be injunctive, i.e. based on what is deemed acceptable behaviour by a particular social group, or descriptive, i.e. the behaviour actually exhibited by the social group. Perceived behavioural control refers to the ability of a person to perform a given behaviour. These input factors are hypothesised to lead to the output of intended behaviour. In the right context, intended behaviours then result in actual behaviours. The theory further states that the input factors are themselves preceded by three psychosocial domains, relating to behavioral, normative, and control beliefs. We hypothesised that the action of attending local antenatal care services is mediated by women's intentions to attend, which are in themselves moderated by their prior attitudes to and beliefs about the value of antenatal care provided locally, by local social norms around such attendance, and by the degree to which they have control over enacting those beliefs and norms, for example, through having the autonomy and finances to travel to where antenatal care is provided. This process in turn is mediated by similar factors operating as mechanisms of effect for staff, creating a complex dynamic system in which both staff and service users are agents. The a priori logic model for the review is given in Figure 1.

**1:**
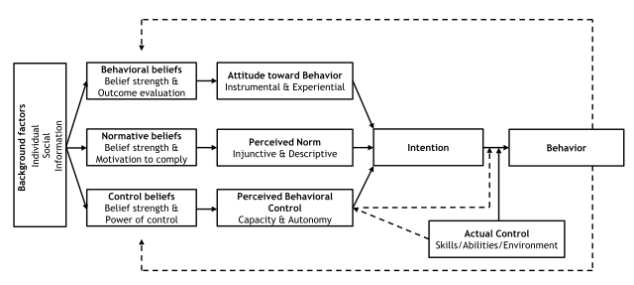
Gjalt‐Jorn Peters. Graphical representation of the reasoned‐action approach. CC BY‐SA 3.0 [https://creativecommons.org/licenses/by‐sa/3.0] https://commons.wikimedia.org/wiki/File:Reasoned_action_approach_text_as_paths.svg

### Why is it important to do this review?

Given the low levels of uptake of ANC in many countries and among some population groups, (e.g. women living in areas of deprivation, women from minority ethnic groups, refugees, asylum seekers, etc.) it is important to determine how ANC can be rendered more acceptable and accessible if it is to fulfil its promise of benefiting women and babies in the future. The World Health Organization has recognised the potential problems with the FANC model, and in some settings the continuing lack of access to ANC as it is currently designed. At the same time, the use of some technologies and techniques, notably ultrasound, is rapidly increasing, with little evidence of added benefit, and some suggestion of possible iatrogenic damage. For example, termination for a female foetus is more likely in some settings when the gender of the baby is identified early (Nie 2011). In other settings, some women are overwhelmed with information, and there is no time for proper discussion or authentically‐informed decision making (Carolan 2007). While ANC has common‐sense value, there is still no strong evidence of impact from RCTs on key maternal and infant outcomes related to uptake of ANC as it is currently delivered around the world. This may be because of the wide variation in content, and the degree to which care is delivered in a way that is acceptable and appropriate for and accessible to the women for whom it is intended. Qualitative review data can provide information on acceptability and accessibility alongside the findings of the current Cochrane Reviews in this area. It can also inform the design of future reviews, to ensure that they capture the elements of ANC that are important to pregnant women.

Healthcare providers play a key role in the implementation and delivery of ANC and are likely to offer valuable insights into their ability to deliver a high‐quality service that is acceptable and accessible to women. The issues that hamper such provision are not just evident at the level of personal beliefs and preferences of providers, but also in the systemic barriers that some face, including resource shortage and workplace bullying. These issues cannot be understood from quantitative effectiveness studies. Qualitative research can offer important insights in this case.

The beneficiaries of this review could therefore be both healthcare providers offering ANC and the women (and their offspring) using it, if policy‐makers, funders of the maternity services and healthcare workers use the findings to design, fund, and provide ANC that is better aligned with women’s needs and expectations, and with provider concerns and values. The review complements existing qualitative and quantitative reviews in this area, as described in Table 5. It allows policy‐makers and those designing and delivering services to better understand what works and what does not, and how what works could be extended into service development and subsequent interventions in the future.

**1:** Published qualitative and quantitative reviews on antenatal care provision and uptake

**Authors, date**	**Title**
**Quantitative reviews**	
Dowswell 2015	Alternative packages of antenatal care for low‐risk pregnant women
Catling 2015	Group versus conventional antenatal care for pregnant women
Mbuagbaw 2015	Health system and community level interventions for improving antenatal care coverage and health outcomes
Till 2015	Impact of offering incentives in exchange for attending prenatal care visits on maternal and neonatal health outcomes
Brown 2015	Giving women their own case notes to carry during pregnancy
Sandall 2016	Midwife‐led continuity models of care compared with other models of care for women during pregnancy, birth and early parenting
**Qualitative reviews**	
Downe 2009	'Weighing up and balancing out': a meta‐synthesis of barriers to antenatal care for marginalised women in high‐income countries
Finlayson 2013	Why do women not use antenatal services in low‐ and middle‐income countries? A meta‐synthesis of qualitative studies
Phillippi 2009	Women's perceptions of access to prenatal care in the United States
Downe 2016a	What matters to women: a systematic scoping review to identify the processes and outcomes of ANC provision that are important to healthy pregnant women

## Objectives

To identify, appraise, and synthesise qualitative studies exploring:

· Women’s views and experiences of attending antenatal care; and factors influencing the uptake of antenatal care arising from women’s accounts;

· Healthcare providers’ views and experiences of providing antenatal care; and factors influencing the provision of antenatal care arising from the accounts of healthcare providers.

## Methods

### Criteria for considering studies for this review

#### Types of studies

This is a systematic review of qualitative primary studies. According to Merriam 2009, "qualitative researchers are interested in understanding the meaning people have constructed, that is, how people make sense of their world and the experiences they have in the world"*.* To achieve this, the review encompassed studies using qualitative designs, such as ethnography and phenomenology. Case studies, grounded theory and mixed methods were all eligible designs, and data collection methods could have been interviews, focus groups, open‐ended survey questions, diaries, and other narrative data collection methods. We did not include studies that collected data using qualitative methods but without performing a qualitative analysis (for example, where qualitative data are only reported using descriptive statistics). We included mixed‐methods studies where it was possible to extract findings derived from qualitative research. We included studies regardless of whether they were carried out alongside studies of effectiveness of antenatal care. We excluded conference abstracts, as they tend to provide inadequate qualitative data and are difficult to formally appraise because of limited information on the methods used to collect, extract and analyse data. We included published PhD theses where no associated and relevant publications were available. We did not include studies scoring lower than C on our chosen quality appraisal tool (Downe 2007; Walsh 2006), because a score of D indicated that they had significant flaws that influenced the trustworthiness of their data (see 'Appraisal of study quality' for more details).

#### Types of participants

In terms of pregnant women, we included studies that reported views about and experiences of routine antenatal care. Pregnant women were eligible, and those who had been pregnant at some time since 1998 (allowing for these accounts to be published by 2000 or subsequently). This time‐frame accounted for changes in antenatal care delivery since the publication of the previous WHO recommendations on antenatal care in 2001 (Villar 2001), which influenced the provision of antenatal care around the world.

We only included studies of healthy women, to ensure compatibility between this review and the content of the WHO antenatal care recommendations that it was primarily designed to inform. Factors influencing uptake of services that are only provided for women/foetuses with particular health or social conditions (such as HIV, malaria, or in‐utero interventions for malformation) are likely to differ from those influencing the behaviours of most pregnant women, who see themselves as healthy. We did not include papers if they only reported what healthcare providers, partners, or families said about the views and experiences of pregnant women.

In terms of healthcare providers, we included studies that reported the views and experiences of staff based in primary, secondary, and tertiary care settings, who were employed by public, private or charity funders to provide routine antenatal care services. Clinical staff fulfilled the WHO 2004 definition of a skilled birth attendant. The accounts of auxiliary and lay health workers were also eligible, if they were paid directly or indirectly (e.g. by paying expenses or through incentive schemes) to provide antenatal care. Healthcare providers who were commenting on their experiences and views of delivering or providing specialist antenatal services for women/babies with specific conditions (such as HIV, malaria, or in‐utero interventions for malformation) were not included, for the same reasons given above for the women. In addition, we did not include papers which only reported on what healthcare providers or managers thought about the views and experiences of women receiving ANC.

#### Setting and care provider

The review includes any setting where ANC was provided, e.g. outpatient/antenatal clinics, or antenatal wards in hospitals, birth centres, local health centres, community centres, children’s centres, or the woman’s home or other local venue. This also includes care provided through e‐ or m‐health platforms. We did not impose any restriction on the healthcare provider in the study selection. Care could have been provided by a range of people, including midwives, nurses, healthcare workers, lay healthcare workers (e.g. trained traditional birth assistants or matrones), obstetricians/gynaecologists, general physicians, and/or peer supporters.

#### Types of interventions

We included studies about healthcare providers' views on routine antenatal care provision, and about women’s views and experiences of using this care or not. We defined routine antenatal care as the contacts, tests, treatments, health promotion activities, information and supportive measures that all women could access during their pregnancy, and that was not designed for women with particular clinical or social conditions or morbidities. This definition was refined from the broader criteria given in the published protocol.

We included studies exploring women’s views and experiences of any or all of the following components of antenatal care, or in the case of the providers of barriers and facilitators to provision of good‐quality care in any of these areas.

- Content of care: consultations, tests, treatments, information, education, advice, support related to maintaining and monitoring a healthy pregnancy, and helping women to prepare for birth and parenting, where these are provided as part of formal antenatal care provision, either publicly or privately funded, for women/foetus without complications.
- How care is provided*:* including the perceived attitudes and behaviours of healthcare providers, and biomedical, psychosocial, relational, and other approaches to care provision.

The review does not include the following.

- Antenatal care programmes/interventions designed for women and babies with specific complications.
- Programmes/interventions that were only about antenatal education, for childbirth or for parenting, or both. These programmes do not include clinical care, tests, and treatments, and they are not usually provided routinely to whole populations of women.

#### Phenomena of interest

The phenomena of interest were the factors that influence the uptake of routine antenatal services from the perspective of pregnant and postnatal women, and the factors influencing the delivery of routine antenatal care, based on the views and experiences of healthcare providers

### Search methods for the identification of studies

#### Electronic searches

We searched PDQ‐Evidence (pdq‐evidence.org) for related reviews in order to identify eligible studies for inclusion, as well as the following electronic databases.

- MEDLINE ‐ Ovid MEDLINE(R) Epub Ahead of Print, In‐Process & Other Non‐Indexed Citations and Daily 1946 to present
- Embase ‐ OvidSP 1974 to present
- CINAHL Complete ‐ EbscoHost
- PsycINFO ‐ EbscoHost
- AMED ‐ EbscoHost
- LILACS ‐ Virtual Health Library
- AJOL (African Journals Online)

We chose these databases as we anticipated that they would provide the highest yield of results based on preliminary, exploratory searches.

Using guidelines developed by the Cochrane Qualitative and Implementation Methods Group for searching for qualitative evidence (Booth 2011), we developed search strategies for each database.We did not impose any language or geographic limit on the searches, but to capture views and experiences of women and healthcare providers since the introduction of focused antenatal care (FANC) programmes, we limited our strategies to publication year 2000 and onwards.

We searched for the studies of women's views and experiences initially between 4th and 9th September 2014, and then updated these on 11th and 12th February 2019. Searches for the providers studies were conducted initially on 4th and 5th February 2015, updated on 11th and 12th February 2019.

We did not include conference abstracts as they tend to provide inadequate qualitative data and are difficult to formally appraise because of limited information on the methods used to collect, extract and analyse data. We included published PhD theses where no associated and relevant publications were available.

Search strategies for all databases are given in Appendix 1 .

#### Searching other resources

We handsearched the reference lists and key authors in the reference lists and undertook backchaining for any references not identified in the search that may be relevant.

We checked the contents pages of over 50 relevant journals as they were issued through Zetoc alerts, over the period the review was undertaken.

### Selection of studies

We collated records into two databases (one for the views and experiences of women, and one for service providers) and removed duplicates. One review author (KF) assessed each study to determine inclusion against the inclusion criteria, and a second author (SD) independently assessed each paper where the fit with the inclusion criteria was unclear. The final decision was made by consensus between SD and KF. If we had needed it, a third author (OT) was available to adjudicate. Where necessary, we contacted study authors for further information.

#### Language translation

For papers that were not published in a language that could be understood by the review authors (i.e. other than English, French, Spanish, Portuguese, Turkish), the abstract was subject to initial translation through open‐source software (Google Translate). For studies that met the inclusion criteria following this process, or if the electronic translation was inadequate to make a decision, we planned to ask members of the multilingual networks associated with the research teams of the review to translate the full text. If this was not possible (for example, for languages outside the scope of the team or any associated staff) we planned to list the study as ‘inclusion not yet confirmed’, to ensure transparency in the review process.

Conceptual translation between languages and cultures is recognised to be an issue in both qualitative and quantitative research (Clark 2017; Al‐Amer 2015; Stevelink 2013). Regmi 2010 discusses the issues of translation (a direct and literal word‐for‐word process) and transliteration (a process of translating meaning which may not be word‐for‐word) in undertaking qualitative research in different language and cultural groups. They use the term 'elegant free translation', from Birbili 2000 which is an approach that in Birbili's analysis can help the reader to 'know what is going on' even if it is less faithful to the original text. Regmi 2010 sees this as "a process involving transcription of only the key themes or few quotes, putting in the context". They recognise that this risks the loss of some precision and meaning, but that it is a pragmatic solution to the complexity and resource demands of full translation in primary qualitative research.

Given that the current review did not aim to be philosophically phenomenological, and that the data we were using (published in English or any other language) was at the level of author themes, selected quotes, and author interpretations of their primary data, we took the pragmatic decision to use the 'elegant free translation' approach to the transliteration of our included studies, rather than translating them word‐for‐word. We applied this approach both at the stage of decisions about inclusion, and for data extraction and analysis.

#### Sampling of studies

Large numbers of studies can threaten the quality of the analysis in qualitative evidence syntheses. Syntheses of qualitative studies aim for greater variation in concepts as opposed to an exhaustive sample that aims to avoid bias. Once we had identified all studies that were eligible for inclusion, we assessed whether maximum variation sampling might be necessary to limit data redundancy, while ensuring optimal data richness and diversity. Key areas of variation that we planned to consider for the service users included the type of antenatal care provision, and the geographical setting. For the healthcare providers, the cadre of the provider was a potential factor. If sampling was required, we planned to create a sampling frame, and to map all eligible studies onto the frame, before reviewing the number of studies in each frame to reach a decision about how many studies in each cell we would include in the review.

#### Data extraction

We recorded study characteristics using an Excel file with multiple worksheets designed specifically for each of the two participant groups included in this review. The study characteristics form recorded details of first study author, date of publication, country of study, context (urban/rural), participant group (parity for the women’s views, type of caregiver for the provider review), type of antenatal care received (level of facility where available), theoretical/conceptual perspective of the study, research methods, sample size, method of analysis, and key themes, as recorded by the study authors in each case.

### Assessing the methodological limitations of included studies

#### Appraisal of study quality

Our inclusion criteria specified that, to be included, a study must have used qualitative methods for both data collection and data analysis. This criterion constituted a basic quality threshold, as we excluded studies that did not meet this standard. In addition, to assess the methodological quality of included studies, two review authors (SD, KF) independently assessed each study for quality, and made the final decision by consensus between SD and KF. We used the criteria from Walsh 2006 which include the study scope and purpose, design, sampling strategy, analysis, interpretation, researcher reflexivity, ethical dimensions, relevance, and transferability. We then applied the A to D grading of Downe 2007, based on Lincoln 1985, as follows.

- A: No, or few flaws. The study credibility, transferability, dependability, and confirmability are high.
- B: Some flaws, unlikely to affect the credibility, transferability, dependability, and confirmability of the study.
- C: Some flaws that may affect the credibility, transferability, dependability, and confirmability of the study.
- D: Significant flaws that are very likely to affect the credibility, transferability, dependability, and confirmability of the study.

We listed but did not include in the central analysis studies that were graded less than C after this process. As can be seen from the summary criteria given above, grading a study as D in our taxonomy means that we judge it to have *'*significant flaws which are very likely to affect the credibility, transferability, dependability, and/or confirmability of the study*'.* We acknowledge that some qualitative researchers believe that all qualitative data have potential value in understanding the phenomenon of interest, but we have argued consistently that including poor‐quality studies in systematic reviews risks a misunderstanding of the final phenomenon, which has potentially important consequences if the findings are to be used in a practice or policy context (Walsh 2006).

#### Data management, analysis, and synthesis

A flowchart illustrating the stages of the analytic process is shown in Figure 2.

**2:**
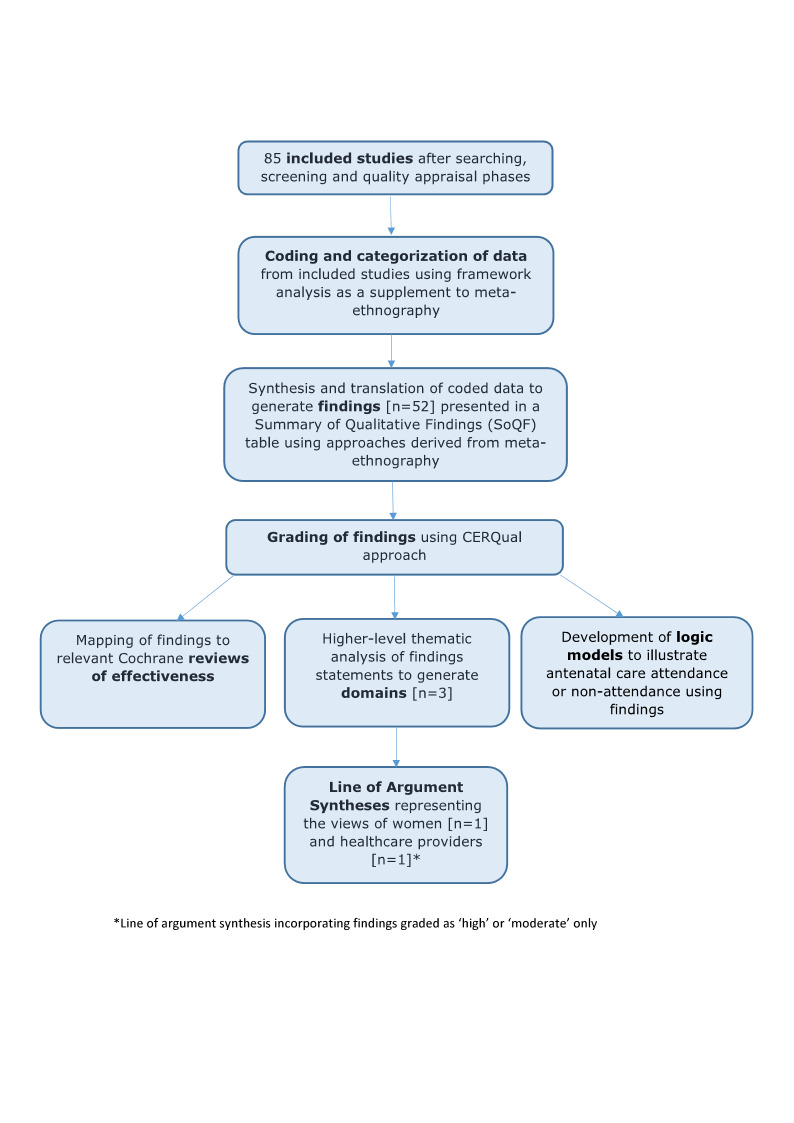
Flow Diagram to Illustrate Analytic Phases

Following the principles of meta‐ethnography (Noblit 1988), we undertook data extraction and analysis simultaneously for each included study in turn. Meta‐ethnography uses an approach based on the grounded theory method of constant comparative analysis, where the analysis is built up study by study. The process requires the researcher to be open to the emergence of new themes, and to ensure that unexpected phenomena can be captured and examined, by subjecting the initial assumptions about what is in the data to both confirmation ('reciprocal analysis') and disconfirmation ('refutational analysis') against each study in turn. This ensures that the product of the review is continually refined as each study is included. However, as this was not a primary grounded theory study, but a qualitative evidence synthesis (QES) (Booth 2016), we did not start from a position of no knowledge. We were explicitly looking for factors influencing both uptake of ANC by women and provision of good‐quality care by staff. We also had some prior beliefs about behavioural change theories. We therefore used framework analysis (Gale 2013) as a supplement to meta‐ethnography. We used the findings to test the explanatory power of our original theoretically‐informed logic model of the theory of planned behaviour (Azjen 1991), given in Figure 1 (the ‘framework’), and where necessary to amend it.

Starting with the earliest published paper, we read each included study in detail, and extracted the relevant verbatim text, along with the codes/themes/theories/metaphors used by the study authors, initially marking them as likely barriers, facilitators, or potentially both barriers and facilitators. We mapped the data from each subsequent paper against this coding structure. Where new data from subsequent papers could not be explained by this emerging taxonomy, we added new categories. Over time, conceptual similarities between some codes in the framework became evident, and these were merged. This resulted in the generation of findings that explained the data at a descriptive level and that were presented in a 'Summary of qualitative findings' table (SoQF), along with the relevant CERQual gradings (see below for details of this process) .

We then undertook a higher‐level thematic analysis, to generate transferable explanatory thematic domains that could be predictive of uptake of ANC. These were translated into two lines of argument syntheses: one to explain the service user data, and one to explain the healthcare provider data. This allowed for theoretical explanations of what might underpin perceived factors influencing women's intended and actual use of local antenatal care, or providers' capacity to provide good‐quality care, in terms of social, behavioural, and control beliefs, and the contextual factors that interact with these factors to prevent or enable an intention for care uptake or quality care provision.

We then tested the explanatory power of the findings in three logic models (full uptake of routine ANC; partial uptake of routine ANC; no uptake of routine ANC), built on our original hypothesis that the theory of planned behaviour would be a good theoretical model for use or non‐use of ANC. The logic models incorporated the key elements of the theory of planned behaviour, namely: 'What do people believe in this context (behavioural beliefs)?'; 'What is normal in this context' (normative beliefs)? and 'How much control do I have over what happens here' (control beliefs)?; the attitudes and perceptions predicted by these beliefs; the intended behaviour that could result; and the actual experiences, all linked to a feedback loop (see figures 4 to 6).

All authors contributed to the final findings, domain structure, lines of argument, and development of the logical models. We made final decisions by consensus, throughout the extraction and analysis process.

##### Assessing confidence in the review findings

We used Confidence in the Evidence from Reviews of Qualitative research (CERQual) to assess the confidence that may be placed in review findings (Lewin 2015). This approach has been developed by the GRADE‐CERQual Project Group 2004. It uses the following four concepts to assess confidence.

- Methodological limitations of included studies: the extent to which there are problems in the design or conduct of the primary studies that contributed evidence to a review finding.
- Relevance of the included studies to the review question: the extent to which the body of evidence from the primary studies supporting a review finding is applicable to the context (perspective or population, phenomenon of interest, setting) specified in the review question.
- Coherence of the review finding: the extent to which the review finding is well grounded in data from the contributing primary studies and provides a convincing explanation for the patterns found in these data.
- Adequacy of the data contributing to a review finding: an overall determination of the degree of richness and quantity of data supporting a review finding.

The above assessments resulted in an overall judgement of confidence in each individual finding as either high, moderate, low, or very low. We list each finding alongside the accompanying CERQual rating in a table that was ordered by the three thematic groups.

#### Planned sub‐analysis

We planned two broad areas of sub‐analyses as follows.

- Data from low‐/middle‐income countries, and those from high‐income countries.

We proposed this sub‐analysis due to differences in uptake, health beliefs, and health system accessibility and quality between these two types of settings.

- Type of respondent: pregnant women; postnatal women; those who have and who have not used antenatal care; type of healthcare provider.

We planned this sub‐analysis because expectation and experience may result in different accounts. Women who have not attended antenatal care may have a different experience of influencing factors than those who have used antenatal care. Healthcare providers from some cadres, such as medical practitioners working in central facilities in high‐income countries, may have very different views and experiences of phenomena that might influence care quality (such as, for example, stock‐outs) than midwives operating in very rural low‐resource contexts.

We also considered that, depending on what emerged from the data, we might have considered other sub‐analyses, including the type of antenatal care that the views and experiences relate to (for example, FANC; classic schemes with more than four routine visits; partnership‐based models), and care setting/location of antenatal care provision.

In the event, our data did not suggest that formal sub‐analyses along any specific lines would enhance the explanatory power of our findings. Instead, where findings might have particular resonance for particular groups/contexts, based on the data, we have noted this in the narrative account of our findings. Findings in which we had high or moderate confidence based on GRADE‐Cerqual appraisal are particularly likely to have high explanatory power across all groups.

##### 'Summary of qualitative findings' table(s) and evidence profile(s)

We present summaries of the findings and our assessments of confidence in these findings in a series of 'Summary of qualitative findings' tables. We present detailed descriptions of our confidence assessment in evidence profiles.

##### Linking the findings to relevant Cochrane Intervention Reviews and WHO guidelines

We identified existing quantitative Cochrane Reviews of interventions containing at least one reference to antenatal care provision in the title (Table 5). We examined identified reviews to see if the authors paid attention to possible underlying theories or mechanisms of effect that might influence the effectiveness of the interventions they were examining. Where authors identified any relevant theories or mechanisms, we mapped them to the findings identified in this review (Table 6).

**2:** Programme theory and/or intervention design factors reported in current effectiveness reviews of models of ANC provision, and related findings

**Author/date**	**Review title**	**Programme theory/Intervention design factors related to findings in current review**	**Related Findings**
Dowswell 2015	Alternative packages of antenatal care for low‐risk pregnant women	ANC is a series of visits with clinical interventions: the main hypothesised mechanism of effect was the number of visits	W31 P21
Catling 2015	Group versus conventional antenatal care for pregnant women	Self‐care; continuity of co‐ordinator of group; time to socialise; flexible content around a standard core; facilitative approach; increased time in antenatal care; education; social and peer support	W15, 17, 20, 21, 22, 27 P11,18,19
Mbuagbaw 2015	Health system and community‐level interventions for improving antenatal care coverage and health outcomes	Staff capacity building; increasing numbers of midwives; reduction/payment of user fees and transport costs; adopting private sector model if superior to alternatives; individual sessions; community education and information to encourage attendance); engaging multiple stakeholders. Some interventions based on behaviour change theories	W1, 2, 6, 10, 12, 17, 19, 27 P1, 2, 4, 5, 9, 11, 12, 14, 15
Till 2015	Impact of offering incentives in exchange for attending prenatal care visits on maternal and neonatal health outcomes	Providing extra finances or resources if women attend ANC is sufficient incentive for them to do so	W6, 12, 18 P5
Brown 2015	Giving women their own case notes to carry during pregnancy	Transfer of information when women move from one facility to another. Easy access to notes (for professionals); reduce the storage and administrative costs; improved information for the woman and improved communication between the woman and the caregiver	W27
Sandall 2016	Midwife‐led continuity models of care compared with other models of care for women during pregnancy, birth and early parenting	Women’s health needs are not isolated events: longitudinal relationship between women and providers; perception of being ‘known’ and cared for by the provider; ‘co‐ordinated and smooth progression of care from the patient’s point of view’; woman‐centeredness: improved management (communication across and between women, professionals, and agencies); information (timely availability of relevant information); and relationship (therapeutic relationship over time.	W21, 22, 27 P19

We also used the findings as the primary data for informing panel judgements on the acceptability and value of proposed components and interventions for the 2016 WHO ANC guidelines (WHO 2016).

### Review author reflexivity

In keeping with quality standards for rigour in qualitative research, the review authors considered their own views and opinions on antenatal care as possible influences on the decisions made in the design and conduct of the study, and in turn on how the emerging results of the study influenced those views and opinions. All review authors believed at the outset that contact with formal and informal caregivers throughout pregnancy was valuable, but that formal antenatal care provision is generally over‐focused on clinical procedures and the assessment of risk/ill health, with too little focus on psychosocial aspects of pregnancy. We therefore used refutational analytic techniques ('disconfirming analyses') to minimise the risk that these presuppositions would skew the analysis and the interpretation of the findings.

## Results

### Results of the search

In total, our searches generated 21,136 hits, including 13,022 from the original searches and 8114 from the updated searches conducted in February 2019. After screening by title and abstract, we retrieved 522 full‐text articles and after further review excluded 376 because they failed to meet our inclusion criteria. Of the remaining 146 we ruled out a further six because they failed to meet our quality appraisal checks (Lohmann 2018; Murira 2003; Nigenda 2003; Påfs 2015; Pell 2013; Tsawe 2014) and we excluded a single Japanese study (Aikawa 2004) because we were unable to translate it (listed under Studies awaiting classification). This left 139 studies, i.e. 65 from our original searches and 74 from our updated searches. Because of the large number of studies we decided to include all 65 from the original searches and a sample from the updated searches. Our sampling strategy for the studies located in the updated searches was based on the following rationale:

- Include all of the eligible healthcare provider studies, as there were only 10 in the original searches;
- Include all of the eligible studies conducted in a European (non‐UK) or Middle Eastern setting, as these areas were under‐represented in the original searches;
- Include a random sample from the remaining studies to reflect an overall sample size of about 25% of the studies eligible in the updated searches.

Based on these criteria, we included seven additional healthcare provider‐only studies, five European studies, one study from Iran, a further four studies representing women's views of ANC and three mixed‐population studies (detailing the accounts of women and healthcare providers). We added these additional 20 studies to the 65 original studies to give a total of 85 included studies for the final analysis

See Figure 3 for a PRISMA diagram illustrating this process.

**3:**
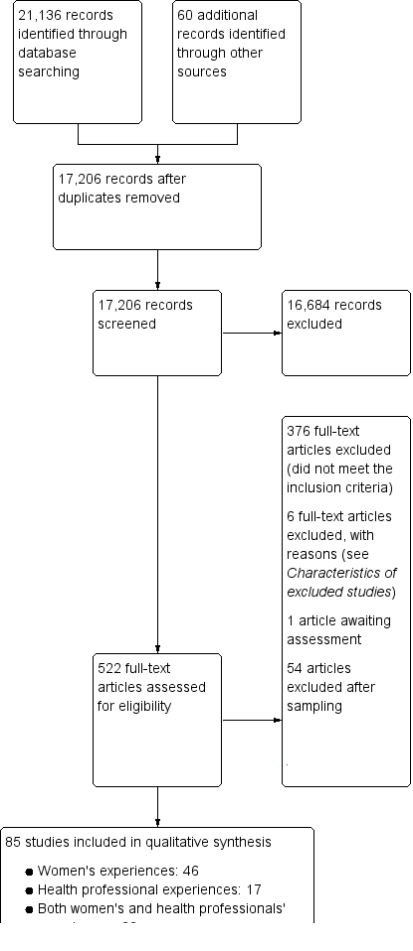
PRISMA flow diagram.

#### Description of the studies

The papers reporting on women’s experiences included antenatal and postnatal women of all parities from 37 countries, living in rural, urban and semi‐urban settings, and with varying levels of uptake of ANC, including no uptake. The date range for these studies was 2000 to 2018, and most of the studies were quality‐graded as having 'few' or 'some' flaws.

The papers reporting on providers' experiences included midwives, nurses, doctors, traditional birth attendants (TBAs), and health service managers from 26 countries, working in rural, urban and semi‐urban settings. The date range for these studies was 2004 to 2018, and most of the studies were quality‐graded as having 'few' or 'some' flaws.

The characteristics and quality assessments of the 85 included studies are shown in Table 7

**3:** Quality Appraisal

**Paper**	**Participants**	**Details of data ** **collection**	**Details of ** **analysis**	**Depth, detail, richness**	**Quality rating**
**Abrahams 2001**	Women	Yes ‐ Adequate	No ‐ No details	Yes ‐ Adequate	C+
**Agus 2012**	Women	Yes ‐ Good	Yes ‐ Limited	Yes ‐ Good ‐ within the context of their traditional beliefs	B
**Alderson 2004**	Health professionals	Yes ‐ Limited	No ‐ Poorly described	Yes ‐ Very good (within the context of ethics)	B
**Andrew 2014**	Women	Yes ‐ Good	Yes ‐ Good	Yes ‐ Very Good	A
	Health professionals	Yes ‐ Limited	Yes ‐ Good	Ok ‐ limited provider quotes	B+
**Armstrong 2005**	Women	Yes ‐ Good	Yes ‐ Good	Yes ‐ Very Good	B+
**Ayala 2013**	Women	Yes ‐ Good	Yes ‐ Good	Yes ‐ Very Good	A−
**Ayiasi 2013**	Women	Yes ‐ Good	Yes ‐ Good	Yes ‐ adequate ‐ but few quotes related to ANC specifically	B
	Health professionals	Yes ‐ Good	Yes ‐ Good	Yes ‐ Adequate	B
**Baffour‐Awuah 2015**	Health Professionals	Yes ‐ Good	Yes ‐ Good	Yes ‐ Good (although largely descriptive)	B+
**Bessett 2010**	Women	Yes ‐ Good	Yes ‐ Adequate	Yes ‐ Very Good	B−
**Biondi 2018**	Health Professionals	Yes ‐ Adequate	Yes ‐ Adequate	Yes ‐ Good ‐ although highly contextual	C+
**Bradley 2012**	Health professionals	Yes ‐ Good	Yes ‐ Good	Yes ‐ Good (focused around a government initiative to increase ANC uptake in rural area's)	A
**Cabral 2013**	Women	Yes ‐ Adequate	No ‐ Very limited	Yes ‐ Adequate	C+
**Cardelli 2016**	Women	Yes ‐ Adequate	Yes ‐ Limited	Yes ‐ Adequate	C+
**Chapman 2003**	Women	Yes ‐ Good	Yes ‐ Good	Yes ‐ Very good	A
**Chimezie 2013**	Women and Health professionals	Yes ‐ Very Good	Yes ‐ Very Good	Yes ‐ Very Good	A
**Choudhury 2011**	Women	Yes ‐ Good	Yes ‐ Limited	Yes ‐ Adequate	B‐
**Chowdhury 2003**	Women	Yes ‐ Good	Yes ‐ Adequate	Yes ‐ Adequate	B−
**Conrad 2012**	Women	Yes ‐ Good	Yes ‐ Good	Yes ‐ Very good	A−
	Health professionals	Yes ‐ Adequate	Yes ‐ Good	Ok ‐ very limited provider views	C+
**Coverston 2004**	Women	Yes ‐ Good	Yes ‐ Adequate	Yes ‐ Good	B
**Dako‐Gyeke 2013**	Women	Yes ‐ Good	Yes ‐ Adequate	Yes ‐ Good	B+
	Health professionals	Yes ‐ Good	Yes ‐ Adequate	Ok ‐ mainly women's views	C+
**De Castro 2010**	Women	Yes ‐ Limited	Yes ‐ Good	Yes ‐ Adequate ‐ very descriptive and researcher led	B
**Docherty 2011**	Women	Yes ‐ Good	Yes ‐ Good	Yes ‐ Adequate	B
**Duarte 2012**	Women	Yes ‐ Limited	Yes ‐ Limited	Yes ‐ Adequate	C+
**Earle 2000**	Women	Yes ‐ Good	Yes ‐ Good	Yes ‐ Good	B+
**Family Care International 2003**	Women	Yes ‐ Good	Yes ‐ Good	Yes ‐ Good ‐ but part of a report with multiple respondents with different community roles	B+
**Franngard 2006**	Health professionals	Yes ‐ Good	Yes ‐ Good	Yes ‐ Good	B+
**Ganle 2014**	Women and Health professionals	Yes ‐ Good	Yes ‐ Very Good	Yes ‐ Good	A−
**Gheibizadeh 2016**	Women and Health professionals	Yes ‐ Good	Yes ‐ Good	Yes ‐ Good	A−
**Graner 2010**	Health professionals	Yes ‐ Good	Yes ‐ Good	Yes ‐ Good	B+
**Graner 2013**	Women	Yes ‐ Good	Yes ‐ Adequate	Yes ‐ Good	B
**Griffiths 2001**	Women	Yes ‐ Good	Yes ‐ Adequate	Yes ‐ Good	B
**Gross 2011**	Health professionals	Yes ‐ Good	Yes ‐ Good	Yes ‐ Good	B
**Haddrill 2014**	Women	Yes ‐ Good	Yes ‐ Adequate	Yes ‐ Good ‐ focused on women who booked late	A−
**Heaman 2015**	Health professionals	Yes ‐ Good	Yes ‐ Good	Yes ‐ Good	B
**Heberlein 2016**	Women	Yes ‐ Good	Yes ‐ Good	Yes ‐ Good	A−
**Hunter 2017**	Women and Health professionals	Yes ‐ Good	Yes ‐ Good	Yes ‐ Very Good	A
**Kabakian‐Khasholian 2000**	Women	Yes ‐ Adequate	Yes ‐ Limited	Yes ‐ Good	B−
**Khoso 2016**	Women	Yes ‐ Adequate	No ‐ Limited	Ok ‐ inadequate detail given nature of phenomenological approach	C
**Kraschnewski 2014**	Women	Yes ‐ Good	Yes ‐ Adequate	Yes ‐ Good ‐ focused on the use of smart phones for antenatal information	B
**Lagan 2011**	Women	Yes ‐ Good	Yes ‐ Good	Yes ‐ Good ‐ focused on use of the Internet for antenatal information	A−
**Larsen 2004**	Women and Health professionals	Yes ‐ Adequate	No ‐ Poorly described	Ok ‐ limited in terms of provider quotes	C+
**Larsson 2017**	Women	Yes ‐ Good	Yes ‐ Good	Yes ‐ Very Good	A
**Lasso Toro 2012**	Women and Health professionals	Yes ‐ Adequate	No ‐ Limited	Poor ‐ lost in translation	C+
**Leal 2018**	Health professionals	Yes ‐ Limited	No ‐ Limited	Ok ‐ largely descriptive and lacking insight	C
**LeMasters 2018**	Women and Health professionals	Yes ‐ Good	Yes ‐ Adequate	Yes ‐ Good ‐ incorporating perspectives from a wide variety of relevant stakeholders	B
**Mahiti 2015**	Women	Yes ‐ Good	Yes ‐ Very Good	Yes ‐ Good ‐ includes data from a large number of relevant stakeholders	B+
**Manithip 2013**	Health professionals	Yes ‐ Good	Yes ‐ Good	Yes ‐ Good	B+
**Maputle 2013**	Women	Yes ‐ Good	Yes ‐ Adequate	Yes ‐ Very Good	A
**Mathole 2005**	Health professionals	Yes ‐ Good	Yes ‐ Good	Yes ‐ Good	B+
**Matsuoka 2010**	Women	Yes ‐ Limited	No ‐ Very limited	Yes ‐ Good ‐ specific barriers identified and discussed	B−
**Mayca 2009**	Women	Yes ‐ Adequate	Yes ‐ Good	Yes ‐ Good but loses a little in translation	B−
	Health professionals	Yes ‐ Adequate	Yes ‐ Limited	Yes ‐ adequate, loses a little in translation	B−
**McDonald 2014**	Women	Yes ‐ Good	Yes ‐ Adequate	Yes ‐ Good	B−
	Health professionals	Yes ‐ Good	Yes ‐ Good	Yes ‐ Good ‐ within the context of group ANC	B
**McNeil 2012**	Women	Yes ‐ Good	Yes ‐ Good	Yes ‐ Good ‐ within the context of Group ANC	B+
**Miteniece 2018**	Women and Health professionals	Yes ‐ Good	Yes ‐ Very Good	Yes ‐ Very Good ‐ detailed exploration of context and wider implications	A
**Molina 2011**	Health professionals	Yes ‐ Adequate	Yes ‐ Adequate	Yes ‐ Good	B−
**Mrisho 2009**	Women	Yes ‐ Good	Yes ‐ Limited	Yes ‐ Good	B
	Health professionals	Yes ‐ Good	Yes ‐ Adequate	Yes ‐ Good ‐ experiences of ante and post‐natal care	B
**Mugo 2018**	Health professionals	Yes ‐ Adequate	Yes ‐ Good	Yes ‐ Very Good	B
**Mumtaz 2007**	Women	Yes ‐ Good	Yes ‐ Good	Yes ‐ Good detail but limited relevant quotes to support findings	B+
**Munguambe 2016**	Women and Health professionals	Yes ‐ Adequate	Yes ‐ Good	Yes ‐ Adequate ‐ part of a larger study on maternity care with limited ANC data	B
**Myer 2003**	Women	Yes ‐ Limited	Yes ‐ Adequate	Yes ‐ Adequate	C+
**Neves 2013**	Women	Yes ‐ Good	Yes ‐ Adequate	Yes ‐Good	B
**Novick 2011**	Women	Yes ‐ Good	Yes ‐ Good	Yes ‐ Very Good (Group Prenatal Care)	A−
**Novick 2013**	Women and Health professionals	Yes ‐ Good	Yes ‐ Good	Yes ‐ Good, within the context of Group ANC	A−
**Østergaard 2015**	Women and Health professionals	Yes ‐ Good	Yes ‐ Good	Yes ‐ Good	A−
**Pretorius 2004**	Women	Yes ‐ Adequate	Yes ‐ Good	Yes ‐ Adequate ‐ mainly about attitudes towards and experiences of birth	B
**Rahmani 2013**	Women	Yes ‐ Adequate	Yes ‐ Limited	Yes ‐ Adequate	C+
	Health professionals	Yes ‐ Adequate	Yes ‐ Adequate	Yes ‐ Good	B
**Rath 2010**	Women	Yes ‐ within the context of the research design	Yes ‐ Good	Yes ‐ Very good ‐ largely framed around an evaluation of a group antenatal care intervention	A−
**Saftner 2017**	Health professionals	Yes ‐ Good	Yes ‐ Good	Yes ‐ Good ‐ largely in the context of support for physiologic birth	B
**Santos 2010**	Women	Yes ‐ Limited	No ‐ Very limited	Yes ‐ adequate ‐ quotes appear to be from survey data?	C+
**Shabila 2014**	Women	Yes ‐ Good	Yes ‐ Good	Yes ‐ Good	B+
**Simkhada 2010**	Women	Yes ‐ Complicated	Poorly explained	Yes ‐ Adequate ‐ but reservations about validity due to the complicated design	C+
**Spindola 2012**	Women	Yes‐ Limited	Yes ‐ Limited	Yes ‐ Adequate	C+
**Stokes 2008**	Women	Yes ‐ Adequate	Yes ‐ Good	Yes ‐ Adequate	B‐
**Sword 2003**	Women	Yes ‐ Good	Yes ‐ Good	Yes ‐ Very good	A
**Sword 2012**	Women	Yes ‐ Good	Yes ‐ Good	Yes ‐ Good ‐ specifically about the quality of ANC provision	A−
	Health professionals	Yes ‐ Good	Yes ‐ Very good	Yes ‐ Good (mixture of findings from providers and women)	A−
**Sychareun 2016**	Women and Health professionals	Yes ‐ Good	Yes ‐ Limited	Yes ‐ Adequate ‐ focus on traditional pregnancy practices rather than ANC specifically	B−
**Teate 2011**	Women	Yes ‐ [Survey]	No ‐ Very limited	Yes ‐ Adequate	C+
**Teate 2013**	Health professionals	Yes ‐ Good	Yes ‐ Good	Yes ‐ Good, within the context of group ANC	A
**Thwala 2011**	Women	Yes ‐ Good	Yes ‐ Good	Yes ‐ Very Good	A
**Titaley 2010**	Women	Yes ‐ Limited	Yes ‐ Adequate	Yes ‐ Adequate	C+
	Health professionals	Yes ‐ Good	Yes ‐ Adequate	Ok ‐ limited views from providers	C+
**Umeora 2008**	Women	Yes ‐ Adequate	No ‐ Very limited	Yes ‐ Adequate ‐ directly answers research question but poor quality	B−
**Walburg 2014**	Women	Yes ‐ Limited	Yes ‐ Adequate	Yes ‐ Adequate ‐ highly descriptive for a phenomenological study	B−
**Wilmore 2015**	Health professionals	Yes ‐ Good	No ‐ Poorly described	Yes ‐ Good ‐ supported by quotes and snippets of conversations from observational data	B−
**Worley 2004**	Women	Yes ‐ Good	Yes ‐ Adequate	Yes ‐ Adequate ‐ barrier led	B
**Wright 2018**	Health professionals	Yes ‐ Good	Yes ‐ Very good	Yes ‐ Very good	A

The studies took place in 41 countries across five continents, and were conducted in eight high‐income countries, 18 middle‐income countries and 15 low‐income countries. Six studies were translated (five Portuguese and one Spanish) but we were unable to translate one Japanese study (Aikawa 2004). Methods used included grounded theory, phenomenology, narrative analysis of survey data, Q methodology and simple interview or focus group studies. Overall, we include the views of more than 1950 women and more than 780 healthcare providers; some studies did not specify the exact number of participants.

### Methodological limitations of the studies

Of the 85 included studies, we rated 66 in the 'A' or 'B' range after quality appraisal, meaning they had few or some flaws that we considered to be relatively minor and unlikely to affect the reliability of the findings. We graded 19 studies as C or C+, meaning that they had some flaws that might affect the reliability of the findings. Of these studies, the methodological limitations were primarily associated with poor or inadequate reporting of data extraction techniques or the approaches used to analyse data. One study (Teate 2011), graded as C+, adopted a survey design and analysed participants' free‐text responses using simple thematic analysis. Whilst the qualitative findings were relevant to the review, the overall level of depth, detail and richness was relatively poor. We graded two studies as 'C', one (Leal 2018) because the recruitment procedures, data extraction and analysis techniques were unclear, and the other (Khoso 2016) because it purported to use a phenomenological design but lacked the methodological details associated with this approach. Details of the methodological limitations of all of the studies are shown in Table 7.

### Findings of the review

Our primary analysis generated 31 findings relating to women’s experiences and views (17 moderate to high confidence), and 21 relating to maternity care providers (14 moderate to high confidence). Three thematic domains encompassed all of the findings across both groups. These were: Socio‐cultural context; Design and provision; and What matters to women and staff*.* The third domain was sub‐divided into two conceptual areas; personalised supportive care, and information and safety. Table 1, Table 2, Table 3, and Table 4 list all the findings in detail, with their CERQual ratings.

Eleven findings were present for both service users and providers (Table 8). They indicate that both service users and providers were conscious that ANC was provided in a social context, in which the local social norms could operate either to enhance or resist uptake. Resource issues are also noted, as well as the need for well‐organised services that offer safety, appropriate information, and positive interpersonal relationships, notably through continuity of care/carer.

**4:** Findings that emerged from both women and provider data

**Domain**	**CERQual assessment**		
	**High/moderate confidence**	**Mixed confidence**	**Low/very low confidence**
**Sociocultural context**	Influence of others	‐	‐
**Service design and provision**	Indirect cost of services Time spent with the professional/service user Flexibility of appointments	Proximity of the clinic to the local community Need for privacy Lack of resources	Disorganised services
**What matters to women and staff**	Continuity of care	ANC as a source of knowledge and information ANC as a source of clinical safety	‐

ANC: antenatal care

A summary of the findings, organised according to the three domains, is discussed below.

#### Domain one: Socio‐cultural context

The domain of socio‐cultural context was influenced by a number of sub‐domains arising from the findings, including the 'Influence of traditional beliefs', the 'Influence of local beliefs and traditional maternity practices', the notion of 'Pregnancy as a healthy state', the 'Selective use of antenatal care' and 'Gender issues'.

##### Influence of traditional beliefs

For many women in low‐ and middle‐income countries (LMICs), and particularly for those living in rural areas, there were a variety of medical, spiritual and supernatural beliefs that they saw as preferable alternatives to engagement with formal ANC services. In these contexts biomedical approaches to health care were not culturally normative. Women used community resources, including TBAs and shamen, to treat pregnancy‐related conditions and allay concerns about pregnancy outcomes. In some contexts women were precluded from attending antenatal facilities because of supernatural fears relating to pregnancy disclosure. Sometimes these fears were based on religious beliefs, but in most cases the influence of sinister forces, described as evil spirits or 'the evil eye', restricted or delayed ANC engagement, “It is a traditional belief; there are some people that when you tell them and they have evil eyes and mind they can destroy it [the foetus] so unless the pregnancy shows then you tell. If not when it is two or three months you cannot tell” (Dako‐Gyeke 2013, Ghana). In other contexts, faith in the knowledge of traditional or spiritual healers limited ANC engagement, "When some women conceive they always have pain. This kind of pain could provoke a miscarriage. They must find the person who knows how to treat this. A curandeiro [traditional healer] or pastor could treat this, but it depends on the woman. There are some cramps that are from your body, and there are illnesses provoked by mal espirito [evil spirit]. Only a prophet or curandeiro can say which is which. In the hospital they don’t know how to differentiate. But neither the hospital nor the curandeiro can cure without God’s help" (Chapman 2003, Mozambique). Where women felt their traditional beliefs were ignored or disrespected by healthcare providers their inclination to visit formal antenatal services was reduced (Family Care International 2003, Kenya), but in other instances where healthcare providers made cultural understanding of traditional beliefs an engagement priority, women appreciated the efforts and were more likely to engage, "There are doctors who know the plants here, from our area and they make us see that our plants do serve us so we don't have to take only the pure medicine [Western medicine]" (Mayca 2009, Peru).

In many LMICs a woman's decision to engage with antenatal services was influenced by a variety of different people, including family members, community representatives and community health workers. The influence might be positive or negative and could depend on financial considerations, traditional beliefs or familial hierarchies, or both. In some cultures deference to an older female family member (usually the mother, or more often the mother‐in‐law) restricted ANC attendance because of a lack of knowledge of ANC or a belief in perpetuating and protecting traditional practices, *"*My mother‐in‐law said that pregnant women didn't go for antenatal check‐ups in the old days. She told me that she had all her children without any antenatal check‐ups and all are fine. She questioned why different foods and antenatal check‐ups are necessary for pregnant women. That's why I didn't go" (Simkhada 2010, Nepal).

##### Influence of local beliefs and traditional maternity practices

In rural communities of LMICs where providers were able to co‐operate effectively with influential community members or TBAs, use of ANC services was perceived to be better than where such co‐operation was not present. This is starkly illustrated in a study from rural Ethiopia where 'good performing clinics' (with high ANC coverage rates) were compared with contextually similar 'poor performing clinics' (with low ANC coverage rates) (Bradley 2012, Ethiopia). In the 'good performing clinics' the importance of community engagement was identified by the providers as being one of the keys to their success, "There are priests and there are also sheiks. These people are community leaders; therefore we go to them and we tell them that such and such person is not willing to listen to us and we ask them to help us get through to them. After that, they would go to the community with us and they would tell people that what we had taught them was true"; (Bradley 2012, Ethiopia). In the 'poor performing clinics' these kinds of connections were limited or non‐existent. In some rural African communities where tension sometimes existed between the traditional practices adopted by TBAs and the modern approaches used by community midwives, an emphasis on co‐operation rather than confrontation was seen as a way of encouraging women to attend ANC services, "Government should put more effort into TBA's because the community has trust in them. They are living with them, some of them are friends and relatives so we need to be nearer to them" (Franngard 2006, Uganda). In a variety of LMICs the reliance on traditional maternity practices was viewed as a barrier to ANC engagement by local providers. Health professionals acknowledged that women sometimes preferred to be seen by a TBA because of their understanding of community‐derived customs and rituals relating to pregnancy. This mutual understanding generated a sense of trust in traditional practices, especially when biomedical approaches to ANC conflicted with cultural beliefs, “For some of the pregnant women when you talk to them like that and tell them about a complication, if there is any TBA around they rather go to that place, rather than the health facility they have been referred to” (Dako‐Gyeke 2013, Ghana).

##### Pregnancy as a healthy state

Across a wide variety of settings and contexts, including urban and rural locations, women perceived pregnancy to be a healthy state and saw no reason to attend an antenatal clinic unless they felt unwell, “We go to the doctor only if the child is unwell or if the mother has excessive bleeding”. (Khoso 2016, Pakistan). Some women viewed pregnancy as a positive experience and held no particular fears or concerns about potential danger signs or complications. This view is clearly reflected in the following statement from a woman in rural West Java, (Indonesia), “I think pregnancy is a normal process so you do not need to think bad thoughts about it" (Agus 2012, Indonesia). Support for this belief was also evident in urban locations where arguably public health messages about the value of antenatal care were more likely to be received and operationalised, as this quote from a woman in Dakka, Bangladesh implies, "As no one expects to be sick during pregnancy, visiting the centre for a check‐up is not necessary. What is the point for going for a check‐up in a healthy condition" (Chowdhury 2003, Bangladesh). Even in high‐income settings, some women postponed or delayed engagement with ANC services because of a perception of feeling well or because of previous experiences with healthy pregnancies, "I think if there were any previous problems with them [previous pregnancies] I would have probably found out but I just felt healthy, I felt OK you know, I just felt normal basically and I suddenly saw my belly getting a bit bigger and my clothes weren’t fitting as much”. (Haddrill 2014, UK).

##### Selective use of antenatal care

In certain settings women made selective use of ANC services, and in some instances this was simply based on their desire to confirm a pregnancy. Women were aware that a test at the clinic would prove their pregnancy status and, provided the clinic was reasonably accessible, would take advantage of this service. However, this did not necessarily mean that women visited a clinic at the first sign of pregnancy or even within the first three months, "I started going to the clinic when I was 5 months pregnant; I was not sure that I was pregnant and therefore decided to go and confirm it" (Mrisho 2009, Tanzania). Selective use of ANC was also evident in contexts where women saw a value in obtaining a paper record of their ANC visit(s) in the form of an 'ANC Card'. This finding was peculiar to an African context where the card was viewed as an insurance policy or a passport allowing access to a hospital or health facility when the time came to give birth, "If you come to the clinic for an antenatal care card, you are booking yourself a bed in the clinic. . . . How could you deliver in the clinic without a card?" (Myer 2003, South Africa). In these contexts, however, the value was placed on the card rather than on antenatal care per se and some women just went to receive the card without any understanding of the wider benefits of antenatal care, "I am just afraid of being denied services when I need them, so one must just go [to ANC] to get the [clinic] card. If you do not have a card, they will not accept you when there is a problem.... Otherwise, we could just rest at home" (Mrisho 2009, Tanzania).

##### Gender issues

Our findings also highlighted several issues relating to gender which generally restricted women's engagement with ANC. The first of these, relating to women's financial dependence on their husband, was demonstrated in a small number of settings where patriarchal systems were dominant. In these contexts women had to ask for money to visit ANC facilities and, even if their husbands were supportive of antenatal care, the issue often came down to whether there was enough money to go. In some settings these power structures limited ANC engagement but in others women found ways of subverting the hierarchy, especially if they valued antenatal services. "Let me tell you, things are very hard now, my husband does not have money and even when he has, he pretends he does not and will hardly give you anything. It is only when I am going to the hospital that he gives me money and often times I will tell him an amount more than I will pay in the hospital and use the rest for other things" (Umeora 2008, Nigeria). Studies conducted in Pakistan and Bangladesh also revealed that cultural limits placed on women's freedom of movement sometimes restricted their ability to visit ANC facilities. Even in situations where women were convinced of the benefits of antenatal care their inability to travel independently sometimes prevented them from doing so, "I wanted to go for check‐up in the hospital but I could not convince anybody in the house to accompany me. Everybody asked me to stay home" (Chowdhury 2003, Bangladesh).

Also relating to gender, women sometimes felt a sense of shame with being pregnant. In studies conducted in Pakistan and Bangladesh. This was because of an association with sex, whilst in other settings the shame was associated with criticism from health providers or other women about the size of their families or their perceived promiscuity *"*You know the mothers, while sitting down and waiting for the clinic they will start to make comments, "That woman used to roam around and show off now she is here at the clinic" (Andrew 2014, Papua New Guinea). For other women, particularly in studies from South America, the sense of shame or embarrassment was associated with physical examinations, "Mothers do not want nurses to see the vagina, it is very difficult for them, and for that reason I think many mothers do not attend health clinics, it is because of the shame" (Mayca 2009, Peru). This latter issue was of particular concern when the health professional doing routine examinations was male. Some women found this particularly embarrassing “Being palpated by a man, oh, that was worse! That young man who palpated me was actually inspecting my private parts! In fact he frankly told me before, that I must remove most clothes and leave my abdomen exposed. I felt very embarrassed to undress in front of a male stranger” (Maputle 2013, South Africa). For others the preference to be seen by a female health professional was related to a sense of affinity or gender kinship, "I didn’t trust him. The health worker who checked me was a man…that’s why I only went once…I only trust the female workers. I am scared of going. Because I’m older, I want to go [to ANC] this time, but I will have to do without it" (Ayala 2013, Peru).

#### Domain two: Service philosophy, design and provision

The second domain affecting use of ANC services incorporates a number of organisational factors as well as the philosophical approaches underpinning service provision. These include the local infrastructure, the direct and indirect cost of services, the actual clinic environment, the organisation of services, resource issues and working conditions and an over‐emphasis on risk.

##### Local infrastructure

The proximity of a clinic acted as both a barrier and a facilitator to ANC access, depending on where it was located. For some women, the convenience of having a clinic close by was viewed very positively, “It’s actually quite convenient ‘cause I can walk there [from work] on the nice days. ... It’s close to my husband’s work as well" (Sword 2012, Canada); while for others, particularly those in more rural locations or for those with relatively modest incomes, the inconvenience of getting to a clinic was perceived negatively, "If the obstetric care was located here in the neighbourhood, it would have been better. And the person who does not have a car, how do they get to the specialist unit?" (Cabral 2013, Brazil). Proximity of ANC services was also noted by midwives in a rural area of Nigeria where the creation of a 'grassroots' health centre serving the local community appeared to have a positive effect on maternal and infant morbidity (Chimezie 2013, Nigeria).

In some LMICs, where women faced the prospect of making relatively long journeys (sometimes on foot) to reach an ANC clinic, the local infrastructure could have a negative impact, "I never visited the health center to check my pregnancy because it is so far and the road condition is too bad" (Matsuoka 2010, Cambodia). These areas were often devoid of useful and affordable public transport systems, making travel to ANC clinics even more difficult, "There were cars but they were all full. I waited for a while but it was getting late so I started to worry how I would get back afterwards, so I just decided not to go to clinic" (Andrew 2014, Papua New Guinea). Transport difficulties were also recognised by providers, particularly in low‐income settings where the hazardous terrain in some rural areas restricted ANC access and presented serious safety concerns for women in distress, "Because of muddy and difficulty topography, the pregnant women in remote areas will not be able to be picked up by the ambulance car from their home. Thus, we have to carry them… This is one of the problems that we have to deal with until the road is constructed" (Bradley 2012, Ethiopia). Providers working in rural areas also bemoaned the lack of available transport options to take them to and from work and the effect this had on the service they were able to provide, "There are no transports for nurses; the authorities should make transportation available for us. We need transport so that we can come early and give effective focused antenatal service, then.... I think the big people should think seriously about it because it will bring more productivity" (Baffour‐Awuah 2015, Ghana).

##### Cost of services

Although publicly‐funded ANC services are provided free of charge in almost all countries around the world, the indirect costs of getting to and from clinics, the additional charges associated with the purchase of medicines, the loss of vital income to families who rely on women's contributions and the corruptive practices of some healthcare staff all limited women's engagement with ANC. Our findings showed that even when women were convinced of the benefits of ANC and lived in an area where there were no infrastructure issues, if they did not have the money to pay for transport they could not go, "The problem is I did not have any money to pay the transport. I want to have my pregnancy checked by the doctor or the midwife every month, but their places are so far away. I needed transport to get there. Instead, I went and sought traditional birth attendants" (Titaley 2010, Indonesia). In relatively impoverished settings the costs of getting to and from a clinic were sometimes overtaken by more immediate concerns relating to women taking time off from family duties or vital income‐generating activities, "When I had a third pregnancy, it was harvest season. So I wanted to help my husband, even during the pregnancy" (Matsuoka 2010, Cambodia). Even in fairly affluent countries the additional costs of purchasing essential medicines or tests hampered ANC attendance in contexts where women were living in relative poverty, “The doctors got angry with me because they wanted me to have an ultrasound but I did not have money” (Coverston 2004, Argentina). Although these issues were occasionally compounded by corrupt healthcare employees selling medicines to women that were supposed to be supplied free of charge (Rahmani 2013, Afghanistan), a number of healthcare providers in a variety of settings also recognised the indirect costs of ANC attendance as a potential barrier to access, "The pregnant women living in rural areas have financial and time constraints for examination [since they need to work]. I have to explain to them that they might experience complications affecting themselves and their unborn child during their pregnancy" (Graner 2013, Vietnam).

##### Clinic environment

In situations where women made the decision to visit an ANC facility and had the time and resources to do so, the environment they encountered at the clinic could have a significant impact on their willingness to return. In a number of settings, including high‐income countries (HICs), the amount of time women were kept waiting was hugely disproportionate to the amount of time they actually spent with a health professional, and generated feelings of frustration and resentment, "I mean I have waited so long and I was thinking, oh, why do they even give you an appointment time because I am never in there on my appointment time. I normally have to wait for an hour and it is so frustrating and then you are only in there for what, five minutes?". (Worley 2004, New Zealand). The issue of time was just as important to health professionals as it was to women, and midwives in a variety of contexts expressed their frustration with the lack of time available at each appointment, "When they [mothers] are many you don't attend to them. You simply examine her, you listen to complaints, you don't treat, there is no time" (Franngard 2006, Uganda). Health professionals recognised the importance of the antenatal appointment as an opportunity to establish meaningful relationships with women beyond the tick box requirements of a formal antenatal consultation, "Women want time. They want to be able to talk about what they are doing, and for women who aren’t educated, don’t know the right questions, or how to say things, it often takes a lot of time just sitting with them to open to the point where they will talk about a bad discharge smell or… the baby hasn’t been moving for the last two days … It really is having enough time to get to know the woman and for them to feel like they are welcomed and they are listened to and they are not hurried out." (Heaman 2015, Canada), Both midwives and women also agreed that a lack of privacy in busy clinics sometimes discouraged women from further attendance, *"...*.if I go to the clinic, there are so many other people sitting there. Everybody is listening to what you are telling the nurses…sometimes, there are things you want to tell only the nurse or you want to ask the nurse alone. But because there are other patients, you can’t" (Ganle 2014, Ghana). In several LMICs providers felt that the condition of the clinic itself acted as a deterrent to women's attendance and, in some cases, was not fit for purpose, *"*Just look at the building. You cannot tell it is a health centre, the health centre is . . . remote . . . the working conditions are poor, there is no transport, no telephone . . . It becomes sad if you have an emergency and you cannot call an ambulance. At times you watch patients dying and you cannot help in any way" (Mathole 2005, Zimababwe)

##### Organisation of services

Both women and providers felt that in certain settings, particularly LMICs, ANC services were poorly organised and hampered regular attendance. A study in Uganda, for example, revealed that whilst antenatal appointments were offered on a daily basis, antenatal education sessions were only offered twice a week on an ad hoc basis, so women had no idea when to attend or what was being taught. “We ask them to return after one month; in between, we do not follow it up. So when they come, the topic they find is the one they shall listen [to], but we do not repeat.” (Conrad 2012, Uganda). Of more concern to both providers and women in a variety of settings was the flexibility of appointment times and the availability of health professionals. In some contexts appointment systems were organised with a provider focus so that heavy caseloads could be managed more effectively, but these systems were not necessarily convenient for women, "They come and we give them dates, except Thursdays . . . normally Thursday is not a working day in this community, so to them Thursday is a clinic visiting day . . . Even when given a date, they wait for Thursday . . . they never observe the dates" (Mathole 2005, Zimababwe). In contrast, where appointment systems were deemed to be more flexible or where health professionals were perceived to be more available women appreciated the ease of access and the extra reassurance this provided, "I think being able to call and get somebody to call you back in about 10 or 15 minutes has been really great. I think that ‐ I don’t know that I wouldn’t have had as healthy a pregnancy ‐ but I think I would’ve felt a little bit more stressed out about certain things" (Sword 2012, Canada).

##### Resource issues and working conditions

Although some women in LMICs bemoaned the lack of equipment, medicines and supplies at local health facilities and viewed this as a disincentive to ANC engagement (Conrad 2012; Matsuoka 2010; Shabila 2014) issues relating to resources and working conditions were largely highlighted by health professionals. Poor pay, lack of career progression opportunities and a lack of recognition were cited by health professionals in a number of LMICs, “We are paid less by the state government and also there is no promotion, no bonus or reward, and the salary is not enough for us to feed our families” (Mugo 2018, South Sudan). Staff shortages were a particular issue and, although identified in one or two high‐income settings (Alderson 2004, UK; Novick 2013, USA), the most severe shortages were noted in LMICs, especially Africa. "Understaffing is a problem, just now I cannot go for a home visit . . . I cannot go because there will be no‐one. I can’t go off . . . I am always here. I work throughout the day and night" (Mathole 2005, Zimbabwe). For some health professionals the lack of staff, coupled with a heavy workload, generated feelings of frustration and anger and the desperate acknowledgement that women were inevitably receiving sub‐standard care, "You are doing research on maternal health access…you have been here, you have seen our staff strength and you have seen the kind of resources and equipment we are working with. How can we ensure that all women have access to good care? Just look at me, I am the only midwife, and look at all the women sitting outside, how can one person take proper care of all of them. Sometimes, I believe the women are right for not coming to us" (Ganle 2014, Ghana). Health professionals also complained of inadequate training, particularly in their ability to deal with pregnancy complications, whilst more experienced staff felt the opportunities for much‐needed refresher courses or 'updates' were curtailed by limited resources, “We hardly go to any training or workshops nor do we receive any tuition reimbursement or bursary for advanced education.” (Chimezie 2013, Nigeria). In addition, poor working conditions and shortages of relatively basic equipment and supplies contributed to inadequate care in a number of LMICs, "We have no essential equipments such as a weighing scale or labour kits for childbirth. We have stopped providing DPT‐ Hepatitis B vaccine because we have no syringes" (Mrisho 2009, Tanzania). In situations where staff felt supported by their managers they felt better able to deal with the various challenges they encountered, *"*We get huge assistance from the woreda [local government].They supervise every week…by mobile phone and by presenting themselves at the health center. There are annual, quarterly, weekly action plans. They follow up on the implementation of these activities. There are experts assigned to provide support for us" (Bradley 2012, Ethiopia). However, where these relationships were strained or viewed as unsupportive, health professionals became frustrated and disengaged, *"*The first thing that people from the woreda [local government] and the health center ask us when they come here is ‘how many babies did you deliver?’ But there might be bleedings, and we don’t even have gloves here. We can’t even get any gloves when we go and ask for them… We are always asking and we are saying that we are missing these things… They do not even supply gloves. We always raise the problem, and the woreda always skip it" (Bradley 2012, Ethiopia).

##### (Over‐) emphasis on risk

In several countries (the UK, Zimababwe, Uganda and Tanzania) health professionals felt that the use of screening procedures to determine risk status hindered their ability to deliver quality antenatal care. Midwives felt that the amount of time required to complete all of the necessary screening procedures during a relatively short antenatal appointment left little time to discuss any woman‐initiated concerns or offer genuine care, "It is the dilemma we are grappling with, and personally I think screening has been introduced without the resource commitment being taken on board" (Alderson 2004, UK).

#### Domain three: What matters to women and staff: personalised supportive care

The third domain encompasses key aspects of antenatal care that are important to women and staff. The first of these is personalised, supportive care incorporating social and community support, individualised care and staff attitudes.

##### Sub‐domain 3a: Personalised, supportive care

###### Social and community support

In a number of different settings and contexts women highlighted the importance of a social component to antenatal care. Several studies conducted in rural areas of LMICs, where ANC access is traditionally low, discussed community involvement in the design and provision of ANC services. In the Huanaco region of Peru, women from the indigenous community were not only engaged in informing the content of ANC (including recognition of traditional practices), but were also involved in the design of the health facility itself to ensure it was constructed along traditional lines, "... We were consulted about the construction of the maternity house in Yápac and we took the ideas and after we all engaged in building it, the people participated bringing materials: boards, stones, sand, bricks, and all that is needed" (Mayca 2009, Peru). In India the use of community‐based 'women's groups' generated interest and input into maternity care and a genuine force for change, "As for my knowledge, the people who are attending the meetings and discussing many new things about the health of mothers and newborns are explaining what they have learnt to five more people, as a result of which each and every person should know. These meetings are really helpful as we are only involved in trying to solve the health problems of the community through the help of community members. We believe that together we can bring about change". (Rath 2010, India). The value of engaging with other pregnant women in an informal way was highlighted by women in a wide variety of contexts and circumstances and is exemplified by this quote from a pregnant woman in Nigeria, "Doctor, you know that we engage in 'hard' work everyday, it is only when we come here or visit the local midwives (TBAs) that we have time to relax and enjoy, even you meet other pregnant women like you and talk about many things that will help you and the baby'. Don't you know we enjoy this dance each time we come here, in fact I look forward to it. If you ask me to come only four times that means I will come only four times. No! I enjoy dancing and other women will agree with me. It helps us relax and make the baby in your 'stomach' (uterus) active and healthy" (Umeora 2008, Nigeria) This kind of social engagement was also evident in a number of HICs, although largely mediated through group antenatal care. The group format provided a context for social interaction and was largely welcomed by women and healthcare professionals as a place where women could share pregnancy‐related information and receive valuable emotional and psychological support, "I felt good, because like, it was good to talk to somebody that was in your predicament, which was pregnant. It was good to talk to somebody like that, so they could understand where you coming from, and how you feeling too" (Novick 2013, USA).

###### Individualised care

Women in HICs sometimes felt that antenatal appointments were impersonal interactions devoid of any genuine 'care'. The short duration of appointments coupled with the emphasis on clinical measurements, largely focused on the foetus, left women feeling processed rather than cared for, "Yeah cos everything is about the baby...it's like AAARRGGHHH! No one says 'how are your hormones today?' or 'can you poke your head out of the hole today?' Yeah, I'm doing well....or they say it as in 'how's your tummy going? It's not about YOU...and how's your BRAIN getting around it!" (Armstrong 2005, Australia). In contrast, women recognised when health professionals provided genuine care and appreciated the individualised nature of inquiries, "She just explained the whole process and she offered me the options of the CMU [community midwifery unit] or the Consultant led unit and explained them in detail and again we just talked through any of my anxieties" (Docherty 2011, UK). Both women and providers in a variety of HICs recognised that the 'continuity of carer' model was probably the best way of providing the type of individualised care that women wanted. This view was expressed in positive terms, "If you were worried about anything or wanted to talk about anything, it's easier if you see the same person every time rather than a strange face" (Earle 2000, UK), or in negative terms, "I worked in Antenatal Clinic for three months. Back then, it just struck me that it was such a waste of time. These poor women would come and sit around for hours, waiting and then they would be seen for five minutes and the person seeing them wouldn’t even know their name." (Wilmore 2015, Australia).

###### Attitude of staff

In terms of women's engagement with ANC the attitude of staff played a key role. In situations where healthcare staff were perceived to be kind, attentive and empathic women were much more likely to return, "When I visit her I feel relaxed, I feel less pain because I like her. She asks me about my problems, I tell her and she answers to all my questions. She talks about everything and she explains everything" (Kabakian‐Khasholian 2000, Lebanon). However, in settings where staff were perceived to be cold and impersonal or just plain rude, women felt upset and sometimes unwilling to return, "[The health workers] work well, but last time I went for ANC they upset me. She told me ‘old woman why are you giving birth to more children? You should use contraception […] I told you and you did not listen to me." (Ayala 2013, Peru). In a number of LMICs the impersonal nature of care sometimes descended into disrespectful behaviour and occasionally verbal or even physical abuse “I am also afraid of the nurses. They bully and mistreat us" (Pretorius 2004, South Africa). From a provider perspective there was an acknowledgement that they sometimes resorted to disrespectful behaviour, although they usually sought to justify their actions. A fieldwork observation from a study in Uganda highlights this issue, "During fieldwork, incidents where caregivers were unfriendly in their interaction with patients were also observed. One such incident was observed in a queue outside a congested health facility in which the caregivers shouted at the waiting patients and even physically pushed away those whom they said were not following the rules. The caregivers said this was the only way to handle what they described as ‘stupid women" (Ayiasi 2013, Uganda). In some contexts this disrespectful attitude amongst providers was so pervasive that suspicions were aroused when health professionals acted in a caring manner, "I am sad to say that patients are afraid of us, they do not dare to ask questions. If I take good care of my patient, my colleagues ask if I am related to the patient or have received money from her" (Rahmani 2013, Afghanistan).

##### Sub‐domain 3b: Information and safety

The third domain also encompasses issues that are important to women and staff, and focuses on antenatal care as a source of information and as a context for clinical safety.

###### Antenatal care as a source of information

In many countries and contexts women visited ANC providers to acquire knowledge and information about their pregnancy and birth. The quest for information was highlighted by women of all parities but was particularly pertinent for women who were pregnant for the first time, “I think the information that I received was very valuable... very helpful detailed information, especially preparing for the labour part... I didn’t know what to expect, so it was really helpful to be able to get information about those things,” (McNeil 2012, Canada). In situations where information was provided in a useful, appropriate and engaging manner, it generated a sense of empowerment and made women feel more involved in their antenatal care, "I believe it’s the way they involve you, and the way they tell you everything that’s going on. So there’s no secrets, there’s no mysteries, there’s no secret codes or anything like that that you don’t understand. ... It makes you feel like you are totally in the loop and you know just as much as the doctors know. ... And it makes you more confident, and like more prepared, and just feels good to know everything that’s going on" (Sword 2012, Canada). By contrast, in situations where this approach was not adopted, i.e. where tests were not explained properly or information was infused with medical jargon, it acted as a barrier and sometimes curtailed further engagement, "The woman that we spoke to, she was going on about you know about protein in your urine or whatever and all this stuff and I just didn't have a clue what she was talking about. It is all very...... I know they must do it all the time" (Docherty 2011, UK). In some contexts it was not so much the manner in which informational needs were met (or unmet), but was more about the medium used to deliver information. Women did not appreciate being given copious amounts of leaflets or booklets during antenatal visits without the opportunity to discuss the contents with a health professional, "Today they gave me a whole bag of pamphlets and flyers and didn’t explain or go over them with me*"* (Kraschnewski 2014, USA). This was an issue in a couple of high‐income settings and often resulted in women turning to the Internet in search of clarity or to satisfy any informational deficits. In low‐ and middle‐income settings women were more likely to turn to relatives, friends or TBAs to address any unmet informational needs. Sometimes this approach brought clarity or reassurance, but at other times clinical knowledge was supplanted by traditional understandings that perpetuated informational myths, “I was told by my mother that I should stop (having sexual intercourse) when I was seven months pregnant, that when you sleep with a man in late pregnancy you will deliver a baby which is dirty with a bad skin” (Ayiasi 2013, Uganda). For some women living in rural areas of LMICs, where access to formal antenatal care was supplemented by informal visits to TBAs or community midwives, the conflation of different sources of information could be confusing. However, there was evidence that in these situations women, especially younger women, were more likely to value the 'scientific' information derived from healthcare professionals than the 'experiential' knowledge from traditional informants, "If the information from different sources is not the same, I need to discuss it. Three to four women can consult together in the market. If we cannot know who is right, we will follow [the advice from] doctors… The information from parents and grandparents is just experience" (Graner 2013, Vietnam).

###### Antenatal care as a context for clinical safety

In addition to viewing antenatal care as a source of information, women also acknowledged that antenatal appointments provided a context for clinical safety. For women in a variety of different resource settings the availability of medicines, medical tests and screening procedures (e.g. HIV tests and ultrasound) offered safety and reassurance during pregnancy and encouraged ANC attendance, *"*I think for me the most important aspects would be knowing that I’m okay. So knowing that my blood pressure’s okay. And knowing that the baby’s heartbeat is ‐ I can hear it, and it’s same as always. ... And knowing that, say for instance, the size of my uterus is the average size of everybody else’s uterus, right, so at this time of pregnancy. So I would just say kind of being reassured that all my vitals, the baby’s vitals are all fine" (Sword 2012, Canada). For women in LMICs who might not ordinarily access antenatal care, the recognition of a pregnancy‐related problem or complication sometimes prompted a visit, "I would not have gone for check‐up if I did not have pani bhangga (leaking membrane) from the sixth month of my pregnancy. I thought that I didn’t require any check‐up if I wouldn’t have any problem" (Chowdhury 2003, Bangladesh). The experience of a previous pregnancy complication encouraged women in a couple of LMICs to ensure they attended antenatal care early and regularly in subsequent pregnancies, as noted by a health professional in Kenya, “She will attend antenatal care immediately she senses that she is pregnant again. She will start preparing for antenatal care without wasting time because she does not want to lose that child as she has been doing again and again.” (Family Care International 2003, Kenya). Providers in several different contexts also agreed that women were attracted by specific components of antenatal care, especially those offering safety and reassurance, e.g. the availability of iron tablets to prevent anaemia (Graner 2010, Vietnam).

### The line of argument and hypothesised facilitative mechanisms of effect

The line of argument emerging from the analysis of the data relating to pregnant women was as follows.

For women, initial or continued use of antenatal care depends on a perception that doing so will be a positive experience. This is a result of the provision of good‐quality local services that are not dependent on the payment of informal fees and that include continuity of care that is authentically personalised, kind, caring, supportive, culturally sensitive, flexible, and respectful of women’s need for privacy, and that allow staff to take the time needed to provide relevant support, information and clinical safety for the woman and the baby, as and when they need it. Women’s perceptions of the value of ANC depend on their general beliefs about pregnancy as a healthy or a risky state, and on their reaction to being pregnant, as well as on local socio‐cultural norms relating to the advantages or otherwise of antenatal care for healthy pregnancies, and for those with complications. Whether they continue to use ANC or not depends on their experience of ANC design and provision when they access it for the first time.

For healthcare providers, the line of argument was similar, but with a different emphasis.

The capacity of healthcare providers to deliver the kind of high‐quality, relationship‐based, locally accessible ANC that is likely to facilitate access by women depends on the provision of sufficient resources and staffing, as well as the time to provide flexible, personalised, private appointments that are not overloaded with organisational tasks. Such provision also depends on organisational norms and values that overtly value kind, caring staff who make effective, culturally‐appropriate links with local communities, who respect women’s belief that pregnancy is usually a normal life event, but who can recognise and respond to complications when they arise. Healthcare providers also require sufficient training and education to do their job well, as well as an adequate salary, so that they do not need to demand extra informal funds from women and families to supplement their income, or to fund essential supplies.

The three facilitative mechanisms of effect arising from these lines of argument were:

- Treating pregnancy as a fundamentally healthy state while monitoring for complications;
- Ensuring authentically accessible and affordable access to skilled care provision and required resources throughout the antenatal episode;
- Creating the conditions to enable positive staff attitudes and behaviours.

### Testing the findings with 'theory of planned behaviour' logic models

To test the utility of the findings for future use in practice, we developed theoretical logic models based on these findings, to explain no uptake, partial uptake, and full uptake of ANC services by women, in the context of our a priori behavioural theory (the theory of planned behaviour). Each input box was populated by statements based directly on the findings. The three models derived from this process are given in Figure 4, Figure 5 and Figure 6. Text in regular font relates to pregnant women, and text in bold font relates to providers of ANC. Superscript text refers to the finding numbering in Table 1; Table 2; Table 3 and Table 4. For this theoretical exercise, we only used findings of moderate or high confidence. If the logic models and findings are to be used to understand mechanisms of effect for implementation projects in specific settings, they may need to be re‐rated for those specific settings. For example, we rated some findings as low or very low confidence on the grounds of coherence or relevance, because all the data only came from particular settings, or because there was incoherence between different types of settings, or both. Both relevance and coherence may be increased for very specific settings. For example, the low‐confidence rating for ‘Only visit ANC to get an ANC card’ is due to lack of relevance and coherence for all settings, since all five included studies were from Africa. For African settings, however, there is high coherence and relevance for this finding.

**4:**
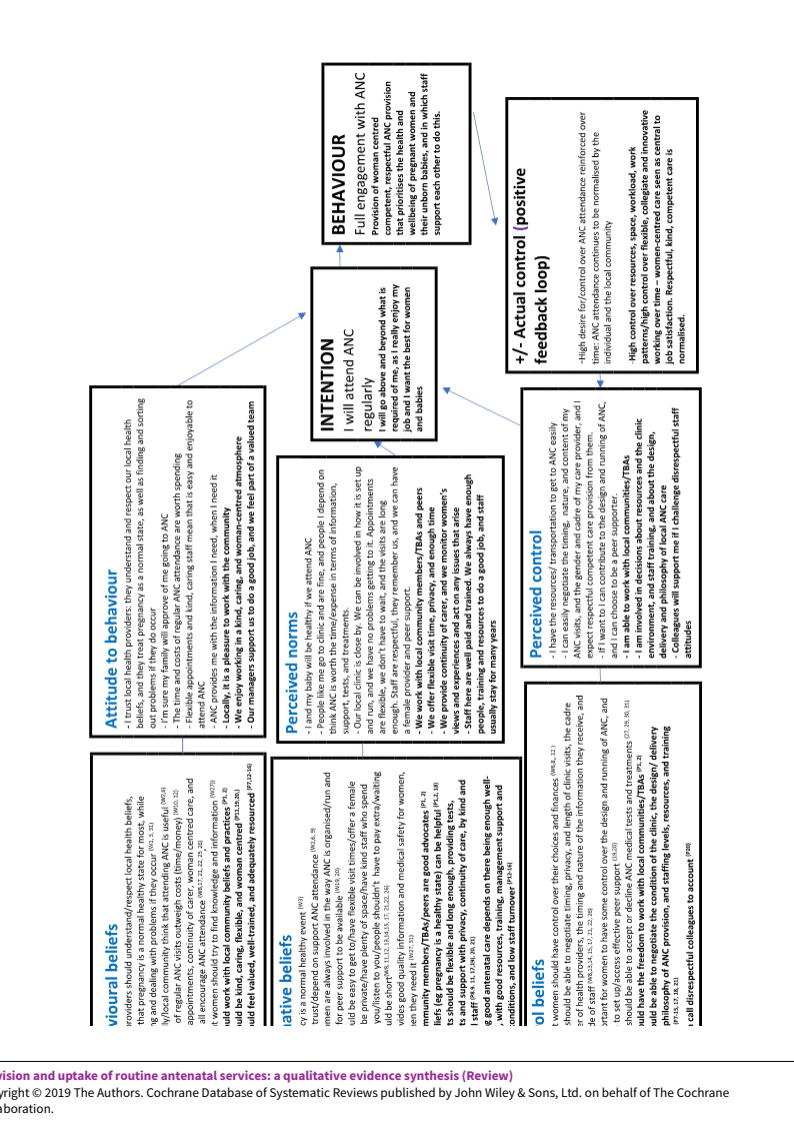
Logic Model of **FULL ANC Uptake** using findings relating to beliefs (superscript letters and numbers refer to Summary of qualitative findings table above)

**5:**
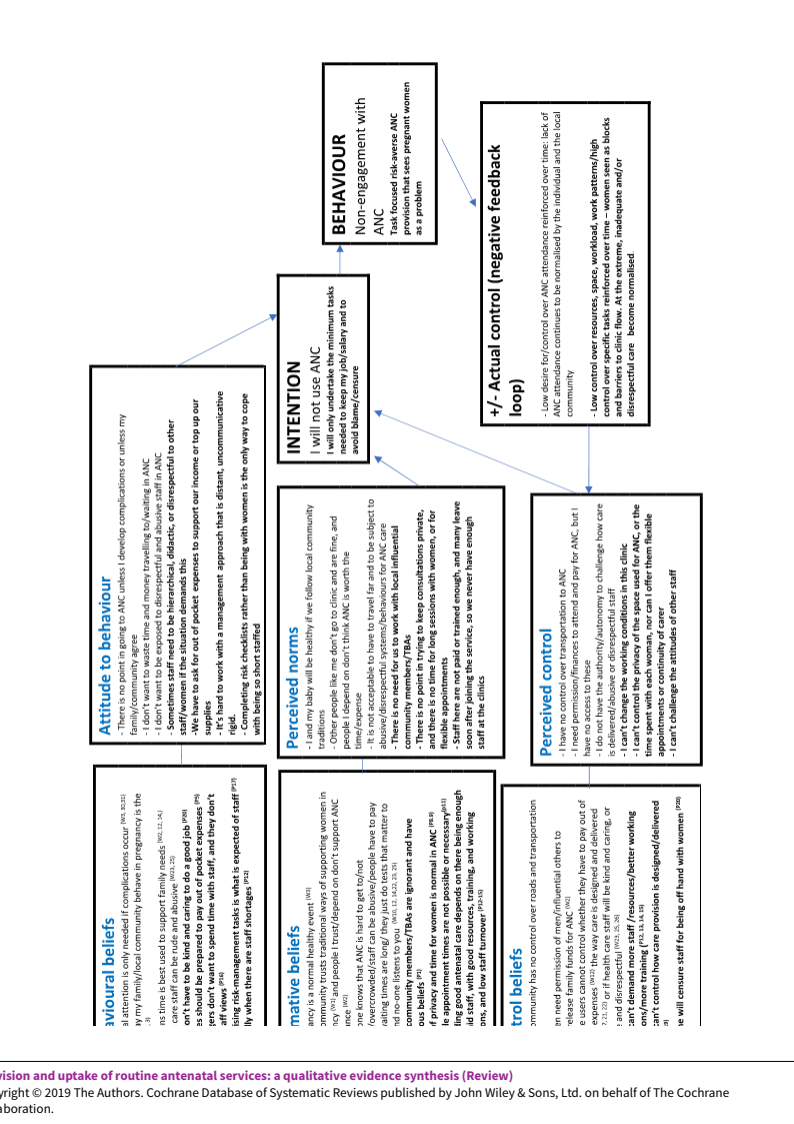
Logic Model of **NO ANC Uptake** using findings relating to beliefs (superscript letters and numbers refer to Summary of qualitative findings table above)

**6:**
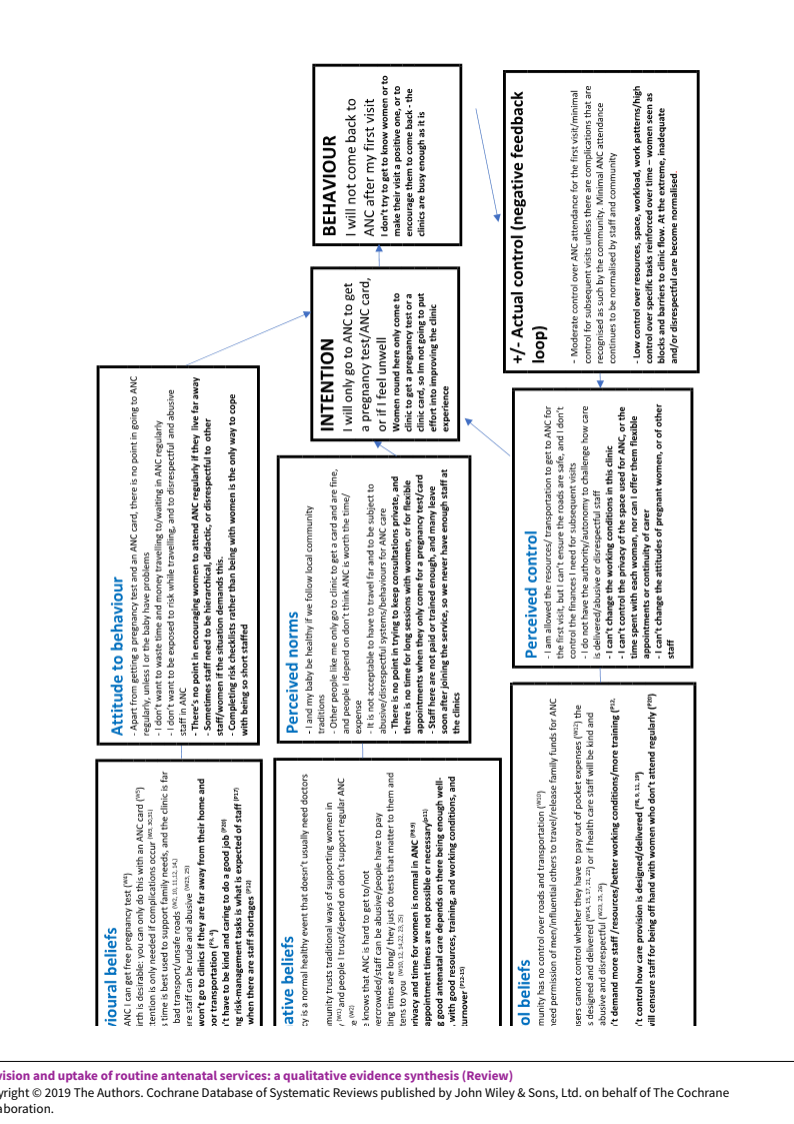
Logic Model of **INITIAL ANC Uptake** using findings relating to beliefs (superscript letters and numbers refer to Summary of qualitative findings table above)

### Using the data for local implementation planning

This modelling exercise illustrates the potential to use the findings of the review as a basis for planning and development with local stakeholders (e.g. policy‐makers, professionals, women, communities, funders). Collaborative assessment of the local position in relation to each of the findings given in the 'Summary of findings' tables, mapped against the elements of the theory of planned behaviour, would illustrate mechanisms where there are local blocks and barriers or potential facilitators, and at which level of the system they are operating (community norms, personal norms, norms of providers, or other). Having identified which findings are most relevant locally, and having agreed any other factors that might be operating in their specific setting, stakeholders can work with the model and with the appropriate findings to turn barriers to facilitators, and to reinforce facilitators that already exist. This would enable the health system to direct effort most efficiently at the factors that are most likely to be influential in catalysing positive change.

### Results of linking the review findings to intervention reviews

We examined the six relevant Cochrane Reviews identified in Table 5 to see if the authors paid attention to possible underlying theories or mechanisms of effect that might influence the effectiveness of the interventions they were examining. Where authors identified any relevant theories or mechanisms, we mapped these to the findings identified in this review (Table 6). Some authors explicitly noted relevant factors in the ‘How this intervention might work’ section of their reviews. In some cases, these mapped directly to some of the findings identified in our review; principally those to do with resources, and with continuity of care. However, 29 findings (women or healthcare provider) were not represented in any of the interventions tested in any of the studies, including four assessed as having high confidence (‘pregnancy as a healthy state’; ‘rude and abusive staff’; ‘authentic and kind staff’; and ‘staff attitude’).

### Results of review author reflexivity

We set out the prior positions of the review team in the Methods section of this synthesis. These positions did not change throughout the synthesis. In terms of data extraction, analysis, synthesis, and decisions about recommendations for practice and research, we specifically looked for disconfirming data relating to our strong prior beliefs that an over‐emphasis on clinical testing and screening tended to overlook women's needs for more psychosocial and informational support. Despite trying to find such disconfirming data, our final analysis confirmed our prior position.

## Discussion

### Summary of main findings

Our primary analysis generated 31 findings relating to women’s experiences and views (17 moderate to high confidence), and 21 relating to healthcare providers (13 moderate to high confidence). The data in the studies included in the updated searches between 2014 and 2019 could all be mapped to the original 31 findings statements, with some additional nuances, and with no new or disconfirming data. This provided a confirmability check for the primary findings.

Three key domains encompassed all of the findings across both groups. These were: 'Socio‐cultural context'; 'Service, design and provision'; and 'What matters to women and staff'*.* The third domain was subdivided into two conceptual areas; personalised supportive care, and information and safety. Logic models were successfully developed, based on the Summary of Qualitative Findings.

### Overall completeness and applicability of the evidence

The included studies encompassed a wide range of countries and socio‐economic settings, and data from women and from healthcare providers. Our confirmatory search and analysis did not identify any new data that could refute the findings from our primary search and analysis.

Eleven findings were present for both women and healthcare providers. They indicate that both women and providers were conscious that ANC was provided in a social context, in which the local social norms could operate either to enhance or to resist uptake. Resource issues were also noted, as well as the need for well‐organised services that offer safety, appropriate information, and positive interpersonal relationships, notably through continuity of care/carer.

In contrast, notions that pregnancy is generally a healthy state, and that attendance at ANC was only useful to confirm pregnancy, or to get an ANC card and therefore access to facilities for intrapartum care, were only present in the women’s data. Data relating to personal constraints, including issues relation to gender inequality (financial dependence. shame and embarrassment, freedom of movement) were also unique to women’s views and experiences. Findings relating to working conditions, training, and the need for management support were only in the healthcare providers' data. Although the finding relating to pregnancy as a healthy state was not in the providers' data, a parallel finding about the perceived limits of risk‐averse service provision was generated from provider responses.

Some authors of effectiveness studies in the area of antenatal care design and provision identified mechanisms of effect, programme theories, or design features for their chosen interventions that could be mapped directly to some of the findings identified in this review. These were principally to do with resources, and with continuity of care. The four high‐confidence findings that were not overtly linked to the interventions covered two key domains. These were women's perception of pregnancy as a healthy state, and the attitudes and behaviours of staff. Both seem to be important omissions, given the growing evidence that lack of (re‐) attendance for ANC can at least partly be explained by a lack of tailoring of the focus of ANC on what matters to women (Downe 2016a; Finlayson 2013; WHO 2016).

The capacity of the theory of planned behaviour to explain our findings suggests that the results of this review could be relevant for implementation strategies to support the introduction of the WHO ANC guidelines (WHO 2016) into effective practice. Olivier de Sarden 2017 has recently critiqued ‘travelling models’ of maternity care, based on ‘miracle mechanisms’, that, having worked in one (usually high‐income) setting, are deemed to be effective for implementation as whole programmes in completely different contexts. De Sardan argues, based on experiences in Africa, that effective implementation needs to first establish local systems, norms, and values, and then to build up context‐specific programmes that are culturally normative for each setting. As our findings have revealed, the need to understand local cultural framing of ANC provision, which may be radically different from normative cultural framing of donors or international agencies, has indeed been recognised in existing qualitative studies of the views and experiences of pregnant women and healthcare providers.

In line with these prior analyses, our review has identified factors rated as low confidence, since they may be more applicable in some particular settings than others. These include the need to visit clinics in pregnancy to get an ANC card as a ‘passport’ to facility birth, for example. In these particular settings, such findings may actually reach high confidence. Issues such as this (going to ANC only to get a card, in an African context) could have been explored in greater detail through sub‐analyses of the data, particularly with regard to potential differences between service provision and uptake in HICs and LMICs. Similarly, our findings might have been further enriched if we had explored similarities and differences between types of respondents, e.g. between midwives and doctors, or types of service provider, e.g. public and private. Future updates of this review could specify these sub‐analyses a priori.

However, our results also provide a critique of the premise that maternal health interventions can ONLY be built from the bottom up. Whilst there will always be a need for local tailoring, the three key domains identified by our review do seem to be universally applicable, both on the basis of the review data and in terms of our confirmatory analysis. We suggest that ANC implementation strategies should always be structured by these three domains, in any context, with the findings arising from the review acting as a framework for assessing what might be relevant locally within each domain, and to what degree each of the findings need to be addressed locally to maximise effective local implementation.

### Confidence in the findings

In the primary analysis, we had high or moderate confidence in more of the findings that contributed to the domain of ‘What matters to women and staff’ (personalised supportive care, and information and safety), (11/17 findings), than for ‘Service design and provision’ (13/28 findings) or for ‘Sociocultural context’ (4/10 findings). This reflects other qualitative analyses of women’s views in other areas of maternity care (Downe 2018; Karlström 2015). For the providers, high or moderate confidence was also evident for findings relating to staff working and employment conditions, which is in line with other studies of healthcare provider views in a range of maternity and general healthcare areas and settings (Elbarazi 2017; Munabi‐Babigumira 2017).

### Agreements and disagreements with other studies or reviews

We are aware of four published meta‐synthesis studies related to antenatal care provision (Table 9). They all focus on the views and experiences of service users. Two included only women who did not use ANC at all, or who did not use it regularly (Downe 2009; Finlayson 2013). One only included women using ANC in the USA (Phillippi 2009). One focused on what matters to women in pregnancy in general, and not on their actual experiences of ANC (Downe 2016a). The findings of our review include providers; women who used ANC fully as well as those who did not; and accounts of participants from around the world. All of the findings in the previous four meta‐synthesis reviews could be mapped to the three key domains generated by this review, with some additional context‐specific details, such as the need for provision of childcare in ANC settings in a review based on participants from the USA (Phillippi 2009).

**5:** Existing qualitative reviews in the area of routine antenatal care for healthy women and babies

**Authors, date**	**Title**	**Focus**	**Methodology**	**What the current review adds**
Downe 2009	'Weighing up and balancing out': a meta‐synthesis of barriers to antenatal care for marginalised women in high‐income countries	Exploration of women's views and experiences of non‐use of ANC in HICs	Qualitative meta‐synthesis	A wider scope, as this review includes all women from all settings, and includes facilitators as well as barriers
Finlayson 2013	Why do women not use antenatal services in low‐ and middle‐income countries? A meta‐synthesis of qualitative studies	Exploration of women's views and experiences of non‐use of ANC in LMICs	Qualitative meta‐synthesis	A wider scope, as this review includes all women from all settings, and includes facilitators as well as barriers
Phillippi 2009	Women's perceptions of access to prenatal care in the United States	Exploration of women's views and experiences of access to ANC in the USA	Qualitative meta‐synthesis	A wider scope, as this review includes all women from all settings
Downe 2016a	What matters to women: a systematic scoping review to identify the processes and outcomes of ANC provision that are important to healthy pregnant women	Exploration of what pregnant women might want and need to support them through pregnancy	Qualitative meta‐synthesis	The review excluded women who were reporting on their actual experience of ANC. This review includes these accounts

ANC: antenatal care; FANC: focused antenatal care; HICs: high‐income countries; LMICs: low‐ and middle‐income countries

In terms of the six most relevant published effectiveness reviews, as noted above and in Table 5 and Table 6, there is a variable match between the hypothesised or apparent mechanisms of effect for the proposed interventions, and the findings of this review. Intervention programmes with multiple components were more likely to include more elements that could be mapped to the findings of this review than those with single components. Those planning future antenatal intervention studies or implementation programmes should consider the findings of this review as part of their analysis of the mechanisms of effect that they intend to operationalise through their planned interventions.

### Limitations of the review

Despite extensive efforts to identify studies from all relevant settings, our review has fewer studies from mainland Europe (n = 4) or the Middle East (n = 4), and none from either Russia or China. Most studies were published in English, which may suggest that studies from cultural contexts where English is not the norm were missed; however, our searches were not restricted by language and we searched continent‐specific databases such as LILACS and AJOL. We excluded studies relating to sub‐populations of women, e.g. minority ethnic groups, refugees and asylum seekers, HIV‐infected women and women with drug or alcohol dependency, as we were interested in routine antenatal care services rather than 'additional' services that might be offered to women from these sub‐groups. Although women from some of these groups are likely to be represented in the general populations included in the studies in the review, their specific needs are not addressed, and arguably it is women from these sub‐groups that are most likely to benefit from antenatal care. Similarly, we excluded from the review women with identified pregnancy complications (e.g. pre‐eclampsia, gestational diabetes), as additional support may be required for these women. In some cases where confidence in the findings was low overall, this was because the data only came from particular contexts, such as LMIC settings. Analysis by these settings alone would have resulted in higher confidence ratings for the findings in the context of those specific settings. This may be important if the findings are to be applied to specific settings in the future.

## Authors' conclusions

This review has identified key barriers and facilitators in the uptake (or not) of ANC services by pregnant women, and in the provision (or not) of good‐quality ANC by healthcare providers. It complements existing effectiveness reviews of models of ANC provision (Brown 2015; Catling 2015; Dowswell 2015; Mbuagbaw 2015; Sandall 2016; Till 2015) and adds essential insights into why the particular type of ANC provided in specific local contexts may or may not be acceptable, accessible, or valued by some pregnant women and their families/communities.

### Implications for practice

Stakeholders intending to implement ANC guidelines could benefit from local modelling of the three key domains identified in this review, and their sub‐elements, against the theory of planned behaviour, in relation to their local communities and ANC provision. This would enable stakeholders to identify local normative, belief, or attitudinal barriers to good‐quality provision, and to uptake. Successful implementation will require tailoring of the ANC service to ensure clinical and interpersonal quality, address barriers, and enhance locally‐enabling factors as well as culturally appropriate promotion of good‐quality locally‐tailored services. This groundwork could also reveal facilitative factors that already exist in the relevant communities and health systems that could be maintained and reinforced into the future. The final model will be different in each setting, while remaining faithful to the underlying mechanisms of effect revealed by the findings generated by the review. For maximum effect, this exercise should include community and service‐user stakeholders, as well as service funders, policy‐makers, managers, and providers.

### Implications for future research

Implementation research should be undertaken to test the findings and conclusions of this review, especially those in which there is high or moderate confidence. Specifically, such research could test the utility of the integration of the findings with logic models as a basis for prospective interventions to improve the quality, acceptability, and uptake of antenatal care provision in particular local settings. Lessons learned should be integrated iteratively into subsequent implementation research design in this area. Future comparative and implementation studies in the area of ANC should explicitly state the hypothesised underlying mechanisms of effect of the chosen intervention(s), with regard to the factors identified in this review. Outcomes should be selected to establish if these mechanisms are actually operating once the intervention is implemented, and to assess impacts that are in line with issues important to service users or providers, or both, as revealed by this review.

## Appendices

### Appendix 1. Search strategies

**Ovid MEDLINE(R) and Epub Ahead of Print, In‐Process & Other Non‐Indexed Citations and Daily**1946 to present

**January 2000 – CURRENT (September 2014): Women’s Searches**

**Table:**

**#**	**Searches**	**Results**
1	Prenatal Care/ or Perinatal Care/ or Maternal Health Services/ or Maternal‐Child Health Services/	36401
2	(antenatal care or antenatal service? or antenatal support or prenatal care or prenatal service? or prenatal support or antepartum care or antepartum service? or antepartum support or perinatal care or perinatal service? or perinatal support or maternal care or maternal service? or maternal health care or maternal healthcare or maternal support or pregnancy care or pregnancy service? or pregnancy support).ti,ab,kw.	20772
3	1 or 2	47854
4	(woman or women$ or mother$).ti,ab,kw.	1201726
5	3 and 4	28115
6	qualitative research/	38113
7	7 and 8	867
8	limit 5 to "qualitative (best balance of sensitivity and specificity)"	7829
9	7 or 8	7891
10	limit 11 to yr="2000 ‐Current"	4266
11	Limit 10 to “humans”	4045

**September 2014 – CURRENT (Febraury 2019): Women’s Searches**

**Table:**

**#**	**Searches**	**Results**
1	Prenatal Care/ or Perinatal Care/ or Maternal Health Services/ or Maternal‐Child Health Services/	40221
2	(antenatal care or antenatal service? or antenatal support or prenatal care or prenatal service? or prenatal support or antepartum care or antepartum service? or antepartum support or perinatal care or perinatal service? or perinatal support or maternal care or maternal service? or maternal health care or maternal healthcare or maternal support or pregnancy care or pregnancy service? or pregnancy support).ti,ab,kw.	23991
3	1 or 2	53614
4	(woman or women$ or mother$).ti,ab,kw.	1221256
5	3 and 4	30633
6	qualitative research/	43880
7	5 and 6	988
8	limit 5 to "qualitative (best balance of sensitivity and specificity)"	8290
9	7 or 8	8344
10	limit 9 to yr="2014 ‐Current"	3280
11	Limit 10 to “humans”	2730

**January 2000 – CURRENT (February 2015): Providers Searches**

**Table:**

**#**	**Searches**	**Results**
1	Prenatal Care/ or Perinatal Care/ or Maternal Health Services/ or Maternal‐Child Health Services/	37806
2	(antenatal care or antenatal service? or antenatal support or prenatal care or prenatal service? or prenatal support or antepartum care or antepartum service? or antepartum support or perinatal care or perinatal service? or perinatal support or maternal care or maternal service? or maternal health care or maternal healthcare or maternal support or pregnancy care or pregnancy service? or pregnancy support).ti,ab,kw.	21772
3	1 or 2	49966
4	exp Health Personnel/	446351
5	(Staff or provider$ or health care provider$ or nurs$ or midwife$ or midwives or physician$ or doctor$ or obstet$ or medical professional$ or clinician$ or skilled birth attendant$ or auxiliary or lay health worker$ or community health worker$ or traditional birth attendant$ or tba$).ti,ab,kw.	1213677
6	4 or 5	1449700
7	3 and 6	17809
8	qualitative research/	33348
9	7 and 8	504
10	limit 7 to "qualitative (best balance of sensitivity and specificity)"	4640
11	9 or 10	4666
12	limit 11 to yr="2000 ‐Current"	3668
13	Limit 12 to «humans»	3521

**February 2015 – CURRENT (February 2019): Providers Searches**

**Table:**

**#**	**Searches**	**Results**
1	Prenatal Care/ or Perinatal Care/ or Maternal Health Services/ or Maternal‐Child Health Services/	40064
2	(antenatal care or antenatal service? or antenatal support or prenatal care or prenatal service? or prenatal support or antepartum care or antepartum service? or antepartum support or perinatal care or perinatal service? or perinatal support or maternal care or maternal service? or maternal health care or maternal healthcare or maternal support or pregnancy care or pregnancy service? or pregnancy support).ti,ab,kw.	24,128
3	1 or 2	53376
4	exp Health Personnel/	471,227
5	(Staff or provider$ or health care provider$ or nurs$ or midwife$ or midwives or physician$ or doctor$ or obstet$ or medical professional$ or clinician$ or skilled birth attendant$ or auxiliary or lay health worker$ or community health worker$ or traditional birth attendant$ or tba$).ti,ab,kw.	1,323677
6	4 or 5	1526700
7	3 and 6	19004
8	qualitative research/	42779
9	7 and 8	798
10	limit 7 to "qualitative (best balance of sensitivity and specificity)"	5497
11	9 or 10	5693
12	limit 11 to yr="2015 ‐Current"	1977
13	Limit 12 to «humans»	1701

**Embase ‐ OvidSP 1974 to Present;**

**January 2000 – CURRENT (September 2014): Women’s Searches**

**Table:**

**#**	**Searches**	**Results**
1	Prenatal Care/ or Perinatal Care/ or Maternal Health Services/ or Maternal‐Child Health Services/	41976
2	(antenatal care or antenatal service? or antenatal support or prenatal care or prenatal service? or prenatal support or antepartum care or antepartum service? or antepartum support or perinatal care or perinatal service? or perinatal support or maternal care or maternal service? or maternal health care or maternal healthcare or maternal support or pregnancy care or pregnancy service? or pregnancy support).ti,ab,kw.	22824
3	1 or 2	52331
4	(woman or women$ or mother$).ti,ab,kw.	1476883
5	3 and 4	31229
6	qualitative research/	55467
7	7 and 8	841
8	limit 5 to "qualitative (best balance of sensitivity and specificity)"	9322
9	7 or 8	9871
10	limit 11 to yr="2000 ‐Current"	4917
11	Limit 10 to “excluding Medline”	343
12	Limit 11 to “humans”	336

**Sept 2014 – CURRENT (February 2019): Women’s Searches.**

**Table:**

**#**	**Searches**	**Results**
1	Prenatal Care/ or Perinatal Care/ or Maternal Health Services/ or Maternal‐Child Health Services/	49971
2	(antenatal care or antenatal service? or antenatal support or prenatal care or prenatal service? or prenatal support or antepartum care or antepartum service? or antepartum support or perinatal care or perinatal service? or perinatal support or maternal care or maternal service? or maternal health care or maternal healthcare or maternal support or pregnancy care or pregnancy service? or pregnancy support).ti,ab,kw.	28865
3	1 or 2	61415
4	(woman or women$ or mother$).ti,ab,kw.	1630938
5	3 and 4	35814
6	qualitative research/	60850
7	5 and 6	875
8	limit 5 to "qualitative (best balance of sensitivity and specificity)"	10188
9	7 or 8	10311
10	limit 9 to yr="2014 ‐Current"	4183
11	Limit 10 to “excluding Medline”	413
12	Limit 11 to “humans”	300

**January 2000 – CURRENT (February 2015): Providers Searches**

**Table:**

**#**	**Searches**	**Results**
1	Prenatal Care/ or Perinatal Care/ or Maternal Health Services/ or Maternal‐Child Health Services/	43221
2	(antenatal care or antenatal service? or antenatal support or prenatal care or prenatal service? or prenatal support or antepartum care or antepartum service? or antepartum support or perinatal care or perinatal service? or perinatal support or maternal care or maternal service? or maternal health care or maternal healthcare or maternal support or pregnancy care or pregnancy service? or pregnancy support).ti,ab,kw.	23071
3	1 or 2	53667
4	Exp Health Personnel/	1,261,427
5	(Staff or provider$ or health care provider$ or nurs$ or midwife$ or midwives or physician$ or doctor$ or obstet$ or medical professional$ or clinician$ or skilled birth attendant$ or auxiliary or lay health worker or community health worker$ or traditional birth attendant$ or tba$).ti,ab,kw.	1,666,756
6	4 or 5	2,211,637
7	3 and 6	22,875
8	qualitative research/	56,078
9	7 and 8	660
10	limit 7 to "qualitative (best balance of sensitivity and specificity)"	6363
11	9 or 10	6456
12	limit 11 to yr="2000 ‐Current"	3617
13	Limit 12 to “humans”	3425
14	Limit 13 to “excluding Medline”	247

**February 2015 – CURRENT (February 2019): Providers Searches**

**Table:**

**#**	**Searches**	**Results**
1	Prenatal Care/ or Perinatal Care/ or Maternal Health Services/ or Maternal‐Child Health Services/	49971
2	(antenatal care or antenatal service? or antenatal support or prenatal care or prenatal service? or prenatal support or antepartum care or antepartum service? or antepartum support or perinatal care or perinatal service? or perinatal support or maternal care or maternal service? or maternal health care or maternal healthcare or maternal support or pregnancy care or pregnancy service? or pregnancy support).ti,ab,kw.	28865
3	1 or 2	61415
4	exp Health Personnel/	1,381,246
5	(Staff or provider$ or health care provider$ or nurs$ or midwife$ or midwives or physician$ or doctor$ or obstet$ or medical professional$ or clinician$ or skilled birth attendant$ or auxiliary or lay health worker$ or community health worker$ or traditional birth attendant$ or tba$).ti,ab,kw.	1,687,334
6	4 or 5	2,486,700
7	3 and 6	26,877
8	qualitative research/	60,850
9	7 and 8	744
10	limit 7 to "qualitative (best balance of sensitivity and specificity)"	7597
11	9 or 10	7693
12	limit 11 to yr="2015 ‐Current"	2577
13	Limit 12 to «humans»	2563
14	Limit 13 to exc Medline Journals	168

**CINAHL EbscoHost;**

**January 2000 – CURRENT (September 2014): Women’s Searches**

**Table:**

#	Searches	No of Hits
S1	(MM "Prenatal Care")	4054
S2	(MM “Perinatal Care”)	1221
S3	((MM "Perinatal Care")) OR (S1)	5241
S4	(MM “Maternal Health Services+)	8517
S5	(MM "Maternal Health Services")) OR (S3)	9758
S6	TI antenatal care or AB antenatal care or TI prenatal care or AB prenatal care or TI pregnan$ or AB pregnan$ or TI perinatal care or AB perinatal care	4406
S7	(TI antenatal care or AB antenatal care or TI prenatal care or AB prenatal care or TI pregnan$ or AB pregnan$ or TI perinatal care or AB perinatal care) OR (S5	12262
S8	TI woman$ or AB woman$ or TI women$ or AB women$ or TI mother$ or AB mother$	194,931
S9	(TI woman$ or AB woman$ or TI women$ or AB women$ or TI mother$ or AB mother) AND (S7)	6162
S10	(MM “Qualitative Studies+”)	3933
S11	TI qualitative or AB qualitative or TI focus group$ or AB focus group$ or TI interview$ or AB interview$	108225
S12	(TI qualitative or AB qualitative or TI focus group$ or AB focus group$ or TI interview$ or AB interview$) OR (S10)	110,393
S13	((TI qualitative or AB qualitative or TI focus group$ or AB focus group$ or TI interview$ or AB interview$) OR (S10)) AND (S9	1787
S14	Limit S13 to studies published from January 2000 to Current	1162

**September 2014 – CURRENT (February 2019): Women’s Searches**

**Table:**

#	Searches	No of Hits
S1	(MM "Prenatal Care")	8066
S2	(MM “Perinatal Care”)	3441
S3	((MM "Perinatal Care")) OR (S1)	11408
S4	(MM “Maternal Health Services+)	16273
S5	(MM "Maternal Health Services")) OR (S3)	19489
S6	TI antenatal care or AB antenatal care or TI prenatal care or AB prenatal care or TI pregnan$ or AB pregnan$ or TI perinatal care or AB perinatal care	9590
S7	(TI antenatal care or AB antenatal care or TI prenatal care or AB prenatal care or TI pregnan$ or AB pregnan$ or TI perinatal care or AB perinatal care) OR (S5	24929
S8	TI woman$ or AB woman$ or TI women$ or AB women$ or TI mother$ or AB mother$	364,975
S9	(TI woman$ or AB woman$ or TI women$ or AB women$ or TI mother$ or AB mother) AND (S7)	13230
S10	(MM “Qualitative Studies+”)	6458
S11	TI qualitative or AB qualitative or TI focus group$ or AB focus group$ or TI interview$ or AB interview$	206352
S12	(TI qualitative or AB qualitative or TI focus group$ or AB focus group$ or TI interview$ or AB interview$) OR (S10)	209742
S13	((TI qualitative or AB qualitative or TI focus group$ or AB focus group$ or TI interview$ or AB interview$) OR (S10)) AND (S9	2500
S14	Limit S13 to studies published from September 2014 to Current	1158

**January 2000‐ CURRENT (February 2015): Providers Searches**

**Table:**

#	Searches	No of Hits
S1	(MM "Prenatal Care")	4215
S2	(MM “Perinatal Care”)	1301
S3	((MM "Perinatal Care")) OR (S1)	5474
S4	(MM “Maternal Health Services+)	9017
S5	(MM "Maternal Health Services")) OR (S3)	10218
S6	TI antenatal care or AB antenatal care or TI prenatal care or AB prenatal care or TI pregnan$ or AB pregnan$ or TI perinatal care or AB perinatal care	4711
S7	(TI antenatal care or AB antenatal care or TI prenatal care or AB prenatal care or TI pregnan$ or AB pregnan$ or TI perinatal care or AB perinatal care) OR (S5	12942
S8	Staff or provider$ or health care provider$ or nurs$ or midwife$ or midwives or physician$ or doctor$ or obstet$ or medical professional$ or clinician$ or skilled birth attendant$ or auxiliary or lay health worker$ or community health worker$ or traditional birth attendant$ or tba$	311, 745
S9	"healthcare personnel" OR (MM "Secondary Health Care") OR (MM "Health Personnel+") OR (MM "Rural Health Personnel")	176,957
S10	("healthcare personnel" OR (MM "Secondary Health Care") OR (MM "Health Personnel+") OR (MM "Rural Health Personnel")) OR (S8)	425,304
S11	(("healthcare personnel" OR (MM "Secondary Health Care") OR (MM "Health Personnel+") OR (MM "Rural Health Personnel")) OR (S8)) AND (S7)	3623
S12	(MM “Qualitative Studies+”)	4028
S13	TI qualitative or AB qualitative or TI focus group$ or AB focus group$ or TI interview$ or AB interview$	108,225
S14	(TI qualitative or AB qualitative or TI focus group$ or AB focus group$ or TI interview$ or AB interview$) OR (S12)	115, 851
S15	((TI qualitative or AB qualitative or TI focus group$ or AB focus group$ or TI interview$ or AB interview$) OR (S12)) AND (S11)	1381
S14	Limit S15 to studies published from January 2000 to Current	738

**February 2015 – CURRENT (February 2019): Providers Searches**

**Table:**

#	Searches	No of Hits
S1	(MM "Prenatal Care")	8072
S2	(MM “Perinatal Care”)	2420
S3	((MM "Perinatal Care")) OR (S1)	10,409
S4	(MM “Maternal Health Services+)	16,284
S5	(MM "Maternal Health Services")) OR (S3)	18,534
S6	TI antenatal care or AB antenatal care or TI prenatal care or AB prenatal care or TI pregnan$ or AB pregnan$ or TI perinatal care or AB perinatal care	9593
S7	(TI antenatal care or AB antenatal care or TI prenatal care or AB prenatal care or TI pregnan$ or AB pregnan$ or TI perinatal care or AB perinatal care) OR (S5	24, 075
S8	Staff or provider$ or health care provider$ or nurs$ or midwife$ or midwives or physician$ or doctor$ or obstet$ or medical professional$ or clinician$ or skilled birth attendant$ or auxiliary or lay health worker$ or community health worker$ or traditional birth attendant$ or tba$	528,548
S9	"healthcare personnel" OR (MM "Secondary Health Care") OR (MM "Health Personnel+") OR (MM "Rural Health Personnel")	298,688
S10	("healthcare personnel" OR (MM "Secondary Health Care") OR (MM "Health Personnel+") OR (MM "Rural Health Personnel")) OR (S8)	720,851
S11	(("healthcare personnel" OR (MM "Secondary Health Care") OR (MM "Health Personnel+") OR (MM "Rural Health Personnel")) OR (S8)) AND (S7)	6793
S12	(MM “Qualitative Studies+”)	6848
S13	TI qualitative or AB qualitative or TI focus group$ or AB focus group$ or TI interview$ or AB interview$	206,580
S14	(TI qualitative or AB qualitative or TI focus group$ or AB focus group$ or TI interview$ or AB interview$) OR (S12)	209,970
S15	((TI qualitative or AB qualitative or TI focus group$ or AB focus group$ or TI interview$ or AB interview$) OR (S12)) AND (S11)	1513
S14	Limit S15 to studies published from February 2015 to Current	734

**PsycINFO EbscoHost;**

**January 2000 – CURRENT (September 2014): Women’s Searches**

**Table:**

#	Searches	No of Hits
S1	((MM "Prenatal Care" OR MM "Childbirth Training") OR (MM "Antepartum Period")) OR (MM "Pregnancy" OR MM "Adolescent Pregnancy" OR MM "Pregnancy Outcomes" OR MM "Primipara")	10,957
S2	TI antenatal care or AB antenatal care or TI prenatal care or AB prenatal care or TI pregnan$ or AB pregnan$ or TI perinatal care or AB perinatal care	2130
S3	(TI antenatal care or AB antenatal care or TI prenatal care or AB prenatal care or TI pregnan$ or AB pregnan$ or TI perinatal care or AB perinatal care) OR (S1)	11,930
S4	TI woman$ or AB woman$ or TI women$ or AB women$ or TI mother$ or AB mother$	189,386
S5	(TI woman$ or AB woman$ or TI women$ or AB women$ or TI mother$ or AB mother$) AND (S3)	9146
S6	((MM "Qualitative Methods" OR MM "Focus Group" OR MM "Grounded Theory" OR MM "Interpretative Phenomenological Analysis" OR MM "Narrative Analysis" OR MM "Semi‐Structured Interview" OR MM "Thematic Analysis") OR (MM "Mixed Methods Research")) OR (MM "Participant Observation")	5413
S7	TI qualitative or AB qualitative or TI focus group$ or AB focus group$ or TI interview$ or AB interview$	192,056
S8	TI qualitative or AB qualitative or TI focus group$ or AB focus group$ or TI interview$ or AB interview$ OR (S6)	192,837
S9	((TI qualitative or AB qualitative or TI focus group$ or AB focus group$ or TI interview$ or AB interview$) OR (S6)) AND (S5)	2125
S10	Limit S9 to studies published from January 2000 to Current	1775

**September 2014 – CURRENT (February 2019): Women’s Searches**

**Table:**

#	Searches	No of Hits
S1	((MM "Prenatal Care" OR MM "Childbirth Training") OR (MM "Antepartum Period")) OR (MM "Pregnancy" OR MM "Adolescent Pregnancy" OR MM "Pregnancy Outcomes" OR MM "Primipara")	20672
S2	TI antenatal care or AB antenatal care or TI prenatal care or AB prenatal care or TI pregnan$ or AB pregnan$ or TI perinatal care or AB perinatal care	3671
S3	(TI antenatal care or AB antenatal care or TI prenatal care or AB prenatal care or TI pregnan$ or AB pregnan$ or TI perinatal care or AB perinatal care) OR (S1)	22385
S4	TI woman$ or AB woman$ or TI women$ or AB women$ or TI mother$ or AB mother$	381,148
S5	(TI woman$ or AB woman$ or TI women$ or AB women$ or TI mother$ or AB mother$) AND (S3)	16088
S6	((MM "Qualitative Methods" OR MM "Focus Group" OR MM "Grounded Theory" OR MM "Interpretative Phenomenological Analysis" OR MM "Narrative Analysis" OR MM "Semi‐Structured Interview" OR MM "Thematic Analysis") OR (MM "Mixed Methods Research")) OR (MM "Participant Observation")	8842
S7	TI qualitative or AB qualitative or TI focus group$ or AB focus group$ or TI interview$ or AB interview$	354,042
S8	TI qualitative or AB qualitative or TI focus group$ or AB focus group$ or TI interview$ or AB interview$ OR (S6)	355563
S9	((TI qualitative or AB qualitative or TI focus group$ or AB focus group$ or TI interview$ or AB interview$) OR (S6)) AND (S5)	2918
S10	Limit S9 to studies published from September 2014 to Current	788

**January 2000 – CURRENT (February 2015): Providers Searches**

**Table:**

#	Searches	No of Hits
S1	((MM "Prenatal Care" OR MM "Childbirth Training") OR (MM "Antepartum Period")) OR (MM "Pregnancy" OR MM "Adolescent Pregnancy" OR MM "Pregnancy Outcomes" OR MM "Primipara")	11, 525
S2	TI antenatal care or AB antenatal care or TI prenatal care or AB prenatal care or TI pregnan$ or AB pregnan$ or TI perinatal care or AB perinatal care	2236
S3	(TI antenatal care or AB antenatal care or TI prenatal care or AB prenatal care or TI pregnan$ or AB pregnan$ or TI perinatal care or AB perinatal care) OR (S1)	12,546
S4	Staff or provider$ or health care provider$ or nurs$ or midwife$ or midwives or physician$ or doctor$ or obstet$ or medical professional$ or clinician$ or skilled birth attendant$ or auxiliary or lay health worker$ or community health worker$ or traditional birth attendant$ or tba$	181,588
S5	((MM "Health Personnel" OR MM "Professional Personnel" OR MM "Allied Health Personnel" OR MM "Caregivers") OR (MM "Midwifery")) OR (MM "Obstetricians")	22,446
S6	(((MM "Health Personnel" OR MM "Professional Personnel" OR MM "Allied Health Personnel" OR MM "Caregivers") OR (MM "Midwifery")) OR (MM "Obstetricians")) OR (S4)	196,819
S7	((((MM "Health Personnel" OR MM "Professional Personnel" OR MM "Allied Health Personnel" OR MM "Caregivers") OR (MM "Midwifery")) OR (MM "Obstetricians")) OR (S4)) AND (S3)	2341
S8	((MM "Qualitative Methods" OR MM "Focus Group" OR MM "Grounded Theory" OR MM "Interpretative Phenomenological Analysis" OR MM "Narrative Analysis" OR MM "Semi‐Structured Interview" OR MM "Thematic Analysis") OR (MM "Mixed Methods Research")) OR (MM "Participant Observation")	6288
S9	TI qualitative or AB qualitative or TI focus group$ or AB focus group$ or TI interview$ or AB interview$	204,394
S10	TI qualitative or AB qualitative or TI focus group$ or AB focus group$ or TI interview$ or AB interview$ OR (S8)	205,369
S11	((TI qualitative or AB qualitative or TI focus group$ or AB focus group$ or TI interview$ or AB interview$) OR (S8)) AND (S7)	857
S12	Limit S11 to studies published from January 2000 to Current	601

**Feb 2015 – CURRENT (February 2019): Providers Searches**

**Table:**

#	Searches	No of Hits
S1	((MM "Prenatal Care" OR MM "Childbirth Training") OR (MM "Antepartum Period")) OR (MM "Pregnancy" OR MM "Adolescent Pregnancy" OR MM "Pregnancy Outcomes" OR MM "Primipara")	20,676
S2	TI antenatal care or AB antenatal care or TI prenatal care or AB prenatal care or TI pregnan$ or AB pregnan$ or TI perinatal care or AB perinatal care	3673
S3	(TI antenatal care or AB antenatal care or TI prenatal care or AB prenatal care or TI pregnan$ or AB pregnan$ or TI perinatal care or AB perinatal care) OR (S1)	22,390
S4	Staff or provider$ or health care provider$ or nurs$ or midwife$ or midwives or physician$ or doctor$ or obstet$ or medical professional$ or clinician$ or skilled birth attendant$ or auxiliary or lay health worker$ or community health worker$ or traditional birth attendant$ or tba$	317,176
S5	((MM "Health Personnel" OR MM "Professional Personnel" OR MM "Allied Health Personnel" OR MM "Caregivers") OR (MM "Midwifery")) OR (MM "Obstetricians")	35,218
S6	(((MM "Health Personnel" OR MM "Professional Personnel" OR MM "Allied Health Personnel" OR MM "Caregivers") OR (MM "Midwifery")) OR (MM "Obstetricians")) OR (S4)	341,921
S7	((((MM "Health Personnel" OR MM "Professional Personnel" OR MM "Allied Health Personnel" OR MM "Caregivers") OR (MM "Midwifery")) OR (MM "Obstetricians")) OR (S4)) AND (S3)	3628
S8	((MM "Qualitative Methods" OR MM "Focus Group" OR MM "Grounded Theory" OR MM "Interpretative Phenomenological Analysis" OR MM "Narrative Analysis" OR MM "Semi‐Structured Interview" OR MM "Thematic Analysis") OR (MM "Mixed Methods Research")) OR (MM "Participant Observation")	8844
S9	TI qualitative or AB qualitative or TI focus group$ or AB focus group$ or TI interview$ or AB interview$	354,176
S10	TI qualitative or AB qualitative or TI focus group$ or AB focus group$ or TI interview$ or AB interview$ OR (S8)	355,687
S11	((TI qualitative or AB qualitative or TI focus group$ or AB focus group$ or TI interview$ or AB interview$) OR (S8)) AND (S7)	912
S12	Limit S11 to studies published from February 2015 to Current	261

**AMED EbscoHost;**

**January 2000 – CURRENT (September 2014): Women’s Searches**

**Table:**

#	Searches	No of Hits
S1	antenatal or prenatal or antepartum or prepregnancy or pregnant or pregnancy or perinatal or maternal health	1257
S2	care or service or services or provider or providers or provision or service or services or delivery or support	47388
S3	(care or service or services or provider or providers or provision or service or services or delivery or support) AND (S1	446
S4	qualitative or interview or interviews or focus group or focus groups	8945
S5	(qualitative or interview or interviews or focus group or focus groups) AND (S3)	76
S6	Limit (S5) to studies published between Jan 2000 – current	50

**September 2014 – CURRENT (February 2019): Women’s Searches**

**Table:**

#	Searches	No of Hits
S1	antenatal or prenatal or antepartum or prepregnancy or pregnant or pregnancy or perinatal or maternal health	2429
S2	care or service or services or provider or providers or provision or service or services or delivery or support	79627
S3	(care or service or services or provider or providers or provision or service or services or delivery or support) AND (S1	865
S4	qualitative or interview or interviews or focus group or focus groups	12462
S5	(qualitative or interview or interviews or focus group or focus groups) AND (S3)	80
S6	Limit (S5) to studies published between September 2014 – current	30

**January 2000 – CURRENT (February 2015): Providers Searches**

**Table:**

#	Searches	No of Hits
S1	antenatal or prenatal or antepartum or prepregnancy or pregnant or pregnancy or perinatal or maternal health	1370
S2	care or service or services or provider or providers or provision or service or services or delivery or support	51,876
S3	(care or service or services or provider or providers or provision or service or services or delivery or support) AND (S1	501
S4	qualitative or interview or interviews or focus group or focus groups	9370
S5	(qualitative or interview or interviews or focus group or focus groups) AND (S3)	78
S6	Limit (S5) to studies published between Jan 2000 – current	52

**Febraury 2015 – CURRENT (February 2019): Providers Searches**

**Table:**

#	Searches	No of Hits
S1	antenatal or prenatal or antepartum or prepregnancy or pregnant or pregnancy or perinatal or maternal health	2429
S2	care or service or services or provider or providers or provision or service or services or delivery or support	79627
S3	(care or service or services or provider or providers or provision or service or services or delivery or support) AND (S1	865
S4	qualitative or interview or interviews or focus group or focus groups	12462
S5	(qualitative or interview or interviews or focus group or focus groups) AND (S3)	80
S6	Limit (S5) to studies published between February 2015 – current	30

**LILACS Virtual Health Library;**

**January 2000 – CURRENT (September 2014): Women’s Searches**

**Table:**

#	Searches	No of Hits
S1	(tw:(mh:("prenatal care") OR mh:("maternal health services") OR mh:("maternal health") OR mh:("perinatal care") )) AND (tw:(mh:("pregnant women") OR woman* OR women* OR mother* OR mum* )) AND (tw:(qualitative))	1798
S2	Limit S1 to db:("LILACS")	103
S3	Limit S2 to studies published between January 2000‐ current	102

**September 2014 – CURRENT (February 2019): Women’s Searches**

**Table:**

#	Searches	No of Hits
S1	(tw:(mh:("prenatal care") OR mh:("maternal health services") OR mh:("maternal health") OR mh:("perinatal care") )) AND (tw:(mh:("pregnant women") OR woman* OR women* OR mother* OR mum* )) AND (tw:(qualitative))	2220
S2	Limit S1 to db:("LILACS")	151
S3	Limit S2 to studies published between February 2014‐ current	48

**January 2000 – CURRENT (February 2015): Providers Searches**

**Table:**

#	Searches	No of Hits
S1	(tw:(mh:("prenatal care") OR mh:("maternal health services") OR mh:("maternal health") OR mh:("perinatal care") )) AND (tw:(mh:("health personnel") OR staff OR provider* OR health care provider* OR nurs* OR midwife OR midwives OR physician* OR doctor* OR obstet* OR medical professional* OR clinician* OR skilled birth attendant* OR auxiliary OR lay health worker* OR community health worker* OR traditional birth attendant* OR tba*)) AND (tw:(qualitative))	519
S2	Limit S1 to db:("LILACS")	26
S3	Limit S2 to studies published between January 2000‐ current	24

**February 2015 – CURRENT (February 2019): Providers Searches**

**Table:**

#	Searches	No of Hits
S1	(tw:(mh:("prenatal care") OR mh:("maternal health services") OR mh:("maternal health") OR mh:("perinatal care") )) AND (tw:(mh:("health personnel") OR staff OR provider* OR health care provider* OR nurs* OR midwife OR midwives OR physician* OR doctor* OR obstet* OR medical professional* OR clinician* OR skilled birth attendant* OR auxiliary OR lay health worker* OR community health worker* OR traditional birth attendant* OR tba*)) AND (tw:(qualitative))	1072
S2	Limit S1 to db:("LILACS")	36
S3	Limit S2 to studies published between February 2015‐ current	10

**AJOL (African Journals Online);**

**January 2000 – CURRENT (February 2015): General Searches**

Care (antenatal or prenatal) ‐ 27

**February 2015 – CURRENT (February 2019): General Searches**

Care (antenatal or prenatal) ‐ 70

**January 2000 – CURRENT (February 2015): General Searches**

Care (antenatal or prenatal) 34

**February 2015 – CURRENT (February 2019): General Searches**

Care (antenatal or prenatal) 70

## Characteristics of studies

### Characteristics of included studies [ordered by study ID]

**Table:** Abrahams 2001

Country	South Africa	
Resource	Upper middle	
Context	Women recruited from 2 primary ANC clinics and 2 secondary hospitals providing ANC in a peri‐urban setting in Cape Town, South Africa	
Notes	Quality rating: C+

**Table:** Agus 2012

Country	Indonesia	
Resource	Lower middle	
Context	Study conducted in a village location in a relatively poor area of West Java	
Notes	Quality rating: B

**Table:** Alderson 2004

Country	UK	
Resource	High	
Context	A teaching hospital, and a district general hospital in urban areas of the UK	
Notes	Qualilty rating: B

**Table:** Andrew 2014

Country	Papua New Guinea PNG)	
Resource	Lower middle	
Context	Community‐based study in rural areas surrounding 2 health centres and 1 district hospital in northern PNG	
Notes	Quality rating: Women: A Health professionals: B+

**Table:** Armstrong 2005

Country	Australia	
Resource	High	
Context	An urban health facility in Australia	
Notes	Quality rating: B+

**Table:** Ayala 2013

Country	Peru	
Resource	Upper middle	
Context	Study conducted at a regional hospital in an Andean province with a large indigenous population	
Notes	Quality rating: A−

**Table:** Ayiasi 2013

Country	Uganda	
Resource	Low	
Context	2 rural districts in northern Uganda including 2 hospitals, 1 regional health centre and 21 smaller health facilities	
Notes	Quality rating Women: B Health professionals: B

**Table:** Baffour‐Awuah 2015

Country	Ghana	
Resource	Lower middle	
Context	Study conducted in an outpatient ANC unit at a large urban hospital in Tema, Ghana	
Notes	Quality rating: B+

**Table:** Bessett 2010

Country	USA	
Resource	High	
Context	Women experiencing a range of ANC services (public and private) in metropolitan New York	
Notes	Quality rating: B−

**Table:** Biondi 2018

Country	Brazil	
Resource	Upper middle	
Context	Obstetrics and maternity centres in 2 metropolitan hospitals in southern Brazil	
Notes	Quality rating: C+

**Table:** Bradley 2012

Country	Ethiopia	
Resource	Low	
Context	7 primary health care units (PHCUs) selected by performance indicators as part of a longitudinal study of 20 PHCUs	
Notes	Quality rating: A

**Table:** Cabral 2013

Country	Brazil	
Resource	Upper middle	
Context	4 different types of health facility (private and public) in an urban city in Brazil	
Notes	Quality rating: C+

**Table:** Cardelli 2016

Country	Brazil	
Resource	Upper middle	
Context	Several basic health units in a municipality in Southern Brazil	
Notes	C+

**Table:** Chapman 2003

Country	Mozambique	
Resource	Low	
Context	Community‐based study in a rural area of Mozambique with high levels of poverty and deprivation and very low levels of ANC access	
Notes	Quality rating: A

**Table:** Chimezie 2013

Country	Nigeria	
Resource	Lower middle	
Context	A range of primary and community health centres in a rural, economically disadvantaged area of Nigeria	
Notes	Quality rating: A

**Table:** Choudhury 2011

Country	Bangladesh	
Resource	Low	
Context	Set in 2 relatively deprived communities (urban/slum and rural) in northern Bangladesh	
Notes	Quality rating: B−

**Table:** Chowdhury 2003

Country	Bangladesh	
Resource	Low	
Context	Set in 2 communities: a slum area of Dhaka city and a rural village with few modern amenities, as part of a larger study looking at skilled birth attendance	
Notes	Quality rating: B−

**Table:** Conrad 2012

Country	Uganda	
Resource	Low	
Context	4 health facilities (a district hospital, 3 clinics) purposively selected to represent the different levels of care available in the study area	
Notes	Quality rating Women: A− Health professionals: C+

**Table:** Coverston 2004

Country	Argentina	
Resource	Upper middle	
Context	A large public maternity hospital in Northwest Argentina with high levels of deprivation and poor ANC access	
Notes	Quality rating: B

**Table:** Dako‐Gyeke 2013

Country	Ghana	
Resource	Lower middle	
Context	Health facilities in 4 districts of peri‐urban Accra, Ghana	
Notes	Quality rating Women: B+ Health professionals: C+

**Table:** De Castro 2010

Country	Brazil	
Resource	Upper middle	
Context	Study conducted in municipal health units in urban Belem, Brazil	
Notes	Quality rating: B

**Table:** Docherty 2011

Country	UK	
Resource	High	
Context	Set in a large maternity unit in west central Scotland with extremes of social and economic deprivation	
Notes	Quality rating: B

**Table:** Duarte 2012

Country	Brazil	
Resource	Upper middle	
Context	An urban health facility in Brazil	
Notes	Quality rating: C+

**Table:** Earle 2000

Country	UK	
Resource	High	
Context	Based on recruitment from 12 ANC clinics in the West Midlands with a relatively diverse population	
Notes	Quality rating: B+

**Table:** Family Care International 2003

Country	Kenya	
Resource	Low	
Context	Set in 2 semi‐rural districts of Western Kenya with relatively high levels of poverty and deprivation	
Notes	Quality rating: B+

**Table:** Franngard 2006

Country	Uganda	
Resource	Low	
Context	9 health centres and 1 hospital in rural Uganda	
Notes	Quality rating: B+

**Table:** Ganle 2014

Country	Ghana	
Resource	Lower middle	
Context	6 purposively sampled communities in rural and urban districts of the northern region of Ghana	
Notes	Quality rating: A−

**Table:** Gheibizadeh 2016

Country	Iran	
Resource	Upper middle	
Context	6 urban health centres across Ahvaz, a city in south‐western Iran	
Notes	Quality rating: A−

**Table:** Graner 2010

Country	Vietnam	
Resource	Lower middle	
Context	32 communal health stations in a rural area of northern Vietnam	
Notes	Quality rating: B+

**Table:** Graner 2013

Country	Vietnam	
Resource	Lower middle	
Context	Based on recruitment from 32 communal health posts in a relatively deprived area of rural Vietnam	
Notes	Quality rating: B

**Table:** Griffiths 2001

Country	India	
Resource	Lower middle	
Context	Set in the urban and surrounding rural areas of 2 large Indian cities (Pune and Mumbai)	
Notes	Quality rating: B

**Table:** Gross 2011

Country	Tanzania	
Resource	Low	
Context	4 antenatal clinics in a rural area of south‐eastern Tanzania	
Notes	Quality rating: B

**Table:** Haddrill 2014

Country	UK	
Resource	High	
Context	Women recruited from a maternity unit in an urban centre in northern England	
Notes	Quality rating: A−

**Table:** Heaman 2015

Country	Canada	
Resource	High	
Context	Study conducted across 8 metropolitan districts in inner city Winnipeg, Canada	
Notes	B

**Table:** Heberlein 2016

Country	USA	
Resource	High	
Context	Large hospital‐affiliated obstetrics practice in South Carolina providing ANC to a racially diverse population	
Notes	Quality rating: A−

**Table:** Hunter 2017

Country	Republic of Ireland	
Resource	High	
Context	Large urban maternity hospital and a smaller regional hospital in different parts of Ireland	
Notes	Quality rating: A

**Table:** Kabakian‐Khasholian 2000

Country	Lebanon	
Resource	Upper middle	
Context	Women recruited from 1 urban (Beruit), 1 semi‐rural and 2 remote rural locations in Lebanon	
Notes	Quality rating: B−

**Table:** Khoso 2016

Country	Pakistan	
Resource	Lower middle	
Context	Women recruited from 3 districts of Baluchistan (Gwadar, Qila Saifullah and Quetta) in semi‐rural Pakistan	
Notes	Quality rating: C

**Table:** Kraschnewski 2014

Country	USA	
Resource	High	
Context	Pregnant women owning a smartphone recruited from a health clinic in central Pennsylvania	
Notes	Quality rating: B

**Table:** Lagan 2011

Country	5 high‐income countries (Australia, New Zealand, Canada, USA and UK)	
Resource	High	
Context	Study used online forums to recruit women into an Internet‐based discussion	
Notes	Quality rating: A−

**Table:** Larsen 2004

Country	Papua New Guinea (PNG)	
Resource	Lower middle	
Context	3 health facilities in the urban, peri‐urban and rural districts of Goroka, PNG	
Notes	Quality rating: C+

**Table:** Larsson 2017

Country	Sweden	
Resource	High	
Context	Study conducted in 3 maternity clinics in different parts of Sweden	
Notes	Quality rating: A

**Table:** Lasso Toro 2012

Country	Colombia	
Resource	Upper middle	
Context	Health facilities in a largely rural area of Colombia with high levels of socio‐economic deprivation	
Notes	Quality rating: C+

**Table:** Leal 2018

Country	Brazil	
Resource	Upper middle	
Context	Various facilities providing ANC in a relatively rural area in north‐east Brazil	
Notes	Quality rating: C

**Table:** LeMasters 2018

Country	Romania	
Resource	Upper middle	
Context	Diverse settings including health centres across Romania as well as rural villages and a Roma community	
Notes	Quality rating: A

**Table:** Mahiti 2015

Country	Tanzania	
Resource	Low	
Context	Women recruited from a district hospital and a health centre in rural Tanzania	
Notes	Quality rating: B+

**Table:** Manithip 2013

Country	Laos	
Resource	Lower middle	
Context	4 district hospitals and 18 health centres in rural areas of Laos	
Notes	Quality rating: B+

**Table:** Maputle 2013

Country	South Africa	
Resource	Upper middle	
Context	Women recruited from a village location in rural South Africa	
Notes	Quality rating: A

**Table:** Mathole 2005

Country	Zimbabwe	
Resource	Low	
Context	18 rural health centres in south‐eastern Zimbabwe	
Notes	Quality rating: B+

**Table:** Matsuoka 2010

Country	Cambodia	
Resource	Low	
Context	Study conducted in 6 purposively selected communities in 3 districts of rural Cambodia	
Notes	Quality rating: B−

**Table:** Mayca 2009

Country	Peru	
Resource	Upper middle	
Context	3 districts of rural Peru with high rates of poverty and a large % of indigenous communities	
Notes	Quality rating Women: B− Health professionals: B−

**Table:** McDonald 2014

Country	Canada	
Resource	High	
Context	An ANC clinic offering group prenatal care in urban Ontario	
Notes	Quality rating Women: B− Health professionals: B

**Table:** McNeil 2012

Country	Canada	
Resource	High	
Context	Study conducted in an urban maternity clinic in Calgary, Canada providing group ANC	
Notes	Quality rating: B+

**Table:** Miteniece 2018

Country	Georgia	
Resource	Lower middle	
Context	Study conducted in 2 urban settings (Tbilisi and Kutaisi) and 1 rural setting (Batumi area)	
Notes	Quality rating: A

**Table:** Molina 2011

Country	Colombia	
Resource	Upper middle	
Context	18 public and private Healthcare Provider Institutions affiliated with different Healthcare Promotion Insurance Companies in urban Medellin	
Notes	Quality rating: B−

**Table:** Mrisho 2009

Country	Tanzania	
Resource	Low	
Context	Health facilities in 8 villages from 2 districts in rural Tanzania	
Notes	Quality rating Women: B Health professionals: B

**Table:** Mugo 2018

Country	South Sudan	
Resource	Low	
Context	3 public health facilities in Juba, South Sudan	
Notes	Quality rating: B

**Table:** Mumtaz 2007

Country	Pakistan	
Resource	Lower middle	
Context	Study conducted in a small rural village in northern Pakistan	
Notes	Quality rating: B+

**Table:** Munguambe 2016

Country	Mozambique	
Resource	Low	
Context	Part of a larger study conducted in several diverse districts (urban and rural) in eastern Mozambique	
Notes	B−

**Table:** Myer 2003

Country	South Africa	
Resource	Upper middle	
Context	Set in a rural district of South Africa with relatively high rates of maternal and infant mortality	
Notes	Quality rating: C+

**Table:** Neves 2013

Country	Brazil	
Resource	Upper middle	
Context	A health centre in urban Sao Paolo providing group ANC	
Notes	Quality rating: B

**Table:** Novick 2011

Country	USA	
Resource	High	
Context	Study conducted at 2 urban clinics in 2 north‐eastern states, 1 in a hospital and 1 in a freestanding community health centre in a low‐income suburb. Both provided group ANC	
Notes	Quality rating: A−

**Table:** Novick 2013

Country	USA	
Resource	High	
Context	2 urban clinics providing care to low‐income women in the north‐eastern USA	
Notes	Quality rating: A−

**Table:** Pretorius 2004

Country	South Africa	
Resource	Upper middle	
Context	Conducted in a variety of healthcare facilities in a rural area of South Africa with relatively low ANC usage	
Notes	Quality rating: B

**Table:** Rahmani 2013

Country	Afghanistan	
Resource	Low	
Context	1 maternity hospital, 2 clinics (1 private, 1 NGO‐run) in urban Kabul and 1 village health facility in rural Afghanistan	
Notes	Quality rating Women: C+ Health professionals: B

**Table:** Rath 2010

Country	India	
Resource	Lower middle	
Context	Community‐based intervention study (using women’s groups) conducted in a rural area of eastern India including 193 villages and 254 hamlets	
Notes	Quality rating: A−

**Table:** Saftner 2017

Country	USA	
Resource	High	
Context	Various institutions across Minnesota including large urban hospitals, urban community hospitals, and 2 smaller cities that cared for women from local and rural areas	
Notes	B+

**Table:** Santos 2010

Country	Brazil	
Resource	Upper middle	
Context	Study set in a public health clinic in Cajazeiras‐PB city, Brazil	
Notes	Quality rating: C+

**Table:** Shabila 2014

Country	Iraq	
Resource	Upper middle	
Context	Women recruited from diverse socio‐economic communities in Erbil, northern Iraq	
Notes	Quality rating: B+

**Table:** Simkhada 2010

Country	Nepal	
Resource	Low	
Context	Rural and urban communities in Nepal	
Notes	Quality rating: C+

**Table:** Spindola 2012

Country	Brazil	
Resource	Upper middle	
Context	Study set in an urban hospital in Rio de Janeiro, Brazil	
Notes	Quality rating: C+

**Table:** Stokes 2008

Country	Gambia	
Resource	Low	
Context	Community‐based study conducted in 3 rural villages in western Gambia purposively chosen based on relative distance from a health facility (1 near, 1 mid‐distance, 1 far)	
Notes	Quality rating: B−

**Table:** Sword 2003

Country	Canada	
Resource	High	
Context	Women recruited from 2 low‐income communities in Ontario, Canada	
Notes	Quality rating: A

**Table:** Sword 2012

Country	Canada	
Resource	High	
Context	5 urban centres across Canada	
Notes	Quality rating: Women: A− Health professionals: A−

**Table:** Sychareun 2016

Country	Laos	
Resource	Lower middle	
Context	4 rural districts in 2 provinces in central and south‐eastern Laos; chosen for their ethnic diversity	
Notes	Quality rating: B−

**Table:** Teate 2011

Country	Australia	
Resource	High	
Context	2 metropolitan hospitals in Sydney, Australia providing group ANC	
Notes	Quality rating: C+

**Table:** Teate 2013

Country	Australia	
Resource	High	
Context	2 hospital antenatal clinics and 2 community healthcare centres in urban southern Sydney	
Notes	Quality rating: A

**Table:** Thwala 2011

Country	Swaziland	
Resource	Low	
Context	Part of a larger study exploring the childbirth practices of a village community in northern Swaziland	
Notes	Quality rating: A

**Table:** Titaley 2010

Country	Indonesia	
Resource	Lower middle	
Context	Health facilities in 6 villages in 3 districts of west Java (3 urban and 3 rural)	
Notes	Quality rating Women: C+ Health professionals: C+

**Table:** Umeora 2008

Country	Nigeria	
Resource	Lower middle	
Context	Study conducted in a rural mission hospital providing ANC in Nigeria	
Notes	Quality rating: B−

**Table:** Walburg 2014

Country	France	
Resource	High	
Context	A large hospital maternity unit in Toulouse, France	
Notes	B−

**Table:** Wilmore 2015

Country	Australia	
Resource	High	
Context	Study framed around exploring midwife communication practices at a large metropolitan hospital in South Australia	
Notes	Quality rating: B−

**Table:** Worley 2004

Country	New Zealand	
Resource	High	
Context	Set in a public prenatal clinic located in an urban city in New Zealand	
Notes	Quality rating: B

**Table:** Wright 2018

Country	Australia	
Resource	High	
Context	Focused ethnography conducted in 6 publicly funded ANC clinics in South Australia	
Notes	Quality rating: A

**Table:** Østergaard 2015

Country	Burkina Faso	
Resource	Low	
Context	3 primary healthcare centres (2 urban, 1 rural) in the central‐east region of Burkina Faso	
Notes	A−	

ANC: antenatal care; FGD: focus group discussion; HIC: high‐income country; NGO: non‐governmental organisation; PHCU: primary health care unit; TBA: traditional birth assistant

### Characteristics of excluded studies [ordered by study ID]

**Table:**

Study	Reason for exclusion
Lohmann 2018	Study framed around midwives discussing what women may think about antenatal care (one of our exclusion criteria)
Murira 2003	Study focused on communication between women and healthcare providers rather than views and experiences of antenatal care
Nigenda 2003	Insufficient quote material from participants to support study findings
Pell 2013	Insufficient quote material from participants to support study findings
Påfs 2015	Study framed around 'near miss' episodes during pregnancy
Tsawe 2014	Insufficient quote material from participants to support study findings

### Characteristics of studies awaiting assessment [ordered by study ID]

**Table:** Aikawa 2004

Notes	Article is in Japanese ‐ unable to translate

## Differences between protocol and review

This review is based on two protocols (Downe 2016b; Downe 2017) that have been merged for the final analysis. This allowed for 'compare and contrast' decisions to be made. It also allowed for an analysis of maternity care as a complex adaptive system, in which the views and experiences of service users and providers are both integral, and in which service delivery and service uptake interact dynamically. We combined the search strategies and inclusion criteria into one overarching search process. Data analysis and all other aspects of the reviews were the same in both protocols, and, apart from the sampling and sub‐analyses planned in the protocols, we followed these processes for the final review.

In the protocol for this review under the heading 'Types of Interventions' we used a broader definition of antenatal care that included the phrase, "helping women to prepare for birth and parenting". When we conducted the initial searches it became clear that this phrase would pick up studies relating to formal antenatal education sessions. As our review was focused on routine antenatal care rather than specific educational sessions offered during the antenatal period we removed the phrase from our definition in this review.

In the protocol for this review, we planned to undertake sampling if there were more than 30 eligible studies. In the event, we had more than 30 studies, but we considered that sampling was only necessary for the more recent studies (located in our final updated search), to ensure full diversity in study inclusion, and adequate data richness, We have therefore amended our Methods section in the final version of the review, to reflect this decision.

In our protocol we stated that the logic models we planned to use were based on the reasoned action approach (Fishbein 2010). In fact, the model provided in Figure 1 in the protocol was for the theory of planned behaviour (Azjen 1991), and this is the theory we used for the analysis in this review. We have amended the label for the figure for accuracy.

## References

[CD012392-bib-0001] Abrahams N, Jewkes R, Mvo Z. Health care‐seeking practices of pregnant women and the role of the midwife in Cape Town, South Africa. Journal of Midwifery and Women’s Health 2001;46(40):240‐7. 10.1016/s1526-9523(01)00138-6 11603639

[CD012392-bib-0002] Agus Y, Horiuchi S, Porter SE. Rural Indonesia women’s traditional beliefs about antenatal care. BMC Research Notes 2012;5:589. [www.biomedcentral.com/1756‐0500/5/589.] 10.1186/1756-0500-5-589 PMC3532090 23106915

[CD012392-bib-0003] Alderson P, Williams C, Farsides B. Pracitioners’ views about equity within prenatal services. Sociology 2004;38(1):61‐80.

[CD012392-bib-0004] Andrew EV, Pell C, Angwin A, Auwun A, Daniels J, Mueller I, et al. Factors affecting attendance at and timing of formal antenatal care: results from a qualitative study in Madang, Papua New Guinea. PLOS One 2014;9(5):e93025. [DOI: 10.1371/journal.pone.0093025] PMC4026245 24842484

[CD012392-bib-0005] Armstrong TM, Pooley JA. Being pregnant: a qualitative study of women's lived experience of being pregnant. Journal of Prenatal and Perinatal Psychology and Health 2005;20(1):4‐25.

[CD012392-bib-0006] Ayala LS, Blumenthal PD, Sarnquist CC. Factors influencing women’s decision to seek antenatal care in the Andes of Peru. Maternal and Child Health Journal 2013;17(6):1112‐18. [DOI: 10.1007/s10995-012-1113-9] 22956365

[CD012392-bib-0007] Ayiasi MR, Royen K Van, Verstraeten R, Atuyambe L, Criel B, Garimoi CO, et al. Exploring the focus of prenatal information offered to pregnant mothers regarding newborn care in rural Uganda. BMC Pregnancy and Childbirth 2013;13:176. [www.biomedcentral.com/1471‐2393/13/176] 10.1186/1471-2393-13-176 PMC3848633 24041135

[CD012392-bib-0008] Baffour‐Awuah A, Mwini‐Nyaledzigbor PP, Richter S. Enhancing focused antenatal care in Ghana: an exploration into perceptions of practicing midwives. International Journal of Africa Nursing Sciences 2015;2:59‐64.

[CD012392-bib-0009] Bessett D. Negotiating normalization: the perils of producing pregnancy symptoms in prenatal care. Social Science & Medicine 2010;71(2):370‐7. 10.1016/j.socscimed.2010.04.007 20494503

[CD012392-bib-0010] Biondi HS, Pinho EC De, Kirchhof AL, Rocha LP, Costa Kerber NP Da. Workloads and inter‐relationships with the program for the humanization of prenatal and childbirth care. Cogitare Enfermagem 2018;23(3):e52801. [DOI: dx.doi.org/10.5380/ce.v23i3.52801]

[CD012392-bib-0011] Bradley EH, Byam P, Alpern R, Thompson JW, Zerihun A, Abeb Y, et al. A systems approach to improving rural care in Ethiopia. PLOS One 2012;7(4):e35042. [DOI: 10.1371/journal.pone.00350422013] PMC3338815 22558113

[CD012392-bib-0012] Cabral FB, Hirt LM, Sand IS Van der. Prenatal care from puerperal women’s point of view: from medicalization to the fragmentation of care. Revista da Escola de Enfermagem da U S P 2013;47(2):281‐7. 10.1590/s0080-62342013000200002 23743891

[CD012392-bib-0013] Cardelli AA, Marrero TL, Ferrari RA, Martins JT, Serafim D. Expectations and satisfaction of pregnant women: unveiling prenatal care in primary care. Investigación y Educación en Enfermería 2016;34(2):252‐60. [DOI: 10.17533/udea.lee.v34n2a04] 28569928

[CD012392-bib-0014] Chapman RR. Endangering safe motherhood in Mozambique: prenatal care as pregnancy risk. Social Science and Medicine 2003;57(2):355‐74. 10.1016/s0277-9536(02)00363-5 12765714

[CD012392-bib-0015] Chimezie RO. A Case Study of Primary Healthcare Services in Isu, Nigeria [Dissertation]. Minneapolis: Walden University, 2013. [Available from scholarworks.waldenu.edu/cgi/viewcontent.cgi?article=2056&context=dissertations ]

[CD012392-bib-0016] Choudhury N, Ahmed SM. Maternal care practices among the ultra‐poor households in rural Bangladesh: a qualitative exploratory study. BMC Pregnancy and Childbirth 2011;11:15. [DOI: 10.1186/1471-2393-11-15] PMC3056829 21362164

[CD012392-bib-0017] Chowdhury AM, Mahbub A, Chowdhury AS. Skilled attendance at delivery in Bangladesh: an ethnographic study. Research Monograph Series 2003; Vol. 22. [research.brac.net/monographs/mono_22.pdf ]

[CD012392-bib-0018] Conrad P, Allegri M De, Moses A, Larsson EC, Neuhann F, Müller O, et al. Antenatal care services in rural Uganda: missed opportunities for good‐quality care. Qualitative Health Research 2012;22(5):619‐29. 10.1177/1049732311431897 22232296

[CD012392-bib-0019] Coverston CR, Franklin CY, Davis JP. Seeking safe passage: Argentine women's experiences of prenatal care. Health Care for Women International 2004;25(7):620‐35. 10.1080/07399330490458132 15487481

[CD012392-bib-0020] Dako‐Gyeke P, Aikins M, Aryeetey R, Mccough L, Adongo PB. The influence of socio‐cultural interpretations of pregnancy threats on health seeking behavior among pregnant women in urban Accra, Ghana. BMC Pregnancy and Childbirth 2013;13:211. [www.biomedcentral.com/1471‐2393/13/211] 10.1186/1471-2393-13-211 PMC3840661 24246028

[CD012392-bib-0021] Castro ME De, Moura, MA, Silva LM Da. Quality of prenatal assistance: A perspective of the new puerpera’s. Revista da Rede de Enfermagem do Nordeste 2010;11:72‐81.

[CD012392-bib-0022] Docherty A, Bugge C, Watterson A. Engagement: an indicator of difference in the perceptions of antenatal care from pregnant women from diverse socioeconomic backgrounds. Health Expectations 2011;15(2):126‐38. 10.1111/j.1369-7625.2011.00684.x PMC5060614 21615639

[CD012392-bib-0023] Duarte SJ. Reasons that pregnant women visit prenatal care: a social representation study. Ciencia y Enfermería 2012;18(2):75‐82.

[CD012392-bib-0024] Earle S. Pregnancy and the maintenance of self‐identity: implications for antenatal care in the community. Health and Social Care in the Community 2000;8(4):235‐41. 10.1046/j.1365-2524.2000.00246.x 11560693

[CD012392-bib-0025] Family Care International. Care seeking during pregnancy, delivery and the post‐partum period: a study in Hombay and Migori districts, Kenya. www.familycareintl.org/UserFiles/File/SCI%20Kenya%20qualitative%20report.pdf 2003; Vol. (accessed 20 May 2019).

[CD012392-bib-0026] Franngard C, Hansveden A, Lijestrand J. Compassion and severe challenges: an exploratory study of being a midwife in rural Uganda. MIDIRS Midwifery Digest 2006;16(4):461‐6.

[CD012392-bib-0027] Ganle JK, Parker M, Fitzpatrick R, Otupiri E. A qualitative study of health system barriers to accessibility and utilization of maternal and newborn healthcare services in Ghana after user‐fee abolition. BMC Pregnancy and Childbirth 2014;14:425. [DOI: 10.1186/s12884-014-0425-8] PMC4307897 25527872

[CD012392-bib-0028] Gheibizadeh M, Ali Abedi H, Mohammadi E, Abedi P. Iranian women and care providers' perceptions of equitable prenatal care: A qualitative study. Nursing Ethics 2016;23(4):465‐77. 10.1177/0969733015573653 25802210

[CD012392-bib-0029] Graner S, Mogren I, Duong Le Q, Krantz G, Klingberg‐Allvin M. Maternal health care professionals’ perspectives on the provision and use of antenatal and delivery care: a qualitative descriptive study in rural Vietnam. BMC Public Health 2010;10:608. [DOI: 10.1186/1471-2458-10-608.; www.biomedcentral.com/1471‐2458/10/608 ] PMC3091560 20946681

[CD012392-bib-0030] Graner S, Klingberg‐Allvin M, Duong LQ, Krantz G, Mogren I. Pregnant women’s perception on signs and symptoms during pregnancy and maternal health care in a rural low‐resource setting. Acta Obstetricia et Gynecologica Scandinavica 2013;92(9):1094‐100. 10.1111/aogs.12170 23663254

[CD012392-bib-0031] Griffiths P, Stephenson R. Understanding users’ perspectives of barriers to maternal health care use in Maharashtra, India. Journal of Biosocial Science 2001;33(3):339‐59. 10.1017/s002193200100339x 11446398

[CD012392-bib-0032] Gross K, Schellenberg JA, Kessy F, Pfeiffer C, Obrist B. Antenatal care in practice: an exploratory study in antenatal care clinics in the Kilombero Valley, south eastern Tanzania. BMC Pregnancy and Childbirth 2011;11:36. [www.biomedcentral.com/1471‐2393/11/36 ] 10.1186/1471-2393-11-36 PMC3123249 21599900

[CD012392-bib-0033] Haddrill R, Jones GL, Mitchell CA, Anumba DO. Understanding delayed access to antenatal care: a qualitative interview study. BMC Pregnancy and Childbirth 2014;14:207. [www.biomedcentral.com/1471‐2393/14/207 ] 10.1186/1471-2393-14-207 PMC4072485 24935100

[CD012392-bib-0034] Heaman MI, Sword W, Elliott L, Moffat M, Helewa ME, Morris H, et al. Perceptions of barriers, facilitators and motivators related to use of prenatal care: a qualitative descriptive study of inner‐city women in Winnipeg, Canada. SAGE Open Medicine 2015;3:2050312115621314. [DOI: doi.org/10.1177/2050312115621314] 10.1177/2050312115621314 PMC4822530 27092262

[CD012392-bib-0035] Heberlein EC, Picklesimer AH, Billings DL, Covington‐Kolb S, Farber N, Frongillo EA. Qualitative comparison of womens perspectives on the functions and benefits of group and individual prenatal care. Journal of Midwifery & Women's Health 2016;61(2):224‐34. [DOI: 10.1111/jmwh.12379] 26878599

[CD012392-bib-0036] Hunter A, Devane D, Houghton C, Grealish A, Tully A, Smith V. Woman‐centred care during pregnancy and birth in Ireland: thematic analysis of women's and clinicians' experiences. BMC Pregnancy and Childbirth 2017;17:322. [DOI: 10.1186/s12884-017-1521-3] PMC5613376 28946844

[CD012392-bib-0037] Kabakian‐Khasholian T, Campbell O, Shediac‐Rizkallah M, Ghorayeb F. Women’s experiences of maternity care: satisfaction or passivity?. Social Science and Medicine 2000;51(1):103‐13. 10.1016/s0277-9536(99)00443-8 10817473

[CD012392-bib-0038] Khoso A, Khan AZ, Sayed SA, Rafique G. Perspectives regarding antenatal care, delivery and breastfeeding practices of women from Baluchistan, Pakistan. Journal of Ayub Medical College, Abbottabad 2016;28(1):105‐9. 27323573

[CD012392-bib-0039] Kraschnewski JL, Chuang CH, Poole ES, Peyton T, Blubaugh I, Pauli J, et al. Paging “Dr. Google”: does technology fill the gap created by the prenatal care visit structure? Qualitative Focus Group Study with pregnant women. Journal of Medical Internet Research 2014;16(6):e147. [DOI: 10.2196/jmir.3385] PMC4060145 24892583

[CD012392-bib-0040] Lagan BM, Sinclair M, Kernohan WG. What is the impact of the internet on decision making in pregnancy? a global study. Birth 2011;38(4):336‐45. 10.1111/j.1523-536X.2011.00488.x 22112334

[CD012392-bib-0041] Larsen GL, Lupiwa S, Kave HP, Gillieatt S, Alpers MP. Antenatal care in Goroka: issues and perceptions. Papua amd New Guinea Medical Journal 2004;47(3‐4):202‐14. 16862944

[CD012392-bib-0042] Larsson A, Warna‐Furu C, Nasman Y. The meaning of caring in prenatal care from Swedish women’s perspectives. Scandinavian Journal of Caring Sciences 2017;31(4):702‐9. [DOI: 10.1111/scs.12383] 27859526

[CD012392-bib-0043] Lasso Toro P. Prenatal care: tensions or possible routes between culture and health system. Pensamiento Psicológico 2012;10(2):123‐133.

[CD012392-bib-0044] Leal NJ, Barreiro MS, Mendes RB, Alves CK, Freitas C. Prenatal care: Nurses testimonial. Journal of Research: Fundamental Care Online 2018;10(1):113‐22. [DOI: dx.doi.org/10.9789/2175‐5361.2018.v10i1.113‐122]

[CD012392-bib-0045] LeMasters K, Wallis AB, Chereches R, Gichane M, Tehei C, Varga A, et al. Pregnancy experiences of women in rural Romania: understanding ethnic and socioeconomic disparities. Culture, Health & Sexuality 2018;21(3):249‐62. [DOI: 10.1080/13691058.2018.1464208] PMC6237651 29764305

[CD012392-bib-0046] Mahiti GR, Mkoka DA, Kiwara AD, Mbekenga CK, Hurtig AK, Goicolea I. Womens perceptions of antenatal delivery and postpartum services in rural Tanzania. Global Health Action 2015;8:28567. [DOI: dx.doi.org/10.3402/gha.v8.28567] 10.3402/gha.v8.28567 PMC4617868 26498576

[CD012392-bib-0047] Manithip C, Edin K, Sihavong A, Wahlstrom R, Wessel H. Poor quality of antenatal care services—Is lack of competence and support the reason? An observational and interview study in rural areas of Lao PDR. Midwifery 2013;29(3):195‐202. 10.1016/j.midw.2011.12.010 22776568

[CD012392-bib-0048] Maputle MS, Lebese RT, Khoza LB, Shilubane NH, Netshikweta LM. Knowledge and attitudes of pregnant women towards antenatal care services at Tshino village, Vhembe district, South Africa. African Journal for Physical, Health Education, Recreation and Dance 2013;March(Supplement 1):126‐37.

[CD012392-bib-0049] Mathole T, Linndmark G, Ahlberg BM. Dilemmas and paradoxes in providing and changing antenatal care: a study of nurses and midwives in rural Zimbabwe. Health Care Women International 2005;26(10):937‐56. 10.1093/heapol/czi046 16183736

[CD012392-bib-0050] Matsuoka S, Aiga H, Rasmey LC, Rathavy T, Okitsu A. Perceived barriers to utilization of maternal health services in rural Cambodia. Health Policy 2010;95(2‐3):255‐63. 10.1016/j.healthpol.2009.12.011 20060193

[CD012392-bib-0051] Mayca J, Palacios‐Flowers E, Medina A, Velasquez JE, Castañeda D. Perceptions of health personnel and the community in relation to the cultural appropriateness of maternal perinatal services in rural areas in the Andes and Amazon from Huanuco region. Revista Peruana de Medicina Experimental y Salud Publica 2009;26(2):145‐160.

[CD012392-bib-0052] McDonald SD, Sword W, Eryuzlu LE, Biringer AB. A qualitative descriptive study of the group prenatal care experience: perceptions of women with low‐risk pregnancies and their midwives. BMC Pregnancy and Childbirth 2014;14:334. [www.biomedcentral.com/1471‐2393/14/334 ] 10.1186/1471-2393-14-334 PMC4262064 25258167

[CD012392-bib-0053] McNeil DA, Vekved M, Dolan SM, Siever J, Horn S, Tough SC. Getting more than they realized they needed: a qualitative study of women's experience of group prenatal care. BMC Pregnancy and Childbirth 2012;12:17. [www.biomedcentral.com/1471‐2393/12/17 ] 10.1186/1471-2393-12-17 PMC3364900 22436393

[CD012392-bib-0054] Miteniece E, Pavlova M, Shengelia L, Rechel B, Groot W. Barriers to accessing adequate maternal care in Georgia: a qualitative study. BMC Health Services Research 2018;18:631. [DOI: doi.org/10.1186/s12913‐018‐3432‐z] 10.1186/s12913-018-3432-z PMC6090778 30103763

[CD012392-bib-0055] Molina G, Vargas G, Shaw A. Compromised quality of maternal healthcare in a market economy: Medellin, Colombia. Colombia Medica 2011;42(3):294‐302.

[CD012392-bib-0056] Mrisho M, Obrist B, Schellenberg JA, Haws RA, Mushi AK, Mshinda H, et al. The use of antenatal and postnatal care: perspectives and experiences of women and health care providers in rural southern Tanzania. BMC Pregnancy and Childbirth 2009;9:10. [DOI: 10.1186/1471-2393-9-10] PMC2664785 19261181

[CD012392-bib-0057] Mugo NS, Dibley MJ, Damundu EY, Alam A. Barriers faced by the health workers to deliver maternal care services and their perceptions of the factors preventing their clients from receiving the services: a qualitative study in South Sudan. Maternal and Child Health Journal 2018;22:1598‐606. [DOI: doi.org/10.1007/s10995‐018‐2555‐5] 10.1007/s10995-018-2555-5 29956127

[CD012392-bib-0058] Mumtaz Z, Salway SM. Gender, pregnancy and the uptake of antenatal care services in Pakistan. Sociology of Health and Illness 2007;29(1):1‐26. [DOI: 10.1111/j.1467-9566.2007.00519.x] 17286703

[CD012392-bib-0059] Munguambe K, Boene H, Vidler M, Bique C, Sawchuck D, Firoz T, et al. Barriers and facilitators to health care seeking behaviours in pregnancy in rural communities of southern Mozambique. Reproductive Health 2016;13(Suppl 1):31. [DOI: 10.1186/s12978-016-0141-0] PMC4943506 27356968

[CD012392-bib-0060] Myer L, Harrison A. Why do women seek antenatal care late? Perspectives from rural South Africa. Journal of Midwifery & Women's Health 2003;48(4):268‐72. 10.1016/s1526-9523(02)00421-x 12867911

[CD012392-bib-0061] Neves PR, Salim N, Soares GCF, Gualda DM. Experiences of women in a pregnant group: a descriptive study. Online Brazilian Journal of Nursing [Internet] 2013;12(4):862‐71. [DOI: dx.doi.org/10.5935/1676‐4285.20134143; www.objnursing.uff.br/index.php/nursing/article/view/4143]

[CD012392-bib-0062] Novick G, Sadler LS, Kennedy HP, Cohen SS, Groce NE, Knafl KA. Women's experience of group prenatal care. Qualitative Health Research 2011;21(1):97‐116. 10.1177/1049732310378655 PMC3085399 20693516

[CD012392-bib-0063] Novick G, Sadler LS, Knafl KA, Groce NE, Kennedy HP. In a hard spot: Providing group prenatal care in two urban clinics. Midwifery 2013;29(6):690‐7. [DOI: 10.1016/j.midw.2012.06.013] PMC3498538 22884892

[CD012392-bib-0064] Pretorius CF, Greeff M. Health‐service utilization by pregnant women in the greater Mafikeng‐Mmbatho district. Curationis 2004;27(1):72‐81. 10.4102/curationis.v27i1.959 15168627

[CD012392-bib-0065] Rahmani Z, Brekke M. Antenatal and obstetric care in Afghanistan – a qualitative study among health care receivers and health care providers. BMC Health Services Research 2013;13:166. [www.biomedcentral.com/1472‐6963/13/166 ] 10.1186/1472-6963-13-166 PMC3654902 23642217

[CD012392-bib-0066] Rath S, Nair N, Tripathy PK, Barnett S, Rath S, Mahapatra R, et al. Explaining the impact of a women’s group led community mobilization intervention on maternal and newborn health outcomes: the Ekjut trial process evaluation. BMC International Health and Human Rights 2010;10:25. [www.biomedcentral.com/1472‐698X/10/25] 10.1186/1472-698X-10-25 PMC2987759 20969787

[CD012392-bib-0067] Saftner MA, Neerland C, Avery MD. Enhancing women's confidence for physiologic birth: Maternity care providers’ perspectives. Midwifery 2017;53:28‐34. [http://dx.doi.org/10.1016/j.midw.2017.07.012] 10.1016/j.midw.2017.07.012 28743051

[CD012392-bib-0068] Santos AL, Radovanovic CA, Marcon SS. Prenatal care: satisfaction and expectations. Revista da Rede de Enfermagem do Nordeste 2010;11:61‐71.

[CD012392-bib-0069] Shabila HP, Ahmed HM, Yasin MY. Women’s views and experiences of antenatal care in Iraq: a Q methodology study. BMC Pregnancy and Childbirth 2014;14:43. [www.biomedcentral.com/1471‐2393/14/43] 10.1186/1471-2393-14-43 PMC3902000 24450437

[CD012392-bib-0070] Simkhada B, Porter MA, Teijlingen ER Van. The role of mothers‐in‐law in antenatal care decision‐making in Nepal: a qualitative study. BMC Pregnancy and Childbirth 2010;10:34. [DOI: 10.1186/1471-2393-10-34] PMC2910658 20594340

[CD012392-bib-0071] Spindola T, Progianti JM, Garcia Penna LH. Pregnant women’s experiences about the obstetrics nurse support during prenatal care at a university hospital. Ciencia y Enfermería 2012;18(2):65‐73.

[CD012392-bib-0072] Stokes E, Dumbaya I, Owens S, Brabin L. The right to remain silent: a qualitative study of the medical and social ramifications of pregnancy disclosure for Gambian women. BJOG 2008;115(13):1641‐7. [DOI: 10.1111/j.1471-0528.2008.01950.x] 19035940

[CD012392-bib-0073] Sword W. Prenatal care use among women of low income: a matter of "taking care of self". Qualitative Health Research 2003;13(3):319. [DOI: 10.1177/0095399702250128] 12669334

[CD012392-bib-0074] Sword W, Heaman MI, Brooks S, Tough S, Janssen PA, Young D, et al. Women’s and care providers’ perspectives of quality prenatal care: a qualitative descriptive study. BMC Pregnancy and Childbirth 2012;12:29. [www.biomedcentral.com/1471‐2393/12/29] 10.1186/1471-2393-12-29 PMC3352181 22502640

[CD012392-bib-0075] Sychareun V, Somphet V, Chaleunvong K, Hansana V, Phengsavanh A, Xayavong S, et al. Perceptions and understandings of pregnancy, antenatal care and postpartum care among rural Lao women and their families. BMC Pregnancy and Childbirth 2016;16:245. [DOI: 10.1186/s12884-016-1031-8] PMC4997701 27561359

[CD012392-bib-0076] Teate A, Leap N, Rising SS, Homer CS. Women’s experiences of group antenatal care in Australia ‐ the Centering Pregnancy Pilot Study. Midwifery 2011;27(2):138‐45. [DOI: 10.1016/j.midw.2009.03.001] 19386402

[CD012392-bib-0077] Teate A, Leap N, Homer CS. Midwives’ experiences of becoming Centering Pregnancy facilitators: a pilot study in Sydney, Australia. Women and Birth 2013;26:e31‐36. 10.1016/j.wombi.2012.08.002 22926224

[CD012392-bib-0078] Thwala SB, Jones LK, Holroyd E. Swaziland rural maternal care: Ethnography of the interface of custom and biomedicine. International Journal of Nursing Practice 2011;17(1):93‐101. [DOI: 10.1111/j.1440-172X.2010.01911.x] 21251159

[CD012392-bib-0079] Titaley CR, Hunter CL, Heywood P, Dibley MJ. Why don’t some women attend antenatal and postnatal care services?: a qualitative study of community members’ perspectives in Garu, Sukambi and Ciamis districts of West Java Province, Indonesia. BMC Pregnancy and Childbirth 2010;10:61. [DOI: 10.1186/1471-2393-10-61.] PMC2964562 20937146

[CD012392-bib-0080] Umeora OU, Ejikeme BN, Sunday‐Adeoye I, Ogu RN. Implementing the new WHO antenatal care model: voices from end‐users in a rural Nigerian community. Nigerian Journal of Clinical Practice 2008;11(3):260‐4. 19140366

[CD012392-bib-0081] Walburg V, Friederich F, Callahan S. Embarrassment and modesty feelings during pregnancy, childbirth and follow‐up care: A qualitative approach. Journal of Reproductive and Infant Psychology 2014;32(2):126‐36. [DOI: dx.doi.org/10.1080/02646838.2013.860518]

[CD012392-bib-0082] Wilmore M, Rodger D, Humphreys S, Clifton VL, Dalton J, Flabouris M, et al. How midwives tailor health information used in antenatal care. Midwifery 2015;31(1):74‐99. [DOI: dx.doi.org/10.1016/j.midw.2014.06.004] 10.1016/j.midw.2014.06.004 25017172

[CD012392-bib-0083] Worley BL, Bullock LF, Geden E. The incubator model: is it effective prenatal care?. Clinical Excellence for Nurse Practitioners 2004;8(1):29‐34.

[CD012392-bib-0084] Wright D, Pincombe J, McKellar L. Exploring routine hospital antenatal care consultations — An ethnographic study. Women and Birth 2018;31(3):e162‐e169. [DOI: 10.1016/j.wombi.2017.09.010] 28969996

[CD012392-bib-0085] Østergaard LR. Maternal healthcare in context: A qualitative study of women's tactics to improve their experience of public healthcare in rural Burkina Faso. Social Science & Medicine 2015;147:98‐104. [DOI: dx.doi.org/10.1016/j.socscimed.2015.10.062] 10.1016/j.socscimed.2015.10.062 26560408

[CD012392-bib-0086] Lohmann S, Mattern E, Ayerle GM. Midwives' perceptions of women's preferences related to midwifery care in Germany: A focus group study. Midwifery 2018;61:53‐62. [https://doi.org/10.1016/j.midw.2018.02.005] 10.1016/j.midw.2018.02.005 29529579

[CD012392-bib-0087] Murira N, Lutzen K, Lindmark G, Christensson K. Communication patterns between healthcare providers and their clients at an antenatal clinic in Zimbabwe. Health Care for Women International 2003;24(2):83‐92. [DOI: 10.1080/07399330390170060] 12746018

[CD012392-bib-0088] Nigenda G, Langer A, Kuchaisit C, Romero M, Rojas G, Al‐Osimy M, et al. Womens' opinions on antenatal care in developing countries: results of a study in Cuba, Thailand, Saudi Arabia and Argentina. BMC Public Health 2003;3:17. [www.biomedcentral.com/1471‐2458/3/17] 10.1186/1471-2458-3-17 PMC166129 12756055

[CD012392-bib-0089] Påfs J, Musafili A, Binder‐Finnema P, Klingberg‐Allvin M, Rulisa S, Essén B. ‘They would never receive you without a husband’: Paradoxical barriers to antenatal care scale‐up in Rwanda. Midwifery 2015;31(12):1149‐56. [DOI: dx.doi.org/10.1016/j.midw.2015.09.010] 10.1016/j.midw.2015.09.010 26471934

[CD012392-bib-0090] Pell C, Menaca A, Were F, Afrah NA, Chatio S, Manda‐Taylor L, et al. Factors affecting antenatal care attendance: results from qualitative studies in Ghana, Kenya and Malawi. PLoS One 8;1:e53747. [DOI: 10.1371/journal.pone.0053747] PMC3546008 23335973

[CD012392-bib-0091] Tsawe M, Susuman AS. Determinants of access to and use of maternal health care services in the Eastern Cape, South Africa: a quantitative and qualitative investigation. BMC Research Notes 2014;7:723. [www.biomedcentral.com/1756‐0500/7/723] 10.1186/1756-0500-7-723 PMC4203863 25315012

[CD012392-bib-0092] Aikawa Y. Positive support and negative support from medical staff which perinatal women experienced. Journal of the Japanese Academy of Midwives 2004;18(2):34‐43.

[CD012392-bib-0093] Al‐Amer R, Ramjan L, Glew P, Darwish M, Salamonson Y. Translation of interviews from a source language to a target language: examining issues in cross‐cultural health care research. Journal of Clinical Nursing 2015;24(9‐10):1151‐62. 10.1111/jocn.12681 25181257

[CD012392-bib-0094] Ames HM, Glenton C, Lewin S. Parents' and informal caregivers' views and experiences of communication about routine childhood vaccination: a synthesis of qualitative evidence. Cochrane Database of Systematic Reviews 2017, Issue 2. [DOI: 10.1002/14651858.CD011787.pub2] PMC5461870 28169420

[CD012392-bib-0095] Azjen I. The theory of planned behaviour. Organizational Behavior and Human Decision Processes 1991;50(2):179‐211.

[CD012392-bib-0096] Baig LA, Shaikh S, Polkowski M, Ali SK, Jamali S, Mazharullah L, et al. Violence against health care providers: a mixed‐methods study from Karachi, Pakistan. Journal of Emergency Medicine 2018;54(4):558‐66. 10.1016/j.jemermed.2017.12.047 29449119

[CD012392-bib-0097] Becker S, Mlay R, Schwandt HM, Lyamuya E. Comparing couples' and individual voluntary counseling and testing for HIV at antenatal clinics in Tanzania: a randomized trial. AIDS and Behavior 2010;14(3):558‐66. 10.1007/s10461-009-9607-1 19763813

[CD012392-bib-0098] Benova L, Tunçalp Ö, Moran AC, Campbell OM. Not just a number: examining coverage and content of antenatal care in low‐income and middle‐income countries. BMJ Global Health 2018; Vol. 3, issue 2:000779. [e000779. doi:10.1136/ bmjgh‐2018‐000779] 10.1136/bmjgh-2018-000779 PMC5898334 29662698

[CD012392-bib-0099] Birbili M. Translating from one language to another. Social Research Update 2000;31:1‐7.

[CD012392-bib-0100] Biza A, Jille‐Traas I, Colomar M, Belizan M, Requejo Harris J, Crahay B, et al. Challenges and opportunities for implementing evidence‐based antenatal care in Mozambique: a qualitative study. BMC Pregnancy and Childbirth. 2015;15:200. [DOI: 10.1186/s12884-015-0625-x.] PMC4557743 26330022

[CD012392-bib-0101] Bohren MA, Hunter EC, Munthe‐Kaas HM, Souza JP, Vogel JP, Gülmezoglu AM. Facilitators and barriers to facility‐based delivery in low‐ and middle‐income countries: a qualitative evidence synthesis. Reproductive Health 2014;11(1):71. [DOI: 10.1186/1742-4755-11-71; www.reproductive‐health‐journal.com/content/11/1/71] PMC4247708 25238684

[CD012392-bib-0102] Bohren MA, Vogel JP, Hunter EC, Lutsiv O, Makh SK, Souza JP, et al. The mistreatment of women during childbirth in health facilities globally: a mixed‐methods systematic review. PLoS Medicine 2015;12(6):e1001847. 10.1371/journal.pmed.1001847 PMC4488322 26126110

[CD012392-bib-0103] Booth A. Chapter 3: Searching for studies. In: Noyes J, Booth A, Hannes K, Harden A, Harris J, Lewin S, et al, editor(s). Supplementary Guidance for Inclusion of Qualitative Research in Cochrane Systematic Reviews of Interventions. Cochrane Collaboration Qualitative Methods Group 2011.

[CD012392-bib-0104] Booth A, Carroll C. How to build up the actionable knowledge base: the role of 'best fit' framework synthesis for studies of improvement in healthcare. BMJ Quality & Safety 2015;24(11):700‐8. [doi: 10.1136/bmjqs‐2014‐003642] 10.1136/bmjqs-2014-003642 PMC4680127 26306609

[CD012392-bib-0105] Booth A, Noyes J, Flemming K, Gerhardus A, Wahlster P, Wilt GJ Van Der, et al. Guidance on choosing qualitative evidence synthesis methods for use in health technology assessments of complex interventions. Available from: www.integrate‐hta.eu/downloads/ 2016 (Accessed May 16th 2018).

[CD012392-bib-0106] Bowser D, Hill K. Exploring evidence for disrespect and abuse in facility‐based childbirth. www.tractionproject.org/sites/default/files/Respectful_Care_at_Birth_9‐20‐101_Final.pdf (accessed prior to 17 September 2016).

[CD012392-bib-0107] Brown HC, Smith HJ, Mori R, Noma H. Giving women their own case notes to carry during pregnancy. Cochrane Database of Systematic Reviews 2015, Issue 10. [DOI: 10.1002/14651858.CD002856.pub3] PMC7054050 26465209

[CD012392-bib-0108] Carlson NS, Lowe NK. Centering Pregnancy: a new approach in prenatal care. American Journal of Maternal Child Nursing 2006;31(4):218‐23. 10.1097/00005721-200607000-00004 16940816

[CD012392-bib-0109] Carolan M. Health literacy and the information needs and dilemmas of first‐time mothers over 35 years. Journal of Clinical Nursing 2007;16(6):1162‐72. 10.1111/j.1365-2702.2007.01600.x 17518891

[CD012392-bib-0110] Carter EB, Temming LA, Akin J, Fowler S, Macones GA, Colditz GA. Group prenatal care compared with traditional prenatal care: a systematic review and meta‐analysis. Obstetrics and Gynecology 2016;128(3):551‐61. 10.1097/AOG.0000000000001560 PMC4993643 27500348

[CD012392-bib-0111] Catling CJ, Medley N, Foureur M, Ryan C, Leap N, Teate A, et al. Group versus conventional antenatal care for women. Cochrane Database of Systematic Reviews 2015, Issue 2. [DOI: 10.1002/14651858.CD007622.pub3] PMC6465187 25922865

[CD012392-bib-0112] Clark L, Birkhead AS, Fernandez C, Egger MJ. A transcription and translation protocol for sensitive cross‐cultural team research. Qualitative Health Research 2017;27(12):1751‐64. [doi: 10.1177/1049732317726761] 10.1177/1049732317726761 PMC5642906 28936930

[CD012392-bib-0113] Downe S, Simpson L, Trafford K. Expert intrapartum maternity care: a meta‐synthesis. Journal of Advanced Nursing 2007;57(2):127‐40. 10.1111/j.1365-2648.2006.04079.x 17214749

[CD012392-bib-0114] Downe S, Finlayson K, Walsh D, Lavender T. 'Weighing up and balancing out': a meta‐synthesis of barriers to antenatal care for marginalised women in high‐income countries. BJOG 2009;116(4):518‐29. 10.1111/j.1471-0528.2008.02067.x 19250363

[CD012392-bib-0115] Downe S, Finlayson K, Tunçalp Ö, Gülmezoglu M. What matters to women: A scoping review to identify the processes and outcomes of antenatal care provision that are important to healthy pregnant women. BJOG 2016;123(4):529‐39. 10.1111/1471-0528.13819 26701735

[CD012392-bib-0116] Downe S, Finlayson K, Oladapo O, Bonet M, Gülmezoglu AM. What matters to women during childbirth: A systematic qualitative review. PLoS One 2018;13(4):e0194906. [e0194906.] 10.1371/journal.pone.0194906 PMC5903648 29664907

[CD012392-bib-0117] Dowswell T, Carroli G, Duley L, Gates S, Gülmezoglu AM, Khan‐Neelofur D, et al. Alternative versus standard packages of antenatal care for low‐risk pregnancy. Cochrane Database of Systematic Reviews 2015, Issue 7. [DOI: 10.1002/14651858.CD000934.pub3] PMC7061257 26184394

[CD012392-bib-0118] Elbarazi I, Loney T, Yousef S, Elias A. Prevalence of and factors associated with burnout among health care professionals in Arab countries: a systematic review. MC Health Services Research 2017;17(1):491‐501. [DOI: 10.1186/s12913-017-2319-8] PMC5513024 28716142

[CD012392-bib-0119] Ezeonwu M. Policy strategies to improve maternal health services delivery and outcomes in Anambra State, Nigeria. Health Care for Women International 2014;35(7‐9):828‐44. [DOI: 10.1080/07399332.2014.925454] 24911182

[CD012392-bib-0120] Finlayson K, Downe S. Why do women not use antenatal services in low‐ and middle‐income countries? A meta‐synthesis of qualitative studies. PLOS Medicine 2013;10(1):e1001373. 10.1371/journal.pmed.1001373 PMC3551970 23349622

[CD012392-bib-0121] Fishbein M, Ajzen I. Predicting and Changing Behavior: The Reasoned Action Approach. New York: Taylor & Francis, 2010.

[CD012392-bib-0122] Gale NK, Heath G, Cameron E, Rashid S, Redwood S. Using the framework method for the analysis of qualitative data in multi‐disciplinary health research. BMC Medical Research Methodology 2013;13:117. 10.1186/1471-2288-13-117 PMC3848812 24047204

[CD012392-bib-0123] Karlström A, Nystedt A, Hildingsson I. The meaning of a very positive birth experience: focus groups discussions with women. BMC Pregnancy and Childbirth 2015;15:251. [DOI: 10.1186/s12884-015-0683-0] PMC4600272 26453022

[CD012392-bib-0124] Khalil DD. Nurses' attitude towards ‘difficult’ and ‘good’ patients in eight public hospitals. International Journal of Nursing Practice 2009;15:437‐43.

[CD012392-bib-0125] Lehmann U, Dieleman M, Martineau T. Staffing remote rural areas in middle‐ and low‐income countries: a literature review of attraction and retention. BMC Health Services Research 2008;23(8):19. [DOI: 10.1186/1472-6963-8-19] PMC2259330 18215313

[CD012392-bib-0126] Lewin S, Glenton C, Munthe‐Kaas H, Carlsen B, Colvin CJ, Gülmezoglu M, et al. Using qualitative evidence in decision making for health and social interventions: an approach to assess confidence in findings from qualitative evidence syntheses (GRADE‐CERQual). PLoS Medicine 2015;12(10):e1001895. 10.1371/journal.pmed.1001895 PMC4624425 26506244

[CD012392-bib-0127] Lincoln YS, Guba EG. Naturalistic Inquiry. Newbury Park (CA): Sage Publication, 1985.

[CD012392-bib-0128] Magriples U, Boynton MH, Kershaw TS, Lewis J, Rising SS, Tobin JN, et al. The impact of group prenatal care on pregnancy and postpartum weight trajectories. American Journal of Obstetrics and Gynecology 2015;213(5):688.e1‐9. [DOI: 10.1016/j.ajog.2015.06.066] PMC4910388 26164694

[CD012392-bib-0129] Mbuagbaw L, Medley N, Darzi AJ, Richardson M, Habiba Garga K, Ongolo‐Zogo P. Health system and community level interventions for improving antenatal care coverage and health outcomes. Cochrane Database of Systematic Reviews 2015, Issue 12. [DOI: 10.1002/14651858.CD010994.pub2] PMC4676908 26621223

[CD012392-bib-0130] Merriam S. Qualitative Research: A Guide to Design and Implementation. San Francisco (CA): Jossey‐Bass, 2009.

[CD012392-bib-0131] Munabi‐Babigumira S, Glenton C, Lewin S, Fretheim A, Nabudere H. Factors that influence the provision of intrapartum and postnatal care by skilled birth attendants in low‐ and middle‐income countries: a qualitative evidence synthesis. Cochrane Database of Systematic Reviews 2017, Issue 11. [DOI: 10.1002/14651858.CD011558.pub2] PMC5721625 29148566

[CD012392-bib-0132] National Institute for Health and Care Excellence. Antenatal care for uncomplicated pregnancies. www.nice.org.uk/guidance/CG62 (accessed 7 September 2015).

[CD012392-bib-0133] Nie JB. Non‐medical sex‐selective abortion in China: ethical and public policy issues in the context of 40 million missing females. British Medical Bulletin 2011;98:7‐20. [DOI: 10.1093/bmb/ldr015] 21596712

[CD012392-bib-0134] Noblit GW, Hare RD. Meta‐Ethnography: Synthesizing Qualitative Studies. Newbury Park (CA): Sage, 1988.

[CD012392-bib-0135] Nyman V, Bondas T, Downe S, Berg M. Glancing beyond or being confined to routines: labour ward midwives' responses to change as a result of action research. Midwifery 2013;29(6):573‐8. [DOI: 10.1016/j.midw.2013.02.010] 23566557

[CD012392-bib-0136] Olivier de Sardan OJ, Diarra A, Moha M. Travelling models and the challenge of pragmatic contexts and practical norms: the case of maternal health. Health Research Policy and Systems / BioMed Central 2017;12(15 (Suppl)):60. [DOI: 10.1186/s12961-017-0213-9] PMC5516842 28722553

[CD012392-bib-0137] Pawson R, Tilley N. Caring communities, paradigm polemics, design debates. Evaluation 1998;4:73‐90.

[CD012392-bib-0138] Phillippi JC. Women's perceptions of access to prenatal care in the United States: a literature review. Journal of Midwifery & Women's Health 2009;54(3):219‐25. 10.1016/j.jmwh.2009.01.002 19410214

[CD012392-bib-0139] Potts H, Hunt P. Participation and the right to the highest attainable standard of health. www.repository.essex.ac.uk/9714/ (accessed prior to 17 September 2016).

[CD012392-bib-0140] Regmi K, Naidoo J, Pilkington P. Understanding the processes of translation and transliteration in qualitative research. International Journal of Qualitative Methods 2010;9(1):16‐26.

[CD012392-bib-0141] Royal College of Midwives. State of Maternity Services Report. www.rcm.org.uk/sites/default/files/RCM%20State%20of%20Maternity%20Services%20Report%202015.pdf. 2015 accessed 16 May 2018).

[CD012392-bib-0142] Sandall J, Soltani H, Gates S, Shennan A, Devane D. Midwife‐led continuity models versus other models of care for childbearing women. Cochrane Database of Systematic Reviews 2016, Issue 4. [DOI: 10.1002/14651858.CD004667.pub5] PMC8663203 27121907

[CD012392-bib-0143] Seward N, Neuman M, Colbourn T, Osrin D, Lewycka S, Azad K, et al. Effects of women's groups practising participatory learning and action on preventive and care‐seeking behaviours to reduce neonatal mortality: A meta‐analysis of cluster‐randomised trials. PLoS One 2017;14:12. [e1002467] 10.1371/journal.pmed.1002467 PMC5716527 29206833

[CD012392-bib-0144] Smith A, Dixon A. Health care professionals’ views about safety in maternity services. Kings Fund, Maternity Services Inquiry. www.kingsfund.org.uk/sites/default/files/pro_evidence.pdf 2008 (accessed 29 May 2018).

[CD012392-bib-0145] Stevelink SA, Brakel WH Van. The cross‐cultural equivalence of participation instruments: a systematic review. Disability and Rehabilitation 2013;35(15):1256‐68. 10.3109/09638288.2012.731132 23789790

[CD012392-bib-0146] Thaddeus S, Maine D. Too far to walk? Maternal mortality in context. Social Science and Medicine 1994;38(8):1091‐110. 10.1016/0277-9536(94)90226-7 8042057

[CD012392-bib-0147] Till SR, Everetts D, Haas DM. Incentives for increasing prenatal care use by women in order to improve maternal and neonatal outcomes. Cochrane Database of Systematic Reviews 2015, Issue 12. [DOI: 10.1002/14651858.CD009916.pub2] PMC8692585 26671418

[CD012392-bib-0148] United Nations. Millenium Development Goals Report 2014. www.un.org/millenniumgoals/2014%20MDG%20report/MDG%202014%20English%20web.pdf. United Nations, (accessed prior to 17 September 2016).

[CD012392-bib-0149] United Nations. The Sustainable Development Goals Report. unstats.un.org/sdgs/report/2018 2018 (accessed 20 May 2019).

[CD012392-bib-0150] Villar J, Ba’aqeel H, Piaggio G, Lumbiganon P, Belizán JM, Farnot U, et al. WHO Antenatal Care Trial Research Group. WHO antenatal care randomised trial for the evaluation of a new model of routine antenatal care. Lancet 2001;357(9268):1551‐64. 10.1016/s0140-6736(00)04722-x 11377642

[CD012392-bib-0151] Vogel JP, Habib NA, Souza JP, Gülmezoglu AM, Dowswell T, Carroli G, et al. Antenatal care packages with reduced visits and perinatal mortality: a secondary analysis of the WHO Antenatal Care Trial. Reproductive Health 2013;10:19. 10.1186/1742-4755-10-19 PMC3637102 23577700

[CD012392-bib-0152] Walsh D, Downe S. Appraising the quality of qualitative research. Midwifery 2006;22(2):108‐19. 10.1016/j.midw.2005.05.004 16243416

[CD012392-bib-0153] World Health Organization. Provision of effective antenatal care ‐ Integrated Management of Pregnancy and Childbirth (IMPAC): Standards for maternal and neonatal care. www.who.int/reproductivehealth/publications/maternal_perinatal_health/effective_antenatal_care.pdf. WHO, (accessed prior to 17 September 2016).

[CD012392-bib-0154] World Health Organization. WHO recommendations on antenatal care for a positive pregnancy experience. www.who.int/reproductivehealth/publications/maternal_perinatal_health/anc‐positive‐pregnancy‐experience/en/ 2016 (accessed 16 May 2018). 28079998

[CD012392-bib-0155] Downe S, Finlayson K, Tunçalp Ö, Gülmezoglu AM. Factors that influence the uptake of routine antenatal services by pregnant women: a qualitative evidence synthesis. Cochrane Database of Systematic Reviews 2016, Issue 10. [DOI: 10.1002/14651858.CD012392] PMC6564082 31194903

[CD012392-bib-0156] Downe S, Finlayson K, Tunçalp Ö, Gülmezoglu AM. Factors that influence the provision of good‐quality routine antenatal services: a qualitative evidence synthesis of the views and experiences of maternity care providers. Cochrane Database of Systematic Reviews 2017, Issue 12. [DOI: 10.1002/14651858.CD012752.pub2]

